# Telomere length and cognitive changes in 7,877 older UK adults of European ancestry

**DOI:** 10.3389/fragi.2024.1480326

**Published:** 2024-11-01

**Authors:** Amy Packer, Leena Habiballa, Esteban Tato-Barcia, Gerome Breen, Helen Brooker, Anne Corbett, Ryan Arathimos, Clive Ballard, Adam Hampshire, Abbie Palmer, Danai Dima, Dag Aarsland, Byron Creese, Margherita Malanchini, Timothy R. Powell

**Affiliations:** ^1^ Social Genetic and Developmental Psychiatry Centre, Institute of Psychiatry, Psychology, and Neuroscience, King’s College London, London, United Kingdom; ^2^ NIHR Maudsley Biomedical Research Centre, King’s College London, London, United Kingdom; ^3^ University of Exeter Medical School, University of Exeter, Exeter, United Kingdom; ^4^ College of Medicine and Health, St Luke’s Campus, University of Exeter, Exeter, United Kingdom; ^5^ Department of Neuroimaging, Institute of Psychiatry, Psychology and Neuroscience, King’s College London, London, United Kingdom; ^6^ Department of Psychology, School of Health and Psychological Sciences, City, University of London, London, United Kingdom; ^7^ Department of Old Age Psychiatry, Institute of Psychiatry, Psychology and Neuroscience, King’s College London, London, United Kingdom; ^8^ Department of Life Sciences, College of Health, Medicine and Life Sciences, Brunel University London, London, United Kingdom; ^9^ Department of Biological and Experimental Psychology, School of Biological and Chemical Sciences, Queen Mary University of London, London, United Kingdom

**Keywords:** telomere length, cognitive function, polygenic score, ageing, PROTECT study

## Abstract

**Background:**

Telomere length (TL) has been linked to cognitive function, decline and dementia. This study aimed to explore whether both measured TL and genetic disposition for TL predict dimensions of cognitive performance in a longitudinal sample of older UK adults.

**Methods:**

We analysed data from PROTECT study participants aged ≥50 years without a dementia diagnosis, who had completed longitudinal cognitive testing. We calculated polygenic scores for telomere length (PGS-TL) for 7,877 participants and measured relative telomere length (RTL) in a subgroup of 846 participants using DNA extracted from saliva samples collected within 6 months either side of their baseline cognitive testing. Latent growth models were used to examine whether RTL and PGS-TL predict both baseline and longitudinal changes in cognitive performance (4 time-points, annually).

**Results:**

In the whole sample, we did not observe significant associations between either measure of telomere length and initial or longitudinal changes in cognitive performance. Stratifying by median age, in older adults (≥ ∼62 years), longer baseline RTL showed a nominal association with poorer baseline verbal reasoning performance (*n* = 423, *M*_*intercept*_ = 47.58, *B* = −1.05, *p* = .011) and PGS-TL was associated with performance over time (*n* = 3,939; slope factor, *M*_*slope*_ = 3.23, *B* = −0.45, *p* = .001; *slope* ^*2*^ factor, *M*_*slope*_^*2*^ = 0.21, *B* = 0.13, *p* = .002).

**Conclusion:**

Our findings suggest either the absence of a significant relationship between telomere length (RTL and PGS-TL) and cognitive performance (baseline and change over time), or possibly a weak age-dependent and domain-specific relationship, in older adults of European ancestry. More research is needed in representative and ancestrally diverse samples over a longer assessment period. Alternative biological ageing indicators may still provide utility in the early detection of individuals at risk for cognitive decline (e.g., pace-of ageing epigenetic clocks).

## 1 Introduction

Telomere length is a key biological hallmark of ageing (López-Otín et al., 2023). Premature telomere shortening (and cellular ageing) has been identified as one potential contributor to differences in cognitive function and decline in older adults (e.g., Hägg et al., 2017). Telomeres are DNA–protein complexes that cap the ends of chromosomes (Blackburn, 1991). The DNA component, stretches of tandem TTAGGG nucleotide repeats, represents sacrificial non-coding DNA elements that protect vital coding DNA from being lost during cell division, as a result of the “end replication problem” (see, Sfeir and De Lange, 2012). When a critically short telomere length is reached, it triggers a DNA damage response mechanism, which instigates the initiation of cellular senescence, whereby the cell can no longer divide and replace old or damaged cells (Blackburn et al., 2015). Telomere shortening therefore limits the proliferative capacity of cells, which may affect neural stem cells that give rise to glial cells throughout the brain, or neurons within the dentate gyrus of the hippocampus (Palmos et al., 2020).

Telomere length is often approximated using leukocyte or salivary DNA, which is easy to extract and correlated with telomere length in other tissues (Demanelis et al., 2020). In a systematic review and meta-analysis using data from 27 observational studies of individuals without dementia, Gampawar et al. (2022) found that leukocyte telomere length was associated with marginally better global cognition (β = 0.01; 95%CI: 0.00–0.02, *p* = .029, *N* = 19,609), as well as larger total brain volume (β = 0.43, 95%CI: 0.36%–0.50%, *p* = .008, *N* = 1,102). Furthermore, a longitudinal study in 2,734 older individuals revealed that longer telomere length at baseline predicted less decline 7 years later on the Modified Mini-Mental State Exam compared to individuals with medium or short telomere length (−1.7 points vs. −2.5 and −2.9 respectively, *p* = .01; mean points at baseline = 91) (Yaffe et al., 2011).

Most inter-individual differences in telomere length are present from birth, whereby individuals born with longer telomeres generally continue to have longer telomeres than their peers into adulthood (Martens et al., 2021). However, several potentially modifiable factors have been identified that may influence telomere length, including physical activity and educational attainment (Bountziouka et al., 2022; Sánchez-González et al., 2024). Furthermore, telomere length (leukocyte) has a strong inherited genetic component in humans. Meta-analytic estimates of twin studies suggest a heritability of 70% (range from 34% to 82%) (Broer et al., 2013). Further, the most powerful genome-wide association study (GWAS) to date (*N* = 464,716 participants aged 40–69 years from the UK Biobank; Codd et al., 2021), identified 197 independent sentinel variants demonstrating that telomere length is a polygenic trait with a genome-wide single nucleotide polymorphisms (SNP) heritability estimate of 8.1% (*SD* = 0.26).

To date, few studies have investigated the relationship between genetically predicted telomere length, cognitive function and age-related change. Mendelian randomisation studies have suggested a possible causal relationship between telomere length and cognitive function and decline (Chen et al., 2023; Hägg et al., 2017; Zhan et al., 2015). However, findings are inconsistent, with other Mendelian randomization studies reporting no association between telomere length and cognitive outcomes (Demanelis et al., 2021; Kuo et al., 2019). Building on previous research, we use polygenic scoring to estimate individuals’ lifelong genetic propensity for longer/shorter telomere length, by applying summary statistics from the largest GWAS of telomere length to date (i.e., Codd et al., 2021). Polygenic scores summarise the genetic influence on a target trait (e.g., telomere length) by aggregating the effects of trait-associated common variants emerging from GWAS into a single composite index (Dudbridge, 2013). Here, we employ an expansive PGS, using many SNPs to assess the proportion of variance in cognitive performance explained by genetic factors common to telomere length.

Our study aimed to examine associations between telomere length and longitudinal performance on four cognitive tests (paired associate learning, digit span, self-ordered search, verbal reasoning), in a large cohort of individuals aged ≥50 years without dementia at baseline assessment from the PROTECT study (https://www.protectstudy.org.uk/). Specifically, telomere length, genetically predicted (*n* = 7,877) and measured (*n* = 846), was assessed for its association with initial cognitive level and changes in cognitive performance over time (4 time-points, annually), using latent growth models. We hypothesised that longer telomere length (measured and captured by a PGS-TL) would be related to better performance on the cognitive tests at baseline and over time.

## 2 Methods

**FIGURE 1:**
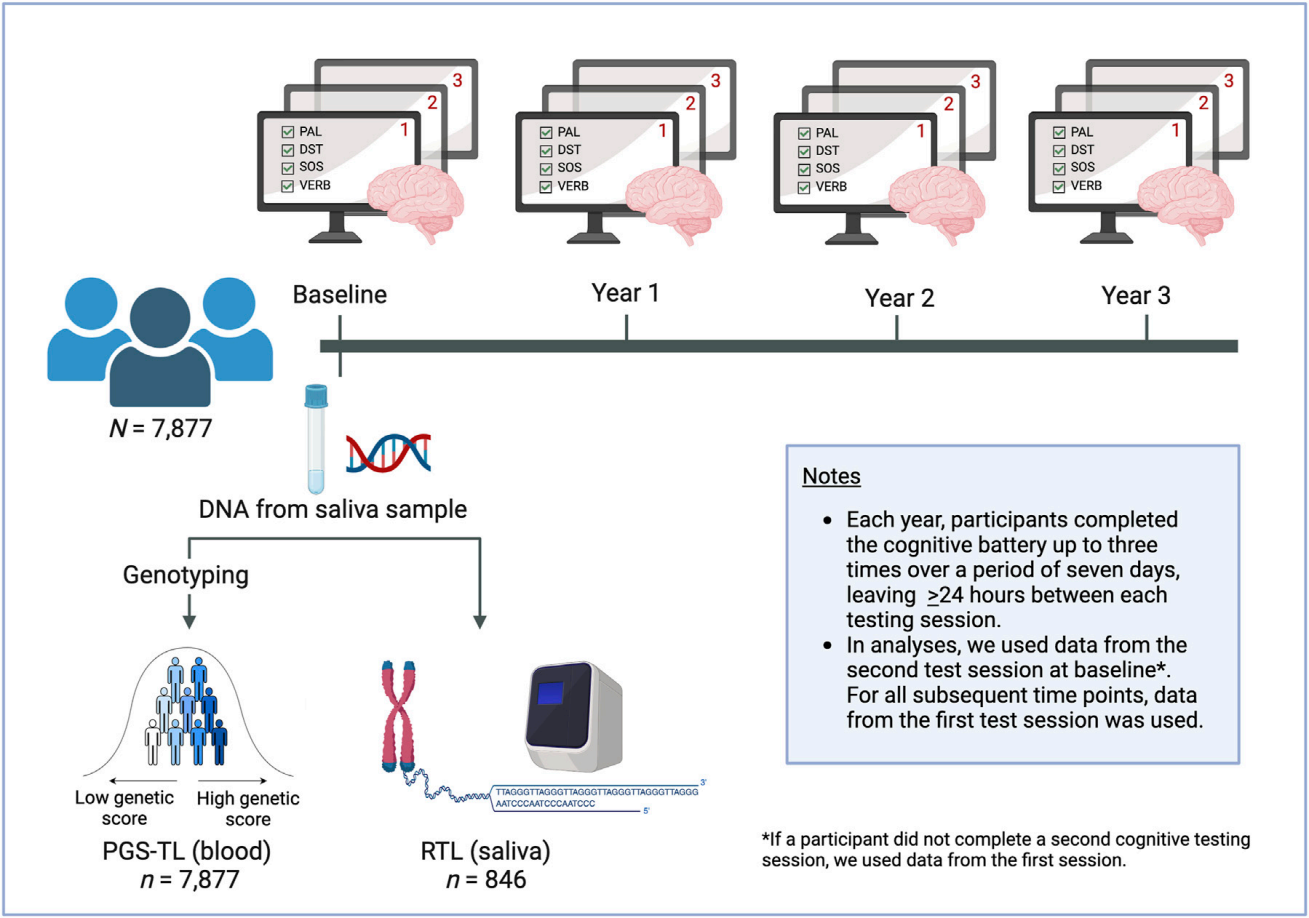
Study overview. Participants provided a saliva sample at baseline. The DNA was extracted and used to genotype participants, which enabled us to calculate polygenic scores for (leukocyte) telomere length (PGS-TL). We also used the DNA samples to quantify (saliva) relative telomere length (RTL). Participants completed the battery of four cognitive tests annually, optionally repeating the battery up to three times at each time point. We used latent growth models to analyse the data. Separate models were run with PGS-TL and RTL as predictors for each of the cognitive tests. We used data from the second test session at baseline (where possible), as the first was considered a practice session. At subsequent time points, data from the first test session was used. This figure was created with www.biorender.com/.

Figure 1 provides an overview of the study design.

### 2.1 Participants

All participants were from the online Platform for Research Online to investigate Genetics and Cognition in Ageing study (PROTECT; http://www.protectstudy.org.uk/). PROTECT is a longitudinal UK-based online participant registry that aims to understand the impact of lifestyle, medical and genetic risk factors on cognitive health and dementia risk in older adults. Inclusion criteria for enrolling in PROTECT are 1) ≥50 years old; 2) no diagnosis of dementia; and 3) access to a computer and the internet. Volunteers were prospectively recruited from November 2015 through both local and national publicity. Data collection was ongoing at the time of this study; therefore, a data freeze was implemented in October 2019 with data extracted for analyses up to this date. In the current study (*N* = 7,877), we used data from a subset of PROTECT study participants who 1) had complete baseline data for the cognitive and covariate variables (missing cognitive data at later time points was permitted), 2) had provided a saliva sample for genotyping and had genotype data available, and 3) were identified as individuals of European ancestry based on genetic principal components (PCs). These participants were generally comparable to the overall PROTECT study cohort in terms of participant characteristics, such as age, sex and education level (Supplementary Table S1). Participant characteristics of our full sample (*N* = 7,877) and the subsample with relative telomere length (RTL) data (*n* = 846) are shown in Supplementary Table S2. Participant characteristics split by median age for the full sample with polygenic scores for telomere length (PGS-TL) data and the subsample with RTL data are summarised in Supplementary Tables S3, S4, respectively.

Ethical approval was granted through the London Bridge National Research Ethics Committee (reference: 13/LO/1578) and informed consent obtained for all participants. The authors assert that all procedures contributing to this work comply with the ethical standards of the relevant national and institutional committees on human experimentation and with the Declaration of Helsinki 1975, as revised in 2008 (World Medical Association, 2013).

### 2.2 Cognitive assessment

Cognitive performance was assessed annually using an online cognitive test battery. We analysed participants’ data on four cognitive tests included in the PROTECT battery. A description of the four tests is provided in Table 1. Briefly, the tests included the paired associate learning (PAL) task of visual-spatial working memory and learning; digit span test (DST) of working memory; self-ordered search (SOS) of executive function and spatial memory; and verbal reasoning (VERB) task. At each annual testing, participants were instructed to complete the test battery up to three times over a period of 7 days, leaving at least 24 h between each cognitive testing session (only the first test session was mandatory). The tasks have parallel forms to ensure that repeated stimuli are not given to participants at each test session. The first cognitive test session at baseline was considered a practice session. In our analyses, we used data from the second testing session at baseline, where available, otherwise, we used data from the first testing session to maximise sample size and statistical power. For all subsequent time points, data from the first test session were used (see, Figure 1).

**TABLE 1:** Summary of the four cognitive tests included in the PROTECT battery.

Test	Description	Cognitive domain
Paired associates learning (PAL)	Boxes on screen are “opened” in a randomised order. One or more of the boxes contain a shape. Each shape is then presented in the middle of the screen and participants must identify which box the shape had been located in. If the participant makes an error, the boxes are opened in sequence again to remind the participant of the locations of the shape. The number of shapes per trial increases throughout the task making it increasingly difficult. Participants are given three attempts to successfully complete each level. The outcome measure was the average number of correct object-place associations (“paired associates”) in trials that were successfully completed. This task measures visual-spatial working memory and learning	Visual-spatial working memory and learning
Digit span test (DST)	A sequence of numbers appears on the screen (one at a time). At the sound of the beep, users click the numbers in the same order. Each successful trial is followed by a new sequence that is one digit longer than the last and each unsuccessful trial is followed by a new sequence that is one digit shorter than the last. This task measures working memory	Working memory
Self-ordered search (SOS)	A series of boxes are present on the screen; one of the boxes will contain a token. The participant selects each box until they locate the token. The token is then placed in another box and again the participant must locate it. Participants are informed that the diamond will never be in the same box twice. Higher scores are achieved through efficient location of the diamond. This task measures working memory	Executive function, spatial working memory
Verbal reasoning (VERB)	A statement appears at the top of the screen, and two objects underneath. The patient’s task is to reason about the relationships among the objects and determine if the statement is true or false. Responding quickly and accurately is required for high scores. There is no set upper or lower limit as the participants can attempt as many trials as they can manage within a specific timeframe. The outcome measure was the total number of trials answered correctly in 90 s, minus the number answered incorrectly. Higher scores indicate better performance. This task assesses verbal reasoning	Verbal reasoning

*Note*: Participants are asked to complete the PROTECT cognitive tests up to 3 times over 7 days leaving 24 h between in each at approximately yearly intervals.

### 2.3 Covariates

Following enrolment into the study, participants completed a series of online baseline questionnaires regarding demographic, lifestyle, and medical information. For the current analysis, the following demographic data were included as covariates: age, sex, education level (school until 16, school until 18, vocational qualification, undergraduate degree, post-graduate degree, and doctoral degree) and employment status (full-time, part-time, self-employed, retired, and unemployed) (dummy coded). Additionally, we included six genetic PCs, defined by the PROTECT study, as this number was determined to sufficiently adjust for population structure using scatter plots of the PCs incrementally plotted against each other until there was no clear pattern in the data (Patterson et al., 2006; Price et al., 2006). The selection of covariates was informed by previous genetic research in the PROTECT sample (i.e., Creese et al., 2021).

### 2.4 PROTECT genetic data

Saliva samples were collected by post and DNA was extracted by the National Institute for Health Research South London and the Maudsley National Health Service Biomedical Research Centre. Genotyping was completed using the Illumina Global Screening Array with custom content.

The total number of participants in the combined genotyped data for the whole PROTECT study was 9,146. Genotype quality control (QC) was performed on all 9,146 individuals, as described in Creese et al. (2021). The QC involved iterative filtering for call rate at 98% completeness (for individuals and SNPs) and then removing participants that were either related, not of European ancestry, of mismatched sex, outliers in the PC calculation or detected to have excess heterozygosity. This resulted in the exclusion of 84 samples due to incompleteness and the removal of a further 794 individuals following exclusions. Thus, a sample size of 8,268 participants remained.

Genotypes were imputed to 1,000 Genomes European reference panel using the Michigan imputation server and genotype phasing using Eagle (Loh et al., 2016). Variants were restricted to SNPs only, with a MAF > 0.001. An absolute cut-off of 0.7 was applied to the imputation quality of variants (Rsq as reported by the Michigan imputation server). The number of variants remaining after quality control was 9,415,055.

Further genotype QC was performed within the sample of 8,268 individuals of European ancestry, following all exclusions. The following SNP exclusions were applied, minor allele frequency (MAF) of <1% and those not in Hardy-Weinberg Equilibrium (*p*-value < .00001).

### 2.5 Polygenic scores for telomere length

We computed polygenic scores for telomere length (PGS-TL), using genome-wide summary statistics (clumped using 250 kb windows and *r^2^* > 0.1; MHC region included) from the largest GWAS of leukocyte telomere length to date (*N* = 464, 716 participants aged 40–69 years from the United Kingdom Biobank; Codd et al., 2021). We applied a *p*-value threshold for PGS-TL calculation (P_T_ = 3.9 × 10^−5^) equivalent to an FDR threshold of <1%, highlighted by Codd et al. (2021) to explain up to 5.64% of the variance in telomere length. The PGS-TL was calculated with PRSice-2 version 2.3.3 software (Choi and O’Reilly, 2019).

### 2.6 Measurement of telomere length

We generated relative telomere length (RTL) data for a subset of 846 participants. Relative telomere length was quantified using DNA samples and a modified version of the quantitative Polymerase Chain Reaction protocol described by Cawthon (Cawthon, 2009), as used by our lab previously (Coutts et al., 2019; Palmos et al., 2018; Palmos et al., 2020; Powell et al., 2018; Vincent et al., 2017); see Supplementary Methods S1 for further details.

Initially, we selected 908 participants from the overall study sample (*N* = 7,877) who met the following additional criteria: 1) complete cognitive data across time points (i.e., no missing cognitive data) 2) DNA was available from a saliva sample collected within 6 months of their baseline cognitive data collection. 54 samples (6%) did not survive our quality control (QC) criteria. Valid RTL measurements were therefore obtained for 854 participants.

We log-transformed the RTL data, as it was not normally distributed. We further adjusted for the day of run as this yielded significant batch effects (see Supplementary Table S5) and *Z*-standardised the data. Outliers were defined as residuals +/− 3.29 SDs from the mean, after fitting a linear regression model with age, sex, education level, employment status (dummy coded) and six genetic PCs (as described in Covariates). Based on this criteria, 8 samples were identified as outliers and were removed, leaving 846 valid telomere measurements for downstream analyses.

### 2.7 Statistical analyses

All statistical analyses and data cleaning were performed using RStudio version 1.3.1093 and R version 4.0.3. The PGS-TL and RTL data were *Z*-standardized (*M* = 0, *SD* = 1) before analysis. To examine relationships with RTL, we performed a multiple linear regression with RTL regressed on PGS-TL, age, sex, education level, employment status (dummy coded), and the six genetic PCs (see Covariates).

To examine cognitive performance over time, we used the R package lavaan (Rosseel, 2012) to construct latent growth models (informed by Berlin et al., 2014), with a Yuan-Bentler correction for non-normality (“estimator” = “mlr”), and full information maximum likelihood in models with missing data (“missing” = “fiml”), under the assumption that this missingness was at random (Arbuckle, 1996). As a sensitivity analysis, we repeated the latent growth modelling with complete cases (i.e., participants with no missing data across time points) for models that had included participants with missing data (see Sensitivity analyses). In the latent growth models, latent factors for the intercept (initial performance) and slope (change in performance over time) were estimated. We first fitted unconditional latent growth models (i.e., models in which the intercept and slope factors are not predicted by (conditioned on) predictors or covariates) for each of the four cognitive outcomes to determine the pattern of growth in the data. We tested no-growth (intercept-only), linear slope and quadratic slope models, using model fit statistics to determine which best described the data.

Next, conditional latent growth models were used to explore associations between measured (RTL) and genetically predicted telomere length (PGS-TL) and initial cognitive level (i.e., latent intercept) and trajectories over time (i.e., latent slope factors). Separate models were used for measured TL and PGS-TL and each cognitive outcome. The first six genetic PCs to adjust for population structure (Patterson et al., 2006; Price et al., 2006), age, sex, education level (school until 16, school until 18, vocational qualification, undergraduate degree, post-graduate degree, and doctoral degree), and employment status at baseline were included as time-invariant covariates.

For all latent growth models, the goodness-of-fit of the models was evaluated using several model fit statistics. Excellent models generally have the following values: CFI ≥.95, RMSEA < .05, and SRMR < .05 (Hu and Bentler, 1999). The chi-square statistic (χ^2^) is reported with degrees of freedom (*df*) but is not used as a measure of fitness, given its oversensitivity to large sample sizes.

Bonferroni correction for multiple testing was applied (i.e., *p* < .013; α = 0.05/4, to reflect the number of cognitive outcome variables) when assessing the relationships between telomere length (PGS-TL and RTL) and intercept and slope factor(s).

### 2.8 Sensitivity analyses

Firstly, to assess whether PGS-TL and RTL differentially predict cognition in middle and older age adults we repeated our analyses, using a median split based on age. A median split was used so that the sample size and power of each group were approximately equal. For more information on these analyses and how they were performed see Supplementary Methods S2.

Secondly, we repeated the latent growth modelling using only complete cases—participants with no missing data across time points—for models with PGS-TL included as a predictor. This step was taken to assess whether attrition and the inclusion of participants with missing data in the original models might have biased the parameter estimates, as these models were initially computed using full information maximum likelihood to handle missing data. For models using RTL as a predictor, which were already conducted with complete cases only, no additional sensitivity analyses were performed.

## 3 Results

Descriptive statistics for the raw cognitive data for the full sample with PGS-TL data (*N* = 7,877) and subsample with RTL data (*n* = 846) are shown in Supplementary Table S6, with each stratified by median age in Supplementary Tables S7, S8.

There was up to 36.35% missingness on the cognitive outcomes at the final time point. The amount of missing data on each cognitive outcome at each time point is summarised in Supplementary Table S9. Participants with complete data at all four time points (*n* = 4,722) performed significantly better at baseline (without correction for multiple testing) on three out of the four cognitive tests than participants with at least some missing data (*n* = 3,155); these participants also significantly differed on several covariates (see, Supplementary Table S10).

### 3.1 Predictors of relative telomere length (RTL)

Table 2 summarises the multiple regression results with RTL regressed on PGS-TL, age, sex, education level, employment status (dummy coded), and the six genetic PCs. Significant predictors of RTL included the PGS-TL (*β* = .07, *p* = .032), retirement status (*β* = .37, *p* = 001) and two of the genetic PCs (*β*s = .09 and .07, *ps* < .046). Age (*p* = .051), sex (*p* = .131) and education (*p* = .914) did not significantly predict RTL.

**TABLE 2:** Summary of multiple regression results predicting relative telomere length [95% confidence intervals] (*N* = 846).

Predictors	*B*	*SE B*	β	*sr* ^*2*^	*p*
(Intercept)	−1.07 [−1.99, −0.16]	0.46	0.25 [0.06, 0.43]	-	.**021**
PGS-TL	338.74 [28.83, 648.65]	157.89	0.07 [0.01, 0.14]	0.01	.**032**
pc1	−2.64 [−8.90, 3.61]	3.19	−0.03 [−0.10, 0.04]	<0.01	.408
pc2	−4.11 [−10.16, 1.95]	3.08	−0.05 [−0.11, 0.02]	<0.01	.183
pc3	1.31 [−4.46, 7.09]	2.94	0.02 [−0.05, 0.08]	<0.01	.655
pc4	0.37 [−5.07, 5.81]	2.77	0.00 [-0.06, 0.07]	<0.01	.894
pc5	7.19 [1.47, 12.90]	2.91	0.09 [0.02, 0.15]	0.01	**.014**
pc6	6.62 [0.11, 13.12]	3.31	0.07 [0.00, 0.14]	<0.01	**.046**
Age	0.01 [−0.00, 0.03]	0.01	0.09 [−0.00, 0.17]	<0.01	.051
Sex	0.12 [−0.04, 0.29]	0.08	0.05 [−0.02, 0.12]	<0.01	.131
Education	0.00 [−0.05, 0.05]	0.03	0.00 [−0.07, 0.06]	<0.01	.914
*Employment (fulltime)*					
Employed (part-time)	−0.17 [−0.40, 0.06]	0.12	−0.17 [−0.40, 0.06]	<0.01	.159
Self-employed	−0.14 [−0.41, 0.13]	0.14	−0.14 [−0.41, 0.13]	0.01	.295
Retired	−0.37 [−0.60, −0.14]	0.12	−0.37 [−0.60, −0.14]	<0.01	**.001**
Unemployed	−0.29 [−0.72, 0.15]	0.22	−0.29 [−0.72, 0.15]	<0.01	.198
Overall model fit	*F* (23, 822) = 12.17, *p* < .001, *R* ^*2*^ */R* ^*2*^ *adjusted* = 0.034/0.017				

*Note*. PGS-TL, polygenic score for telomere length; pc = genetic principal components. Relative telomere length (log-transformed, adjusted for batch) and PGS-TL, were *Z*-standardised in the whole sample to a mean of 0 and SD, of 1. Outliers of +/− 3.29 SDs, from the mean (adjusting for covariates) were removed from the relative telomere length variable (see, Methods). Employment was dummy-coded, with full-time employment as the reference category in brackets.

### 3.2 Prediction of cognitive trajectories: unconditional models

First, we fitted unconditional models (i.e., without the intercept and slope factors conditioned on predictors or covariates) for each of the four cognitive outcomes to determine the pattern of growth in the data. Across the full sample and all subsamples (i.e., split by median age and/or those with RTL data), unconditional model fit statistics suggested that linear models provided an acceptable fit to the data for the paired associat learning (PAL), digit span test (DST), and self-ordered search (SOS) tasks, whereas for the verbal reasoning (VERB) task, a quadratic model provided acceptable fit (see, Supplementary Tables S11–S22 for the fit indices and parameter estimates for the unconditional models).

Next, we fitted conditional models to explore whether PGS-TL or RTL predicted baseline cognitive level (i.e., latent intercept) and/or change over time (i.e., latent slope factors). Although these models were adjusted for covariates, we do not report their corresponding parameter estimates in the main text for simplicity. Parameter estimates with 95% confidence intervals are reported in full, including those related to covariates, for each model within the Supplementary Tables S23–S28.

### 3.3 Prediction of cognitive trajectories: whole sample with PGS-TL

PGS-TL did not predict baseline cognitive performance (captured by the intercept factor; *ps* ≥ .238) or change over time (captured by the slope factor(s); *ps* ≥ .080) on any of the cognitive tests, after adjusting for covariates. Conditional model fit statistics for the full sample can be found in Supplementary Table S29 and parameter estimates with confidence intervals are summarised in Supplementary Table S23.

### 3.4 Prediction of cognitive trajectories: subsample with RTL data

RTL did not predict baseline cognitive performance (*ps* ≥ .154) or change over time (*ps* ≥ .276) on any of the cognitive tests, after adjusting for covariates. Conditional model fit statistics for the RTL subsample can be found in Supplementary Table S29 and parameter estimates with confidence intervals can be found in Supplementary Table S24.

### 3.5 Sensitivity analyses: analyses split by median age

To assess whether PGS-TL and RTL differentially predict cognition in middle and older age adults we repeated our analyses, using a median split based on age. In the full sample with PGS-TL data (*N* = 7,877) the median age was 62.24 years, in the subsample with RTL data (*n* = 846) it was 62.77 years.

#### 3.5.1 Whole sample with PGS-TL: median age split

**FIGURE 2:**
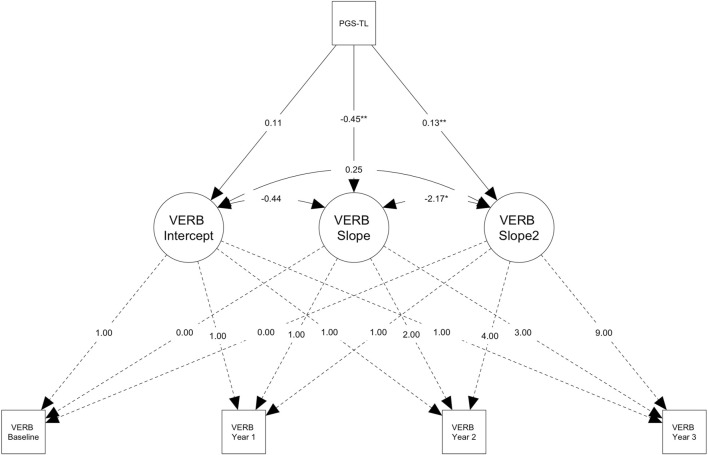
Path diagram for the conditional latent growth model showing genetically predicted telomere length (PGS-TL) predicts change in verbal reasoning (VERB) performance in older participants (> ∼62.24 years; n = 3,939). Path diagram for the conditional latent growth model showing genetically predicted telomere length (PGS-TL) predicts the slope factor (i.e., increase in performance over time, M = 3.23) and the quadratic slope factor (i.e., acceleration in the rate of change, M = 0.21) for the verbal reasoning test in the subsample of older participants (> ∼62.24 years; n = 3,939). Rectangles denote measured variables. Circles denote latent factors (i.e., intercept = baseline performance, slope = linear change, slope^2^ = acceleration or deceleration in the rate of change). Though effect sizes were small, individuals with higher PGS-TL initially experienced slower improvements on the verbal reasoning task, followed by a more rapid increase in performance over time. PGS-TL did not significantly predict baseline performance. Note that the model was adjusted for several covariates (see, Methods) and additional parameters were also estimated (e.g., intercept and slope means), but these are not shown on the diagram for simplicity. The PGS-TL was Z-standardised to a mean of 0 and standard deviation of 1. Residual terms were freely estimated (heteroscedastic) and full information maximum likelihood for missing data with robust maximum likelihood estimation was used (see Methods). *p < .05; **p < .01.

**FIGURE 3:**
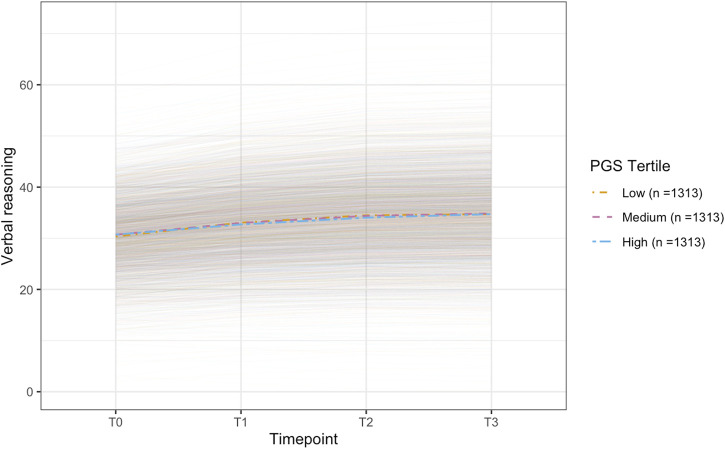
Plot of the conditional latent growth model predicted (model-implied) trajectories for older participants (> ∼62.24 years; n = 3,939) on the verbal reasoning task. The model included polygenic scores for telomere length (PGS-TL) as a predictor and was adjusted for several covariates (see, Methods). Though effect sizes were small, individuals with higher PGS-TL initially experienced slower improvements on the verbal reasoning task, followed by a more rapid increase in performance over time. PGS-TL did not significantly predict baseline performance. The PGS-TL was Z-standardised to a mean of 0 and standard deviation of 1. To show how trajectories differ across different levels of the PGS-TL, we plot the mean trajectory for each PGS-TL tertile to aid interpretation (though PGS-TL was modelled as a continuous variable).

In older adults (*n* = 3,939), PGS-TL was a significant predictor of change over time (i.e., slope factor, *M*_*slope*_ = 3.23, *B* = −0.45, *p* = .001; slope^2^ factor, *M*_*slope*_^*2*^ = 0.21, *B* = 0.13, *p* = .002), but not baseline cognitive performance (*p* = .438) on the VERB task (Figures 2, 3). On the remaining cognitive tasks PGS-TL did not significantly predict older adults’ baseline cognitive performance (*ps* ≥ .508) or change over time (*ps* ≥ .090) (Table 3). In middle-aged adults (*n* = 3,938), PGS-TL did not significantly predict baseline cognitive performance (*ps* ≥ .352) or change over time (*ps* ≥ .192) on any of the cognitive tests (Table 3). All models were adjusted for covariates. Conditional model fit statistics for models split by median age can be found in Supplementary Table S30.

**TABLE 3:** Parameter estimates [95% confidence intervals] for the latent growth models conditional on polygenic scores for telomere length for each of the cognitive outcomes in the whole sample split by median age (∼62.24 years) at baseline.

	Middle-aged (*n* = 3,938)				Older (*n* = 3,939)			
Parameter	Paired associate learning	Digit span test	Self-ordered search	Verbal reasoning	Paired associate learning	Digit span test	Self-ordered search	Verbal reasoning
*Means*								
Intercept	5.45 [4.97, 5.93]	7.89 [7.05, 8.73]	11.61 [10.29, 12.93]	42.40 [36.97, 47.83]	6.32 [5.87, 6.78]	10.06 [9.26, 10.86]	13.35 [12.16, 14.55]	54.72 [49.77, 59.67]
Slope	0.01 [−0.23, 0.24]	0.19 [−0.11, 0.50]	0.32 [−0.29, 0.94]	0.52 [−4.95, 6.00]	0.09 [−0.12, 0.30]	0.11 [−0.20, 0.42]	−0.28 [−0.86, 0.29]	3.23 [−1.13, 7.60]
Slope^2^	-	-	-	0.04 [−1.70, 1.78]	-	-	-	0.21 [−1.15, 1.58]
*Variances*								
Intercept	0.27 [0.21, 0.34]	1.32 [1.14, 1.51]	2.28 [1.92, 2.65]	64.92 [56.93, 72.92]	0.19 [0.15, 0.24]	1.70 [1.39, 2.01]	1.85 [1.52, 2.18]	55.55 [48.28, 62.81]
Slope	0.02 [0.01, 0.03]	0.00^a^	0.15 [0.06, 0.24]	11.43 [2.47, 20.39]	0.00 [−0.01, 0.01]	0.06 [0.02, 0.10]	0.08 [−0.01, 0.17]	9.67 [1.22, 18.12]
Slope^2^	-	-	-	1.03 [0.40, 1.66]	-	-	-	0.55 [−0.05, 1.14]
*Covariances*								
Intercept-Slope	−0.03 [−0.05, −0.00]	−0.01 [−0.04, 0.02]	−0.05 [−0.20, 0.10]	1.84 [−6.74, 10.42]	0.01 [−0.01, 0.03]	−0.12 [−0.20, −0.03]	0.02 [−0.13, 0.16]	−0.44 [−8.42, 7.55]
Intercept-Slope^2^	-	-	-	−0.14 [−2.28, 1.99]	-	-	-	0.25 [−1.74, 2.24]
Slope-Slope^2^	-	-	-	−3.11 [−5.21, −1.00]	-	-	-	−2.17 [−4.17, −0.17]
*Residual variances*								
Baseline	0.59 [0.53, 0.66]	1.26 [1.06, 1.46]	3.40 [2.96, 3.84]	25.21 [17.55, 32.88]	0.70 [0.62, 0.77]	1.38 [1.13, 1.63]	3.96 [3.53, 4.39]	22.79 [15.78, 29.81]
Year 1	0.61 [0.56, 0.65]	0.88 [0.78, 0.97]	5.76 [5.22, 6.31]	22.79 [19.91, 25.66]	0.68 [0.62, 0.73]	1.33 [1.09, 1.56]	5.10 [4.65, 5.56]	20.89 [18.32, 23.46]
Year 2	0.57 [0.52, 0.61]	0.92 [0.80, 1.04]	4.34 [3.86, 4.83]	22.85 [19.88, 25.82]	0.59 [0.55, 0.64]	1.17 [0.98, 1.36]	4.27 [3.82, 4.71]	19.89 [17.19, 22.59]
Year 3	0.55 [0.49, 0.61]	0.87 [0.71, 1.02]	3.33 [2.76, 3.90]	16.69 [7.53, 25.86]	0.56 [0.50, 0.63]	0.86 [0.67, 1.05]	4.19 [3.63, 4.74]	18.45 [10.38, 26.53]
*Predictor estimates*								
Intercept ∼ PGS-TL	−0.01 [−0.04, 0.01]	−0.02 [−0.07, 0.03]	0.03 [−0.04, 0.10]	0.13 [−0.17, 0.42]	0.01 [−0.02, 0.03]	−0.01 [−0.06, 0.04]	0.02 [−0.05, 0.09]	0.11 [−0.17, 0.39]
Slope ∼ PGS-TL	0.00 [−0.01, 0.02]	0.01 [−0.01, 0.03]	−0.02 [−0.05, 0.02]	0.19 [−0.10, 0.48]	−0.00 [−0.02, 0.01]	−0.01 [-0.02, 0.01]	−0.03 [−0.06, 0.00]	−0.45 [−0.72, −0.18]
Slope^2^ ∼ PGS-TL	-	-	-	−0.05 [−0.14, 0.04]	-	-	-	0.13 [0.05, 0.22]

*Note*. PGS-TL, polygenic score for telomere length (leukocyte). PGS-TL, was *Z*-standardised to a mean of 0 and SD, of 1. The intercept factor captures the baseline level of cognitive performance, the slope factor reflects the rate of change (i.e., increase/decrease), and the inclusion of a quadratic slope factor indicates the change overtime is non-linear and captures either the acceleration or deceleration in the rate of change in the outcome variable. Covariances measure the degree to which two variables change together (e.g., a negative intercept-slope covariance indicates that individuals with higher baseline cognitive performance tend to have less steep increases over time, assuming a positive slope factor). Residual variances represent the variability in the observed data that is not explained by the latent growth model. Finally, parameter estimates reflect the influence of predictor variables on the latent growth factors (i.e., intercept and slopes). All models used full information maximum likelihood for missing data with robust maximum likelihood estimation and were adjusted for several covariates, not included here for simplicity (see, Supplementary Tables S25, S26 for full results for the middle- and older-age adults, respectively).

^a^ The slope variance was fixed to 0.0001 (i.e., estimated as a fixed effect; see Supplementary Methods S2), hence no confidence intervals are provided.

#### 3.5.2 Subsample with RTL data: median age split

**FIGURE 4:**
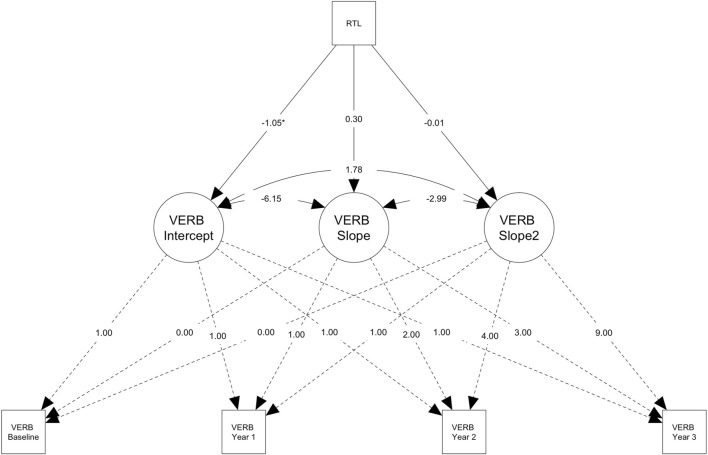
Plot of the conditional latent growth model predicted (model-implied) trajectories for older participants (> ∼62.77 years; n = 423) on the verbal reasoning task. Path diagram for the conditional latent growth model showing that longer relative telomere length (RTL) predicts worse performance on the verbal reasoning test (VERB) at baseline (i.e., intercept factor, M = 47.58), but not predict change over time, in the subsample of older participants (>∼62.77 years; n = 423). Rectangles denote measured variables. Circles denote latent factors (i.e., intercept = baseline performance, slope = linear change, slope^2^ = acceleration or deceleration in the rate of change). Note that the model was adjusted for several covariates (see, Methods) and additional parameters were also estimated (e.g., intercept and slope means), but these are not shown on the diagram for simplicity. The RTL was Z-standardised to a mean of 0 and standard deviation of 1 within the sample with RTL data (n = 846). Residual terms were freely estimated (heteroscedastic) and full information maximum likelihood for missing data with robust maximum likelihood estimation was used (see Methods) *p < .05.

**FIGURE 5:**
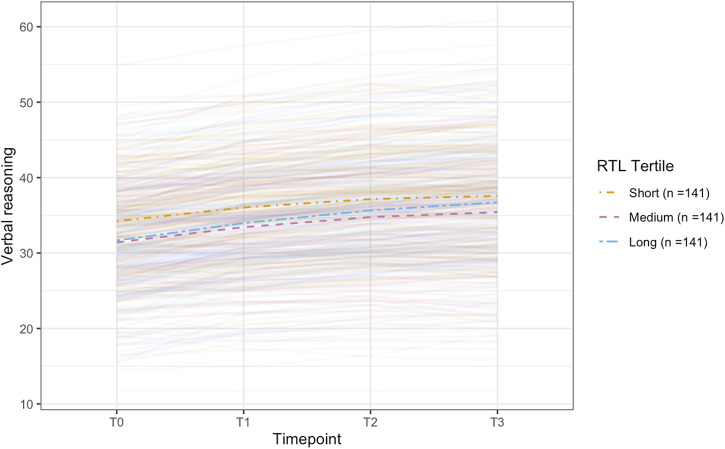
Plot of the conditional latent growth model predicted (model-implied) trajectories for older participants (> ∼62.77 years; n = 423) on the verbal reasoning task. The model included relative telomere length (RTL) as a predictor and was adjusted for several covariates (see, Methods). Longer RTL predicted worse performance on the verbal reasoning task at baseline but did not significantly predict change over time. The RTL was Z-standardised to a mean of 0 and standard deviation of 1. To show how trajectories differ across different levels of the RTL, we plot the mean trajectory for each RTL tertile to aid interpretation (though RTL was modelled as a continuous variable).

In older adults (*n* = 423), RTL significantly predicted the intercept (*M*_*interce*pt_ = 47.58, *B* = −1.05, *p* = .011) in older adults on the VERB task, whereby longer telomere length was associated with worse baseline performance; RTL did not predict either of the slope factors (*ps* ≥ .440) (see, Figures 4, 5). Further, RTL did not significantly predict older adults’ baseline cognitive performance (*ps* ≥ .265) or change over time (*ps* ≥ .391) on the PAL, DST or SOS tests (Table 4). In middle-aged adults (*n* = 423), RTL did not significantly predict baseline cognitive performance (*ps* ≥ .366) or change over time (*ps* ≥ .139) on any of the cognitive tests (Table 4). All models were adjusted for covariates. Conditional model fit statistics for models split by median age can be found in Supplementary Table S30.

**TABLE 4:** Parameter estimates [95% confidence intervals] for the latent growth models conditional on relative telomere length for each of the cognitive outcomes split by median age (∼62.77 years) at baseline.

	Middle-aged (*n* = 423)				Older (*n* = 423)			
Parameter	Paired associate learning	Digit span test	Self-ordered search	Verbal reasoning	Paired associate learning	Digit span test	Self-ordered search	Verbal reasoning
*Means*								
Intercept	6.54 [5.26, 7.83]	8.73 [6.55, 10.90]	10.19 [6.53, 13.85]	34.20 [18.86, 49.53]	7.50 [6.22, 8.79]	9.01 [6.45, 11.57]	14.07 [9.90, 18.25]	47.58 [33.02, 62.14]
Slope	−0.01 [−0.62, 0.60]	−0.22 [−0.94, 0.49]	1.38 [−0.09, 2.85]	10.01 [−2.15, 22.16]	−0.02 [−0.59, 0.55]	0.41 [−0.49, 1.31]	0.59 [−1.13, 2.31]	6.19 [−7.21, 19.60]
Slope^2^	-	-	-	−2.62 [−6.47, 1.23]	-	-	-	−0.64 [−4.76, 3.49]
*Variances*								
Intercept	0.12 [0.01, 0.23]	1.22 [0.98, 1.46]	1.11 [0.39, 1.83]	52.09 [31.02, 73.16]	0.24 [0.13, 0.34]	1.18 [0.87, 1.50]	1.93 [1.04, 2.83]	50.72 [32.33, 69.11]
Slope	0.01 [−0.02, 0.03]	0.01 [−0.02, 0.05]	0.04 [−0.16, 0.24]	6.90 [−15.63, 29.43]	0.02 [−0.00, 0.04]	0.04 [−0.01, 0.09]	0.07 [−0.14, 0.28]	12.04 [−9.60, 33.67]
Slope^2^	-	-	-	1.60 [0.18, 3.02]	-	-	-	0.77 [−0.66, 2.20]
*Covariances*								
Intercept-Slope	0.01 [−0.04, 0.05]	−0.03 [−0.10, 0.03]	0.18 [−0.12, 0.48]	5.59 [−16.13, 27.30]	−0.03 [−0.07, 0.01]	−0.04 [−0.16, 0.08]	−0.12 [−0.48, 0.25]	−6.15 [−25.60, 13.31]
Intercept-Slope^2^	-	-	-	−0.60 [−6.09, 4.89]	-	-	-	1.78 [−3.05, 6.61]
Slope-Slope^2^	-	-	-	−3.24 [−8.46, 1.98]	-	-	-	−2.99 [−8.05, 2.08]
*Residual variances*								
Baseline	0.61 [0.46, 0.76]	0.73 [0.48, 0.98]	3.00 [1.92, 4.07]	27.75 [8.86, 46.64]	0.55 [0.42, 0.69]	1.44 [0.93, 1.95]	3.65 [2.45, 4.85]	19.87 [2.32, 37.43]
Year 1	0.57 [0.47, 0.67]	0.74 [0.61, 0.87]	5.26 [3.87, 6.66]	17.97 [11.78, 24.15]	0.58 [0.49, 0.66]	0.82 [0.65, 0.99]	4.17 [3.05, 5.29]	21.56 [15.84, 27.28]
Year 2	0.57 [0.48, 0.66]	0.76 [0.60, 0.91]	3.97 [2.83, 5.10]	24.18 [15.51, 32.85]	0.50 [0.43, 0.58]	0.65 [0.52, 0.79]	4.12 [2.96, 5.29]	15.08 [8.79, 21.37]
Year 3	0.52 [0.39, 0.66]	0.83 [0.55, 1.11]	3.28 [2.08, 4.48]	8.86 [−9.93, 27.65]	0.44 [0.34, 0.55]	0.65 [0.43, 0.87]	5.26 [3.75, 6.77]	21.95 [2.13, 41.78]
*Predictor estimates*								
Intercept ∼ RTL	−0.02 [−0.09, 0.05]	0.06 [−0.07, 0.18]	−0.08 [−0.30, 0.15]	0.22 [−0.59, 1.03]	0.03 [−0.04, 0.11]	−0.08 [−0.22, 0.06]	0.10 [−0.11, 0.30]	−1.05 [−1.85, −0.24]
Slope ∼ RTL	0.01 [−0.02, 0.04]	−0.02 [−0.06, 0.02]	0.02 [−0.07, 0.11]	−0.64 [−1.48, 0.21]	−0.01 [−0.04, 0.03]	0.01 [−0.04, 0.07]	−0.04 [−0.12, 0.05]	0.30 [−0.46, 1.06]
Slope^2^ ∼ RTL	-	-	-	0.19 [−0.08, 0.46]	-	-	-	−0.01 [−0.24, 0.22]

*Note*. RTL, relative telomere length (from saliva, log-transformed, batch adjusted). RTL, was *Z*-standardised within the full subsample, and outliers were removed (+/− 3.29 SDs, from the mean, adjusting for covariates). The intercept factor captures the baseline level of cognitive performance, the slope factor reflects the rate of change (i.e., increase/decrease), and the inclusion of a quadratic slope factor indicates the change overtime is non-linear and captures either the acceleration or deceleration in the rate of change in the outcome variable. Covariances measure the degree to which two variables change together (e.g., a negative intercept-slope covariance indicates that individuals with higher baseline cognitive performance tend to have less steep increases over time, assuming a positive slope factor). Residual variances represent the variability in the observed data that is not explained by the latent growth model. Finally, parameter estimates reflect the influence of predictor variables on the latent growth factors (i.e., intercept and slopes). All models used full information maximum likelihood for missing data with robust maximum likelihood estimation and were adjusted for several covariates, not included here for simplicity (see, Supplementary Tables S27, S28 for full results for the middle- and older-age adults, respectively).

### 3.6 Sensitivity analyses: complete case analyses

We repeated the latent growth modelling for models that had included participants with missing data (i.e., those with PGS-TL as a predictor), using only complete cases only—participants with no missing data at any time point. These analyses yielded similar results to those that included participants with missing data, see Supplementary Tables S31–S33 for parameter estimates and Supplementary Table S34 for model fit statistics). The direction of effect and the significance of PGS-TL as a predictor remained consistent for all outcomes, both in the full sample and within each age subgroup divided by median age. All models using RTL as a predictor were already performed in complete cases only, and so no sensitivity analyses were completed for these models.

## 4 Discussion

To our knowledge, this is the first study to examine the relationship between telomere length and longitudinal cognitive performance in normative ageing, using both measured (RTL) and genetically predicted (PGS-TL) telomere length. Our PGS-TL captured lifelong genetic predisposition to telomere length, likely more independently of environmental effects. Whereas our RTL encapsulated both environmental and genetic effects measured at a single point in time. Though our measures of telomere length were derived from different tissue types, we showed that our PGS-TL (blood) positively predicted RTL (saliva). However, we found that neither measure of telomere length predicted cognitive performance (baseline or change over time) in the whole sample overall, or in participants aged <62 years when stratifying by median age. Whereas, contrary to expectations, in older adults (≥ ∼62 years) longer RTL was associated with worse performance at baseline on the verbal reasoning test; though this result did not survive correction for multiple testing. Furthermore, higher PGS-TL (indicating a genetic predisposition to longer telomere length) was associated with smaller increases in performance on the verbal reasoning test across the first 2 years. However, the inverse was observed from year two to year three, whereby higher PGS-TL predicted better performance (i.e., larger increases). Nonetheless, the PGS-TL effect sizes were small and unlikely to be clinically meaningful.

Many models showed a positive mean slope factor, indicating a mean increase in cognitive performance over time. This was the case both in models accounting for missing data using full-information maximum likelihood and in those using complete cases. Therefore, outcome-related attrition alone does not explain these increases. Previous research indicates that cognitive performance often improves over repeated testing due to practice effects (Goldberg et al., 2015), even when different test versions are administered in successive sessions (Salthouse and Tucker-Drob, 2008). Less robust practice effects can signal current cognitive status and future decline, and are associated with neurodegeneration biomarkers (Jutten et al., 2020). We therefore interpret the results of this study in light of this.

Our results did not agree with previously published research, suggesting predictive utility of longer telomere length for better cognitive performance in middle- and older aged adults (e.g., Hägg et al., 2017). Though some other studies have reported non-significant associations between telomere length and cognitive performance, they have generally consisted of small sample sizes and are therefore limited by low statistical power (e.g., Sánchez-González et al., 2022). In our sample, telomere length appeared to have only some limited predictive biomarker properties that were specific to verbal reasoning in older adults. However, the significant effects of telomere length were generally in the opposite direction to what was hypothesised. Inverse associations between other risk factors and cognitive outcomes in older ages have previously been reported (e.g., PGS and measured C-reactive protein; Packer et al., 2023; Silverman et al., 2009). One explanation for this is that the association of the risk factor with cognition does not change within an individual with increased age, but rather there is a survivor bias in which the observed association in the population changes due to differential mortality and/or study participation (Anderson et al., 2024). This may be exacerbated in the current sample for which use of a computer and no dementia diagnosis was a requirement for enrolment to the study, which would have disproportionately affected the participation of older individuals. Additionally, participation bias is frequently observed in large-scale studies, often resulting in samples that are healthier and of higher socioeconomic status (e.g., Schoeler et al., 2023). This bias has been shown to reduce or even reverse effects seen in more representative samples (e.g., Alten et al., 2022). The current sample showed evidence of participation bias, such as being more highly educated than the general population of the same age (see Limitations).

We also observed that relative to full-time employment, being retired was associated with shorter RTL (after adjusting for covariates, such as age and education). Retirement represents a major life transition marked by psychological and financial stress, as well as lifestyle changes in physical activity, sleep patterns, social engagement, and dietary habits (Wanka, 2020). Given RTL represents a marker sensitive to the effects of a wide variety of environmental stress, it seems plausible such factors could explain this RTL association with retirement. Alternatively, individuals with accelerated biological aging (indicated by shorter telomeres) are more likely to experience poorer mental and physical health. This may increase their likelihood of retiring early and decrease their chances of remaining employed (Topa et al., 2018).

Overall, our results draw into question the utility of telomere length as an ageing biomarker that is capable of predicting cognitive performance and age-related change, in typically ageing individuals. Alternative indicators of biological ageing may better predict cognition in middle-older aged adults, such as epigenetic clocks (i.e., blood-based DNA methylation measures of ageing). Notably, the third generation of epigenetic clocks that capture the rate of ageing, such as the DunedinPACE (Dunedin Pace of Aging Calculated from the Epigenome; Belsky et al., 2022), has emerged as a promising tool for identifying individuals at risk for cognitive decline (Sugden et al., 2022).

### 4.1 Limitations

This study had several limitations. First, our measures of telomere length were derived from different tissue sources. Saliva is easier to collect but is arguably a less valid tissue source than blood, which has a clearer link to brain ageing (Pluvinage and Wyss-Coray, 2020). However, telomere length is generally positively correlated across different tissue types (Demanelis et al., 2020), and genetic factors for leukocyte telomere length (i.e., PGS-TL) predicted salivary telomere length in our study. Second, the PGS-TL may have been underpowered. While telomere length is highly heritable (i.e., ∼70% according to meta-analytic estimates, Broer et al. (2013); with SNP heritability estimates of 8.1%; Codd et al., 2021), the PGS-TL only explains up to ∼5.64% of the variance in leukocyte telomere length (Codd et al., 2021). Therefore, larger sample sizes and more powerful GWAS may be required to improve the predictive power of the PGS-TL and to address this “missing heritability”. Third, although we adjusted our analyses for several covariates, we cannot exclude the possibility that unmeasured confounding may have biased our results. For example, factors such as physical activity, which may influence both telomere length and cognitive function, could have biased our results. Fourth, limited variability in scores on the cognitive tests (potentially reflective of our homogenous sample) and relatively short follow-up period may have hindered our power to detect significant effects related to age-related cognitive decline. Additionally, associations between telomere length and cognitive performance may exist in cognitive domains not assessed by the four tests included in this study (e.g., reaction time). Finally, as in all research, we urge caution in extrapolating our findings beyond the current sample. For example, the study consisted of white UK adults only, and women and individuals of a higher education level were overrepresented in our study, relative to the general population (see, Office for National Statistics, http://www.ons.gov.uk). Research in more representative and diverse samples is greatly needed, especially given that telomere length may be differentially associated with cognitive function across sociodemographic groups (see, Leibel et al., 2019).

## 5 Conclusion

In summary, we found little evidence to suggest that telomere length (RTL and PGS-TL) predicts cognitive performance, in adults aged ≥50 years of European ancestry. Alternative indicators of biological ageing, such as the third generation of epigenetic clocks that capture the pace of ageing (e.g., DunedinPACE), may provide better utility in predicting individuals at risk for cognitive decline.

## Data Availability

This study was conducted using secondary data collected as part of the United Kingdom version of the PROTECT ongoing study. PROTECT data are available to investigators outside the PROTECT team after request and approval by the PROTECT Steering Committee. Requests to access these datasets should be directed to protect.data@exeter.ac.uk.
