# Supplementary material for: Telomere length and cognitive changes in 7,877 older UK adults of European ancestry
Source: Front Aging. 2024 Nov 1;5:1480326. doi: 10.3389/fragi.2024.1480326 (PMC11564160; doi:10.3389/fragi.2024.1480326)
Supplement: Supplementary file 1 [file DataSheet1.docx]

Supplementary Material

#

# Supplementary methods

## Supplementary methods 1: Telomere length quantification

To assess relative telomere length, we performed a modified version of a quantitative polymerase reaction (qPCR) protocol by Cawthon and colleagues (see Main Text). The protocol involves two separate qPCRs performed on separate 384-well plates with DNA samples pipetted into identical wells on each plate. In the first reaction, we assayed the telomere repeat region (TTAGGG). In the second reaction, we assayed a single copy gene, albumin, which we used as an internal control to correct for differences in DNA concentration between samples. The telomere/albumin ratio was used to calculate relative telomere length.

On each plate, one negative control consisting of RNase-free water was used to screen for any DNA contamination, and five positive controls consisting of independent leukocyte DNA samples were used to confirm a successful PCR. An eight-point dilution series using human leukocyte genomic DNA (0.47, 0.94, 1.88, 3.75, 7.5, 15, 30, and 60 ng) was used on each plate to allow for absolute quantification of each sample and to account for any differences in efficiency between the telomere and albumin reactions. All reactions were performed using three technical replicates. Each qPCR mix for the telomere reactions consisted of 10 μL of 2x qPCR Mastermix with SYBR Green (Primer Design, Southampton, United Kingdom), 5 μL or RNase free water, 12 ng of DNA, 1000 nM of telg, 5′-ACACTAAGGTTTGGGTTTGGGTTTGGGTTTGGGTTAGTGT-3′ and 800nM of telc, 5′-TGTTAGGTATCCCTATCCCTATCCCTATCCCTATCCCTAACA-3′. Four stages made up the thermocycling conditions as follows: Stage 1: 95°C for 15 min, Stage 2: 2 cycles for 15 s at 94°C and 49°C, Stage 3: 25 cycles at 94°C for 15 s, 10 s at 62°C, and 15 s at 73°C (data collection), Stage 4: dissociation curve (primer specificity detection).

The same reagents and quantities were used for the albumin reactions, apart from the albumin forward and reverse primers replaced the telomere primers. Quantities of the albumin forward and reverse primers were adjusted to 765 nM for the forward primer albu, 5′-CGGCGGCGGGCGGCGCGGGCTGGGCGGAAATGCTGCACAGAATCCTT-3′ and 930 nM for the reverse primer albd, 5′-GCCCGGCCCGCCGCGCCCGTCCCGCCGGAAAAGCATGGTCGCCTGTT-3′. The thermocycling conditions for the albumin reaction consisted of four stages: Stage 1: 95°C for 15 min, Stage 2: 2 cycles for 15 s at 94°C and 49°C, Stage 3: 33 cycles at 94°C for 15 s, 10 s at 62°C, and 15 s at 88°C (data collection), Stage 4: dissociation curve (primer specificity detection). Reactions were performed using the QuantStudio 5 Real-Time PCR System (Thermofisher Scientific).

Quality control included checking for primer amplification specificity using melting curves, ensuring high primer efficiencies (90-110%), and confirming an R^2^ >0.985 for all standard curves. Following this, single outliers were identified and removed if Ct values corresponding to technical triplicates generated a standard deviation (SD) of above 0.5. Ct values were related to absolute quantities as part of a standard curve to generate Cq values. The average (mean) Cq value was then generated across replicates, for each sample and positive control. Next, the average (mean) Cq of all positive controls was calculated; one positive control on one of the albumin plates did not survive quality control, therefore only four positive controls were used to calculate the mean. To reduce inter-plate variability, each sample average Cq was then divided by the average Cq of the positive controls (i.e., positive control normalised). To generate relative telomere length, the positive control normalised average Cq value pertaining to the telomere repeat sequence was divided by the positive control normalised average Cq relating to the single copy gene, albumin. When repeating a set of 80 samples using this method on separate days, it yielded an intraclass correlation coefficient (ICC) of 0.721, CI [0.606, 0.809], *p* = 1.63 x 10^-15^, indicating good reproducibility.

## Supplementary Methods 2: Lavaan warnings

When estimating the unconditional and conditional linear models fit to the DST data from the middle-aged participants, we received a lavaan warning message about a negative variance for the slope factor (suggesting almost zero variation in the slope across participants). We addressed this problem by fixing the slope variance to a small number (i.e., 0.0001), modelling it as a fixed effect rather than random in the DST models. This solution fixed the problem, and no additional warnings were generated.

When estimating several of the conditional models in the older participants with RTL data, we received a lavaan warning indicating the variance-covariance matrix of the estimated parameters (vcov) did not appear to be positive definite. This appeared to be caused by the dummy variable for unemployment (vs. full-time employment), as only a small number of individuals within this sample were unemployed (*n* = 2, i.e., 0.47%; see Supplementary Table 3). This error was resolved by collapsing the unemployed and retired dummy variables to create a ‘not working’ dummy variable. This collapsed variable was therefore used in all conditional models within the RTL subsample, split by median age, and no additional warnings were generated.

# Supplementary figures

Complete baseline cognitive test data, saliva sample provided for genotyping, and European ancestry
*N* = 7,877

Removed as not enough DNA available from saliva within 6 months of baseline cognitive testing
*n* = 648

Complete cognitive data on the PROTECT battery (4 timepoints) and CogTrack^TM^ (3 timepoints)^a^
*n* = 1,695

Removed as at least some missing data
*n* = 6,176

Removed as DNA samples were not extracted/available
*n* = 139

Removed
as did not survive QC
*n* = 54

Identified as an outlier
*n* =8

DNA available from saliva sample taken within 6 months of baseline data
*n* = 1,047

DNA sample available
*n* = 908

PCR data survived quality control
*n* = 846

PCR data survived quality control
*n* = 854

Supplementary Figure 1. Overview of the relative telomere length (RTL) workflow and exclusions at each stage of processing. RTL data was generated from DNA samples derived from saliva.
^a^ Only data from the PPROTECT battery is used in the current study, however data was also collected on another battery (CogTrack^TM^).
^b^ These participants were selected to minimise the number of plates from which samples were drawn. This was to minimise the workload of the lab team.

# Supplementary Tables

**Supplementary Table 1. Participant characteristics at baseline for the full PROTECT sample and subsamples included/excluded in our analyses.**

|  | Full PROTECT sample  (*N* = 25,542) | | Subsample excluded from our analyses (*N* = 17,665) | | Subsample included in our analyses (*n* = 7,877) | |
| --- | --- | --- | --- | --- | --- | --- |
| Age (mean, *SD*) | 62.33 | 9.41 | 62.27 | 10.36 | 62.55 | 7.12 |
| Sex (*n*, %) |  |  |  |  |  |  |
| Male | 6788 | 26.58 | 4817 | 27.27 | 1971 | 25.02 |
| Female | 18754 | 73.42 | 12848 | 72.73 | 5906 | 74.98 |
| Education level (*n*, %) |  |  |  |  |  |  |
| Secondary education | 4144 | 16.22 | 2960 | 16.76 | 1184 | 15.03 |
| Post-secondary education | 2989 | 11.70 | 2059 | 11.66 | 930 | 11.81 |
| Vocational qualification | 5146 | 20.15 | 3567 | 20.19 | 1579 | 20.05 |
| Undergraduate degree | 8157 | 31.94 | 5504 | 31.16 | 2653 | 33.68 |
| Post graduate degree | 4233 | 16.57 | 2949 | 16.69 | 1284 | 16.30 |
| Doctoral degree | 873 | 3.42 | 626 | 3.54 | 247 | 3.14 |
| Employment status (*n*, %) |  |  |  |  |  |  |
| Full-time | 5353 | 20.96 | 3939 | 22.30 | 1414 | 17.95 |
| Part-time | 4152 | 16.26 | 2816 | 15.94 | 1336 | 16.96 |
| Self-employed | 2675 | 10.47 | 1853 | 10.49 | 822 | 10.44 |
| Retired | 12551 | 49.14 | 8475 | 47.98 | 4076 | 51.75 |
| Unemployed | 805 | 3.15 | 576 | 3.26 | 229 | 2.91 |

*Note.* *SD* = standard deviation.

**Supplementary Table 2. Participant characteristics at baseline for the whole sample and subsample with relative telomere length (RTL) data.**

|  | Whole sample  (*N* = 7,877) | | RTL subsample (*n* = 846) | |
| --- | --- | --- | --- | --- |
| Age (mean, *SD*) | 62.55 | 7.12 | 62.75 | 6.87 |
| Sex (*n*, %) |  |  |  |  |
| Male | 1971 | 25.02 | 206 | 24.38 |
| Female | 5906 | 74.98 | 639 | 75.62 |
| Education level (*n*, %) |  |  |  |  |
| Secondary education | 1184 | 15.03 | 96 | 11.36 |
| Post-secondary education | 930 | 11.81 | 82 | 9.70 |
| Vocational qualification | 1579 | 20.05 | 174 | 20.59 |
| Undergraduate degree | 2653 | 33.68 | 306 | 36.21 |
| Post graduate degree | 1284 | 16.30 | 153 | 18.11 |
| Doctoral degree | 247 | 3.14 | 34 | 4.02 |
| Employment status (*n*, %) |  |  |  |  |
| Full-time | 1414 | 17.95 | 140 | 16.57 |
| Part-time | 1336 | 16.96 | 158 | 18.70 |
| Self-employed | 822 | 10.44 | 86 | 10.18 |
| Retired | 4076 | 51.75 | 437 | 51.72 |
| Unemployed | 229 | 2.91 | 24 | 2.84 |
| Baseline test session used in analyses (*n*, %)^a^ |  |  |  |  |
| 1 | 576 | 7.31 | 24 | 2.82 |
| 2 | 7301 | 92.69 | 827 | 97.18 |

*Note.* *SD* = standard deviation.

^a^Participants completed up to three test sessions at each timepoint. In our analyses, we used data from the second test session at baseline, unless this was not available, in which case we used data from the first test session. At all other timepoints, the first test session was used for all participants.

**Supplementary Table 3. Participant characteristics at baseline in the whole sample, split by median age (~62.24 years; *N* = 7,877).**

|  | Middle-aged  (*n* = 3,938) | | Older  (*n* = 3,939) | | Comparison  (*t/χ*^2^, *p*)*^a^* | |
| --- | --- | --- | --- | --- | --- | --- |
| Relative telomere length (mean, *SD*)^b^ | 0.05 | 0.98 | -0.05 | 1.01 | 1.48 | .139 |
| PGS-TL (mean, *SD*) | -0.01 | 1.01 | 0.01 | 0.99 | -0.50 | .614 |
| Age (mean, *SD*) | 56.78 | 3.46 | 68.31 | 4.78 | -122.62 | <.001 |
| Paired associate learning (baseline) | 4.69 | 0.94 | 4.40 | 0.96 | 13.84 | <.001 |
| Digit span test (baseline) | 7.59 | 1.61 | 7.31 | 1.77 | 7.53 | <.001 |
| Self-ordered search (baseline) | 8.07 | 2.39 | 7.37 | 2.44 | 10.86 | <.001 |
| Verbal reasoning (baseline) | 34.47 | 9.74 | 30.63 | 9.35 | 15.70 | <.001 |
| Sex (*n*, %) |  |  |  |  | 130.86 | <.001 |
| Male | 765 | 19.43% | 1206 | 30.62% |  |  |
| Female | 3173 | 80.57% | 2733 | 69.38% |  |  |
| Education level (*n*, %) |  |  |  |  | 88.97 | <.001 |
| Secondary education | 472 | 11.99% | 712 | 18.08% |  |  |
| Post-secondary education | 494 | 12.54% | 436 | 11.07% |  |  |
| Vocational qualification | 742 | 18.84% | 837 | 21.25% |  |  |
| Undergraduate degree | 1443 | 36.64% | 1210 | 30.72% |  |  |
| Post graduate degree | 682 | 17.32% | 602 | 15.28% |  |  |
| Doctoral degree | 105 | 2.67% | 142 | 3.60% |  |  |
| Employment status (*n*, %) |  |  |  |  | 2802.90 | <.001 |
| Full-time | 1250 | 31.74% | 164 | 4.16% |  |  |
| Part-time | 1009 | 25.62% | 327 | 8.30% |  |  |
| Self-employed | 572 | 14.53% | 250 | 6.35% |  |  |
| Retired | 887 | 22.52% | 3189 | 80.96% |  |  |
| Unemployed | 220 | 5.59% | 9 | 0.23% |  |  |
| Baseline test session*^c^* (*n*, %) |  |  |  |  | 1.58 | .208 |
| 1 | 303 | 7.69% | 273 | 6.93% |  |  |
| 2 | 3635 | 92.31% | 3666 | 93.07% |  |  |

*Note.* PGS-TL = polygenic score for telomere length; SD = standard deviation. Relative telomere length (log-transformed, adjusted for batch) and PGS-TL were standardised in the whole sample to a mean of 0 and SD of 1. Outliers of +/- 3.29 SDs from the mean (adjusting for covariates) were removed from the relative telomere length variable (see, Methods).

^a^ *t*- values and p-values are reported with Welch’s correction (Welch, 1951) for continuous variables and Pearson's Chi-squared test with Yates' continuity correction is reported for categorical variables.

^b^ Only a subsample of complete cases had relative telomere length data generated (*n* = 846); here the sample size is 399 for middle-aged individuals and 447 for older individuals.

*^c^* Participants completed up to three test sessions at each timepoint. In our analyses, we used data from the second test session at baseline, unless this was not available, in which case we used data from the first test session. At all other timepoints, the first test session was used for all participants.

**Supplementary Table 4. Participant characteristics at baseline in the sample with relative telomere length data, split by median age (~62.77 years; *N* = 846).**

|  | Middle-aged  (*n* = 423) | | Older  (*n* = 423) | | Comparison  (*t/χ*^2^, *p*)^a^ | |
| --- | --- | --- | --- | --- | --- | --- |
| Relative telomere length (mean, *SD*)^b^ | 0.06 | 1.00 | -0.06 | 1.00 | 1.62 | .106 |
| PGS-TL (mean, *SD*) | 0.01 | 1.02 | 0.07 | 0.95 | -0.50 | .614 |
| Age (mean, *SD*) | 57.18 | 3.59 | 68.32 | 4.42 | -40.25 | <.001 |
| Paired associate learning (baseline) | 4.74 | 0.88 | 4.46 | 0.91 | 4.40 | <.001 |
| Digit span test (baseline) | 7.61 | 1.40 | 7.42 | 1.67 | 1.79 | .074 |
| Self-ordered search (baseline) | 8.06 | 2.05 | 7.53 | 2.43 | 3.46 | .001 |
| Verbal reasoning (baseline) | 34.99 | 9.09 | 32.45 | 8.80 | 1.78 | .075 |
| Sex (*n*, %) |  |  |  |  | 27.86 | <.001 |
| Male | 70 | 16.55% | 137 | 32.39% |  |  |
| Female | 353 | 83.45% | 286 | 67.61% |  |  |
| Education level (*n*, %) |  |  |  |  | 9.58 | .088 |
| Secondary education | 35 | 8.27% | 61 | 14.42% |  |  |
| Post-secondary education | 45 | 10.64% | 37 | 8.75% |  |  |
| Vocational qualification | 88 | 20.80% | 86 | 20.33% |  |  |
| Undergraduate degree | 157 | 37.12% | 150 | 35.46% |  |  |
| Post graduate degree | 83 | 19.62% | 70 | 16.55% |  |  |
| Doctoral degree | 15 | 3.55% | 19 | 4.49% |  |  |
| Employment status (*n*, %) |  |  |  |  | 293.54 | <.001 |
| Full-time | 122 | 28.84% | 18 | 4.26% |  |  |
| Part-time | 121 | 28.61% | 37 | 8.75% |  |  |
| Self-employed | 62 | 14.66% | 24 | 5.67% |  |  |
| Retired | 96 | 22.70% | 342 | 80.85% |  |  |
| Unemployed | 22 | 5.20% | 2 | 0.47% |  |  |
| Baseline test session^c^ (*n*, %) |  |  |  |  | 0.04 | .836 |
| 1 | 11 | 2.60% | 13 | 3.07% |  |  |
| 2 | 412 | 97.40% | 410 | 96.93% |  |  |

*Note.* PGS-TL = polygenic score for telomere length; SD = standard deviation. Relative telomere length (log-transformed, adjusted for batch) and PGS-TL were standardised in the whole sample to a mean of 0 and SD of 1. Outliers of +/- 3.29 SDs from the mean (adjusting for covariates) were removed from the relative telomere length variable (see, Methods).

*^a^ t*- values and p-values are reported with Welch’s correction (Welch, 1951) for continuous variables and Pearson's Chi-squared test with Yates' continuity correction is reported for categorical variables.

^b^ Only a subsample of complete cases had relative telomere length data generated (*n* = 846).

*^c^* Participants completed up to three test sessions at each timepoint. In our analyses, we used data from the second test session at baseline, unless this was not available, in which case we used data from the first test session. At all other timepoints the first test session was used for all participants.

**Supplementary Table 5. Summary of regression results with batch (plate number) predicting relative telomere length [95% confidence intervals] (*N* = 846).**

| Predictors | B | SE B | B | p |
| --- | --- | --- | --- | --- |
| (Intercept) | -0.37 | 0.09 | -0.37 | <.001 |
| Batch (Plate number 1) | | | | |
| Plate number 2 | 0.32 | 0.13 | 0.32 | .013 |
| Plate number 3 | -0.07 | 0.13 | -0.07 | .578 |
| Plate number 4 | -0.09 | 0.14 | -0.09 | .484 |
| Plate number 5 | 0.50 | 0.13 | 0.50 | <.001 |
| Plate number 6 | -0.17 | 0.13 | -0.17 | .198 |
| Plate number 7 | 0.59 | 0.13 | 0.59 | <.001 |
| Plate number 8 | 0.56 | 0.13 | 0.56 | <.001 |
| Plate number 9 | 1.40 | 0.13 | 1.40 | <.001 |
| Plate number 10 | 1.02 | 0.17 | 1.02 | <.001 |
| Overall model fit | *F*(9, 836) = 27.41, *p* < .001, *R^2^ / R^2^* adjusted = 0.228 / 0.220 | | | |

*Note.* Relative telomere length was log-transformed, as it was not normally distributed, and Z-standardised. Batch (plate number) was dummy-coded with plate 1 as the reference category.

**Supplementary Table 6. Descriptive statistics for the cognitive outcomes at each time point for the whole sample and subsample with relative telomere length (RTL) data available.**

| Test | Whole sample (*N* = 7,877) | | | | | |  | RTL subsample (*n* = 846) | | | | | |
| --- | --- | --- | --- | --- | --- | --- | --- | --- | --- | --- | --- | --- | --- |
| Time point | n | M | SD | Range | Skew | Kurtosis |  | n | M | SD | Range | Skew | Kurtosis |
| Paired associate learning |  |  |  |  |  |  |  |  |  |  |  |  |  |
| Baseline | 7877 | 4.55 | 0.96 | (0-16) | 0.24 | 6.28 |  | 846 | 4.59 | 0.91 | (0-8) | -0.06 | 1.04 |
| Year one | 6757 | 4.61 | 0.95 | (0-11) | 0.01 | 1.69 |  | 846 | 4.64 | 0.89 | (2-8) | 0.25 | 0.22 |
| Year two | 5879 | 4.68 | 0.93 | (0-9) | 0.01 | 1.13 |  | 846 | 4.71 | 0.87 | (2-7) | 0.03 | -0.06 |
| Year three | 5042 | 4.72 | 0.94 | (0-10) | 0.12 | 1.26 |  | 846 | 4.69 | 0.87 | (2-7) | 0.1 | -0.14 |
| Digit span test |  |  |  |  |  |  |  |  |  |  |  |  |  |
| Baseline | 7877 | 7.45 | 1.7 | (0-20) | 1.23 | 9.14 |  | 846 | 7.51 | 1.54 | (0-16) | 0.15 | 2.57 |
| Year one | 6770 | 7.47 | 1.6 | (0-20) | 0.83 | 7.89 |  | 846 | 7.6 | 1.44 | (2-13) | 0.11 | 0.46 |
| Year two | 5891 | 7.57 | 1.57 | (0-20) | 0.39 | 5.87 |  | 846 | 7.64 | 1.42 | (4-13) | 0.21 | 0.16 |
| Year three | 5060 | 7.58 | 1.5 | (0-20) | 0.45 | 4.42 |  | 846 | 7.61 | 1.43 | (3-13) | 0.05 | 0.21 |
| Self-ordered search |  |  |  |  |  |  |  |  |  |  |  |  |  |
| Baseline | 7877 | 7.72 | 2.44 | (0-18) | -1.21 | 2.75 |  | 846 | 7.79 | 2.27 | (0-13) | -1.27 | 3.11 |
| Year one | 6746 | 7.5 | 2.82 | (0-20) | -1.21 | 1.77 |  | 846 | 7.63 | 2.57 | (0-15) | -1.33 | 2.44 |
| Year two | 5855 | 7.62 | 2.68 | (0-16) | -1.25 | 2.23 |  | 846 | 7.68 | 2.55 | (0-15) | -1.32 | 2.64 |
| Year three | 5014 | 7.67 | 2.64 | (0-20) | -1.25 | 2.47 |  | 846 | 7.58 | 2.61 | (0-13) | -1.41 | 2.45 |
| Verbal reasoning |  |  |  |  |  |  |  |  |  |  |  |  |  |
| Baseline | 7877 | 32.55 | 9.74 | (-9-68) | -0.05 | 0.22 |  | 846 | 33.66 | 9.07 | (8-68) | 0.07 | 0.05 |
| Year one | 6754 | 35.06 | 10.07 | (-4-76) | -0.02 | 0.22 |  | 846 | 36.12 | 9.28 | (8-69) | 0.02 | 0.04 |
| Year two | 5871 | 37.02 | 10.44 | (-9-80) | 0.03 | 0.33 |  | 846 | 37.81 | 9.67 | (1-76) | 0.03 | 0.42 |
| Year three | 5035 | 37.7 | 10.66 | (-9-77) | -0.01 | 0.29 |  | 846 | 38.47 | 10.16 | (-3-73) | -0.04 | 0.42 |

*Note.* *M* = mean; *SD* = standard deviation. The range shows the minimum and maximum score(s) within the data. The range of the possible scores that could be achieved on each test was 0-16 on the paired associates learning, 0-20 on the digit span test and self-ordered search, and on the verbal reasoning upper or lower limit.

**Supplementary Table 7. Descriptive statistics for the cognitive outcomes at each time point for the whole sample split by median age (~62.24 years; *N* = 7,877).**

| Test | Middle-age sample (*n* = 3,938) | | | | | |  | Older subsample (*n* = 3,939) | | | | | |
| --- | --- | --- | --- | --- | --- | --- | --- | --- | --- | --- | --- | --- | --- |
| Time point | n | M | SD | Range | Skew | Kurtosis |  | n | M | SD | Range | Skew | Kurtosis |
| Paired associate learning |  |  |  |  |  |  |  |  |  |  |  |  |  |
| Baseline | 3938 | 4.69 | 0.94 | (0-16) | 0.35 | 7.53 |  | 3939 | 4.4 | 0.96 | (0-15) | 0.16 | 5.6 |
| Year one | 3363 | 4.75 | 0.92 | (0-10) | 0.12 | 1.39 |  | 3394 | 4.48 | 0.96 | (0-11) | -0.06 | 1.95 |
| Year two | 2925 | 4.84 | 0.91 | (0-9) | 0.09 | 0.83 |  | 2954 | 4.52 | 0.93 | (0-9) | -0.04 | 1.45 |
| Year three | 2485 | 4.87 | 0.92 | (0-9) | 0.17 | 0.73 |  | 2557 | 4.58 | 0.94 | (0-10) | 0.09 | 1.79 |
| Digit span test |  |  |  |  |  |  |  |  |  |  |  |  |  |
| Baseline | 3938 | 7.59 | 1.61 | (0-20) | 1.28 | 8 |  | 3939 | 7.31 | 1.77 | (0-20) | 1.25 | 10.06 |
| Year one | 3368 | 7.61 | 1.48 | (0-20) | 0.48 | 3.57 |  | 3402 | 7.32 | 1.7 | (0-20) | 1.13 | 10.48 |
| Year two | 2928 | 7.72 | 1.49 | (0-20) | 0.15 | 3.91 |  | 2963 | 7.43 | 1.63 | (0-20) | 0.63 | 7.47 |
| Year three | 2493 | 7.74 | 1.43 | (3-20) | 0.57 | 3.27 |  | 2567 | 7.42 | 1.55 | (0-20) | 0.41 | 5.29 |
| Self-ordered search |  |  |  |  |  |  |  |  |  |  |  |  |  |
| Baseline | 3938 | 8.07 | 2.39 | (0-18) | -1.23 | 3.35 |  | 3939 | 7.37 | 2.44 | (0-15) | -1.25 | 2.36 |
| Year one | 3356 | 7.78 | 2.88 | (0-20) | -1.26 | 1.96 |  | 3390 | 7.22 | 2.73 | (0-18) | -1.23 | 1.69 |
| Year two | 2912 | 7.97 | 2.71 | (0-16) | -1.36 | 2.56 |  | 2943 | 7.27 | 2.59 | (0-16) | -1.24 | 2.16 |
| Year three | 2471 | 8.08 | 2.56 | (0-16) | -1.39 | 3.07 |  | 2543 | 7.28 | 2.65 | (0-20) | -1.18 | 2.2 |
| Verbal reasoning |  |  |  |  |  |  |  |  |  |  |  |  |  |
| Baseline | 3938 | 34.47 | 9.74 | (0-68) | -0.01 | 0.17 |  | 3939 | 30.63 | 9.35 | (-9-66) | -0.15 | 0.21 |
| Year one | 3361 | 37.08 | 10.1 | (-2-76) | -0.06 | 0.22 |  | 3393 | 33.05 | 9.64 | (-4-69) | -0.04 | 0.27 |
| Year two | 2921 | 39.12 | 10.54 | (-3-77) | -0.02 | 0.27 |  | 2950 | 34.94 | 9.93 | (-9-80) | 0 | 0.46 |
| Year three | 2483 | 40.04 | 10.72 | (-3-76) | -0.06 | 0.24 |  | 2552 | 35.43 | 10.09 | (-9-77) | -0.04 | 0.42 |

*Note.* *M* = mean; *SD* = standard deviation. The range shows the minimum and maximum score(s) within the data. The range of the possible scores that could be achieved on each test was 0-16 on the paired associates learning, 0-20 on the digit span test and self-ordered search, and on the verbal reasoning upper or lower limit.

**Supplementary Table 8. Descriptive statistics for the cognitive outcomes at each time point split by median age in the subsample with relative telomere length data (~62.77 years; *N* = 846).**

| Test | Middle-age sample (*n* = 423) | | | | | |  | Older subsample (*n* = 423) | | | | | |
| --- | --- | --- | --- | --- | --- | --- | --- | --- | --- | --- | --- | --- | --- |
| Time point | n | M | SD | Range | Skew | Kurtosis |  | n | M | SD | Range | Skew | Kurtosis |
| Paired associate learning |  |  |  |  |  |  |  |  |  |  |  |  |  |
| Baseline | 423 | 4.74 | 0.88 | (2-8) | 0.06 | 0.41 |  | 423 | 4.46 | 0.91 | (0-8) | -0.15 | 1.58 |
| Year one | 423 | 4.7 | 0.85 | (3-8) | 0.41 | 0.62 |  | 423 | 4.59 | 0.92 | (2-7) | 0.13 | -0.15 |
| Year two | 423 | 4.83 | 0.88 | (2-7) | 0.03 | -0.31 |  | 423 | 4.59 | 0.84 | (2-7) | -0.02 | 0.18 |
| Year three | 423 | 4.78 | 0.88 | (2-7) | 0.01 | -0.17 |  | 423 | 4.6 | 0.86 | (2-7) | 0.16 | -0.07 |
| Digit span test |  |  |  |  |  |  |  |  |  |  |  |  |  |
| Baseline | 423 | 7.61 | 1.4 | (4-14) | 0.4 | 1.29 |  | 423 | 7.42 | 1.67 | (0-16) | 0.04 | 2.93 |
| Year one | 423 | 7.73 | 1.43 | (4-13) | 0.02 | 0.15 |  | 423 | 7.47 | 1.44 | (2-13) | 0.19 | 0.83 |
| Year two | 423 | 7.74 | 1.43 | (4-12) | 0.04 | 0.2 |  | 423 | 7.56 | 1.41 | (4-13) | 0.38 | 0.19 |
| Year three | 423 | 7.73 | 1.42 | (3-12) | -0.07 | 0.06 |  | 423 | 7.51 | 1.44 | (3-13) | 0.17 | 0.43 |
| Self-ordered search |  |  |  |  |  |  |  |  |  |  |  |  |  |
| Baseline | 423 | 8.06 | 2.05 | (0-12) | -1.43 | 4.28 |  | 423 | 7.53 | 2.43 | (0-13) | -1.11 | 2.31 |
| Year one | 423 | 7.85 | 2.64 | (0-15) | -1.42 | 2.69 |  | 423 | 7.4 | 2.52 | (0-14) | -1.28 | 2.16 |
| Year two | 423 | 7.96 | 2.58 | (0-15) | -1.44 | 3.06 |  | 423 | 7.41 | 2.5 | (0-13) | -1.25 | 2.37 |
| Year three | 423 | 7.94 | 2.42 | (0-13) | -1.49 | 3.46 |  | 423 | 7.22 | 2.76 | (0-13) | -1.31 | 1.65 |
| Verbal reasoning |  |  |  |  |  |  |  |  |  |  |  |  |  |
| Baseline | 423 | 34.99 | 9.09 | (11-68) | 0.17 | 0.16 |  | 423 | 32.45 | 8.8 | (8-57) | -0.05 | -0.17 |
| Year one | 423 | 37.93 | 9.21 | (11-69) | 0.03 | 0.22 |  | 423 | 34.45 | 8.92 | (8-59) | -0.01 | -0.14 |
| Year two | 423 | 39.86 | 9.94 | (1-76) | -0.03 | 0.69 |  | 423 | 35.89 | 8.88 | (10-64) | -0.03 | 0.1 |
| Year three | 423 | 40.49 | 10.35 | (-3-73) | -0.12 | 0.64 |  | 423 | 36.56 | 9.54 | (8-62) | -0.05 | 0.29 |

*Note.* *M* = mean; *SD* = standard deviation. The range shows the minimum and maximum score(s) within the data. The range of the possible scores that could be achieved on each test was 0-16 on the paired associates learning, 0-20 on the digit span test and self-ordered search, and on the verbal reasoning upper or lower limit.

**Supplementary Table 9. Number and percentage of missing data points for each cognitive outcome *(N* = 7,877).**

|  | Baseline | | Year 1 | | Year 2 | | Year 3 | |
| --- | --- | --- | --- | --- | --- | --- | --- | --- |
| Cognitive outcome | n | % | n | % | n | % | n | % |
| Paired associates learning | 0 | 0.00 | 1120 | 14.22 | 1998 | 25.36 | 2835 | 35.99 |
| Digit span test | 0 | 0.00 | 1107 | 14.05 | 1986 | 25.21 | 2817 | 35.76 |
| Self-ordered search | 0 | 0.00 | 1131 | 14.36 | 2022 | 25.67 | 2863 | 36.35 |
| Verbal reasoning | 0 | 0.00 | 1123 | 14.26 | 2006 | 25.47 | 2842 | 36.08 |

*Note.* Participants were selected who had no missing data at baseline (see, Methods).

**Supplementary Table 10. Participant characteristics at baseline split by case completeness (*N* = 7,877).**

|  | Complete cases^a^ (*n* = 4,722) | | Cases with missingness  (*n* = 3,155) | | Comparison  (*t/χ*^2^, *p*)^b^ | |
| --- | --- | --- | --- | --- | --- | --- |
| Relative telomere length (mean, *SD*)^c^ | 0.00 | 1.00 | - | - | - | - |
| PRS-TL (mean, *SD*) | -0.01 | 1.01 | 0.02 | 0.99 | 1.29 | .198 |
| Age (mean, *SD*) | 62.74 | 7.06 | 62.27 | 7.19 | -2.87 | .004 |
| Paired associate learning (baseline) | 4.56 | 0.93 | 4.52 | 0.99 | 1.99 | .047 |
| Digit span test (baseline) | 7.46 | 1.61 | 7.43 | 1.83 | 0.71 | .481 |
| Self-ordered search (baseline) | 7.77 | 2.37 | 7.65 | 2.53 | -2.07 | .039 |
| Verbal reasoning (baseline) | 33.06 | 9.60 | 31.80 | 9.89 | -5.60 | <.001 |
| Sex (*n*, %) |  |  |  |  | 23.36 | <.001 |
| Male | 1090 | 23.08% | 881 | 27.92% |  |  |
| Female | 3632 | 76.92% | 2274 | 72.08% |  |  |
| Education level (*n*, %) |  |  |  |  | 30.52 | <.001 |
| Secondary education | 644 | 13.64% | 540 | 17.12% |  |  |
| Post-secondary education | 564 | 11.94% | 366 | 11.60% |  |  |
| Vocational qualification | 932 | 19.74% | 647 | 20.51% |  |  |
| Undergraduate degree | 1622 | 34.35% | 1031 | 32.68% |  |  |
| Post graduate degree | 785 | 16.62% | 499 | 15.82% |  |  |
| Doctoral degree | 175 | 3.71% | 72 | 2.28% |  |  |
| Employment status (*n*, %) |  |  |  |  | 31.89 | <.001 |
| Full-time | 768 | 16.26% | 646 | 20.48% |  |  |
| Part-time | 819 | 17.34% | 517 | 16.39% |  |  |
| Self-employed | 468 | 9.91% | 354 | 11.22% |  |  |
| Retired | 2535 | 53.68% | 1541 | 48.84% |  |  |
| Unemployed | 132 | 2.80% | 97 | 3.07% |  |  |
| Baseline test session^d^ (*n*, %) |  |  |  |  | 182.12 | <.001 |
| 1 | 192 | 4.07% | 384 | 12.17% |  |  |
| 2 | 4530 | 95.93% | 2771 | 87.83% |  |  |

*Note.* PRS-TL = polygenic risk score for telomere length; *SD* = standard deviation. Relative telomere length (log-transformed, adjusted for batch) and PRS-TL were standardised in the whole sample to a mean of 0 and *SD* of 1. Outliers of +/- 3.29 SDs from the mean (adjusting for covariates) were removed from the relative telomere length variable (see, Methods).

^a^ Complete data on all cognitive outcomes (i.e., four repeated measures on all four tests).

^b^ *t*- values and p-values are reported with Welch’s correction (Welch, 1951) for continuous variables and Pearson's Chi-squared test with Yates' continuity correction is reported for categorical variables.

^c^ Only a subsample of complete cases had relative telomere length data generated (*n* = 846).

^d^ Participants completed up to three test sessions at each timepoint. In our analyses, we used data from the second test session at baseline, unless this was not available, in which case we used data from the first test session. At all other timepoints the first test session was used for all participants.

## Unconditional latent growth models

**Supplementary Table 11. Fit Indices for the unconditional latent growth models for each of the cognitive outcomes (whole sample, *N* = 7,877).**

| Test  Model | χ^2^(*df*) | *p*-value | RMSEA | SRMR | CFI | TLI | AIC | χ^2^/*df* |
| --- | --- | --- | --- | --- | --- | --- | --- | --- |
| Paired associate learning |  |  |  |  |  |  |  |  |
| 1. Intercept (no growth) | 138.24 (8) | .000 | 0.05 | 0.04 | 0.93 | 0.95 | 67471.59 | 17.28 |
| 2. Linear | 2.79 (5) | .733 | <0.01 | 0.01 | 1.00 | 1.00 | 67308.46 | 0.56 |
| Digit span test |  |  |  |  |  |  |  |  |
| 1. Intercept (no growth) | 47.11 (8) | .000 | 0.03 | 0.02 | 0.98 | 0.99 | 88725.52 | 5.89 |
| 2. Linear | 19.58 (5) | .001 | 0.02 | 0.01 | 0.99 | 0.99 | 88669.40 | 3.92 |
| Self-ordered search |  |  |  |  |  |  |  |  |
| 1. Intercept (no growth) | 116.11 (8) | .000 | 0.04 | 0.04 | 0.94 | 0.96 | 118371.07 | 14.51 |
| 2. Linear | 64.37 (5) | .000 | 0.04 | 0.03 | 0.97 | 0.96 | 118309.83 | 12.87 |
| Verbal reasoning |  |  |  |  |  |  |  |  |
| 1. Intercept (no growth) | 2537.14 (8) | .000 | 0.20 | 0.13 | 0.81 | 0.86 | 176051.21 | 317.14 |
| 2. Linear | 217.69 (5) | .000 | 0.07 | 0.03 | 0.98 | 0.98 | 173383.07 | 43.54 |
| 3. Quadratic | 8.08 (1) | .004 | 0.03 | <0.01 | 1.00 | 1.00 | 173156.31 | 8.08 |

*Note.* χ^2^ = chi-square statistic; SRMR = Standardized Root Mean Squared Residual; RMSEA = root mean square error of approximation; CFI = comparative fit index; TLI = Tucker–Lewis index; AIC = Akaike Information Criterion. All models had freely estimated residual terms (heteroscedastic) and used full information maximum likelihood for missing data with robust maximum likelihood estimation (see Methods for more information).

**Supplementary Table 12. Parameter estimates [95% confidence intervals] for the unconditional latent growth models for each of the cognitive outcomes (whole sample, *N* = 7,877).**

|  | Paired associates learning | | Digit span test | | Self-ordered search | | Verbal reasoning | | |
| --- | --- | --- | --- | --- | --- | --- | --- | --- | --- |
|  | No growth | Linear | No growth | Linear | No growth | Linear | No growth | Linear | Quadratic |
| Parameter | Estimate | Estimate | Estimate | Estimate | Estimate | Estimate | Estimate | Estimate | Estimate |
| Means | | | | | | | | | |
| Intercept | 4.62 [4.60, 4.64] | 4.55 [4.53, 4.57] | 7.5 [7.46, 7.54] | 7.44 [7.40, 7.48] | 7.63 [7.59, 7.67] | 7.66 [7.60, 7.72] | 34.98 [34.76, 35.20] | 32.92 [32.7, 33.14] | 32.52 [32.30, 32.74] |
| Slope | - | 0.06 [0.06, 0.06] | - | 0.04 [0.02, 0.06] | - | -0.03 [-0.05, -0.01] | - | 1.55 [1.49, 1.61] | 2.83 [2.63, 3.03] |
| Slope^2^ | - | - | - | - | - | - | - | - | -0.43 [-0.49, -0.37] |
| Variances | | | | | | | | | |
| Intercept | 0.27 [0.25, 0.29] | 0.27 [0.23, 0.31] | 1.47 [1.33, 1.61] | 1.58 [1.38, 1.78] | 2.47 [2.31, 2.63] | 2.32 [2.07, 2.57] | 79.75 [76.79, 82.71] | 69.24 [66.24, 72.24] | 71.47 [65.61, 77.33] |
| Slope | - | 0.01 [0.01, 0.01] | - | 0.03 [0.01, 0.05] | - | 0.12 [0.06, 0.18] | - | 1.31 [0.96, 1.66] | 11.72 [5.27, 18.17] |
| Slope^2^ | - | - | - | - | - | - | - | - | 0.82 [0.39, 1.25] |
| Covariances | | | | | | | | | |
| Intercept-Slope | - | -0.01 [-0.03, 0.01] | - | -0.06 [-0.12, 0.00] | - | 0.00 [-0.10, 0.10] | - | 2.95 [2.19, 3.71] | 0.51 [-5.74, 6.76] |
| Intercept-Slope^2^ | - | - | - | - | - | - | - | - | 0.31 [-1.24, 1.86] |
| Slope-Slope^2^ | - | - | - | - | - | - | - | - | -2.86 [-4.35, -1.37] |
| Residual variances | | | | | | | | | |
| T1 | 0.67 [0.63, 0.71] | 0.64 [0.58, 0.70] | 1.44 [1.28, 1.6] | 1.33 [1.15, 1.51] | 3.8 [3.55, 4.05] | 3.71 [3.4, 4.02] | 40.34 [38.40, 42.28] | 28.20 [26.40, 30.00] | 23.34 [17.81, 28.87] |
| T2 | 0.64 [0.60, 0.68] | 0.64 [0.60, 0.68] | 1.11 [0.99, 1.23] | 1.04 [0.92, 1.16] | 5.37 [5.02, 5.72] | 5.42 [5.07, 5.77] | 20.93 [19.66, 22.2] | 22.92 [21.69, 24.15] | 22.08 [20.04, 24.12] |
| T3 | 0.6 [0.56, 0.64] | 0.58 [0.54, 0.62] | 1.04 [0.92, 1.16] | 1.04 [0.92, 1.16] | 4.46 [4.13, 4.79] | 4.28 [3.95, 4.61] | 25.94 [24.41, 27.47] | 23.22 [21.79, 24.65] | 21.05 [18.95, 23.15] |
| T4 | 0.61 [0.57, 0.65] | 0.55 [0.51, 0.59] | 0.96 [0.84, 1.08] | 0.87 [0.71, 1.03] | 4.43 [4.08, 4.78] | 3.78 [3.37, 4.19] | 30.04 [28.32, 31.76] | 19.8 [17.78, 21.82] | 18.33 [11.82, 24.84] |

*Note.* All models had freely estimated residual terms (heteroscedastic) and used full information maximum likelihood for missing data with robust maximum likelihood estimation (see Methods for more information).

**Supplementary Table 13. Fit Indices for the unconditional latent growth models for each of the cognitive outcomes (relative telomere length subsample, *N* = 846).**

| Test  Model | χ^2^(*df*) | *p*-value | RMSEA | SRMR | CFI | TLI | AIC | χ^2^/*df* |
| --- | --- | --- | --- | --- | --- | --- | --- | --- |
| Paired associate learning |  |  |  |  |  |  |  |  |
| 1. Intercept (no growth) | 15.93 (8) | .043 | 0.03 | 0.03 | 0.97 | 0.98 | 8499.21 | 1.99 |
| 2. Linear | 3.11 (5) | .683 | <0.01 | 0.02 | 1.00 | 1.01 | 8491.84 | 0.62 |
| Digit span test |  |  |  |  |  |  |  |  |
| 1. Intercept (no growth) | 14.91 (8) | .061 | 0.03 | 0.03 | 0.99 | 0.99 | 10762.41 | 1.86 |
| 2. Linear | 5.95 (5) | .311 | 0.02 | 0.02 | 1.00 | 1.00 | 10758.25 | 1.19 |
| Self-ordered search |  |  |  |  |  |  |  |  |
| 1. Intercept (no growth) | 14.39 (8) | .072 | 0.03 | 0.04 | 0.97 | 0.98 | 15380.21 | 1.80 |
| 2. Linear | 5.31 (5) | .380 | 0.01 | 0.02 | 1.00 | 1.00 | 15375.16 | 1.06 |
| Verbal reasoning |  |  |  |  |  |  |  |  |
| 1. Intercept (no growth) | 372.73 (8) | .000 | 0.23 | 0.14 | 0.80 | 0.85 | 22892.97 | 46.59 |
| 2. Linear | 35.06 (5) | .000 | 0.08 | 0.03 | 0.98 | 0.98 | 22510.83 | 7.01 |
| 3. Quadratic | 0.13 (1) | .721 | <0.01 | 0.00 | 1.00 | 1.00 | 22479.13 | 0.13 |

*Note.* χ^2^ = chi-square statistic; SRMR = Standardized Root Mean Squared Residual; RMSEA = root mean square error of approximation; CFI = comparative fit index; TLI = Tucker–Lewis index; AIC = Akaike Information Criterion. All models had freely estimated residual terms (heteroscedastic) and used full information maximum likelihood for missing data with robust maximum likelihood estimation (see Methods for more information).

**Supplementary Table 14. Parameter estimates [95% confidence intervals] for the unconditional latent growth models for each of the cognitive outcomes (relative telomere length subsample, *N* = 846).**

|  | Paired associates learning | | Digit span test | | Self-ordered search | | Verbal reasoning | | |
| --- | --- | --- | --- | --- | --- | --- | --- | --- | --- |
|  | No growth | Linear | No growth | Linear | No growth | Linear | No growth | Linear | Quadratic |
| Parameter | Estimate | Estimate | Estimate | Estimate | Estimate | Estimate | Estimate | Estimate | Estimate |
| Means | | | | | | | | | |
| Intercept | 4.66 [4.62, 4.70] | 4.61 [4.55, 4.67] | 7.6 [7.52, 7.68] | 7.55 [7.45, 7.65] | 7.68 [7.56, 7.80] | 7.77 [7.63, 7.91] | 36.76 [36.17, 37.35] | 34.22 [33.63, 34.81] | 33.70 [33.09, 34.31] |
| Slope | - | 0.03 [0.01, 0.05] | - | 0.03 [-0.01, 0.07] | - | -0.06 [-0.12, 0.00] | - | 1.6 [1.44, 1.76] | 2.98 [2.45, 3.51] |
| Slope^2^ | - | - | - | - | - | - | - | - | -0.46 [-0.64, -0.28] |
| Variances | | | | | | | | | |
| Intercept | 0.21 [0.17, 0.25] | 0.24 [0.16, 0.32] | 1.26 [1.10, 1.42] | 1.33 [1.11, 1.55] | 2.06 [1.71, 2.41] | 1.83 [1.20, 2.46] | 66.12 [58.91, 73.33] | 55.7 [48.43, 62.97] | 59.46 [45.21, 73.71] |
| Slope | - | 0.02 [0.00, 0.04] | - | 0.03 [-0.01, 0.07] | - | 0.07 [-0.07, 0.21] | - | 0.73 [-0.05, 1.51] | 13.35 [-2.60, 29.30] |
| Slope^2^ | - | - | - | - | - | - | - | - | 1.42 [0.38, 2.46] |
| Covariances | | | | | | | | | |
| Intercept-Slope | - | -0.02 [-0.06, 0.02] | - | -0.05 [-0.13, 0.03] | - | 0.04 [-0.20, 0.28] | - | 2.97 [1.21, 4.73] | -1.67 [-16.49, 13.15] |
| Intercept-Slope^2^ | - | - | - | - | - | - | - | - | 1.09 [-2.63, 4.81] |
| Slope-Slope^2^ | - | - | - | - | - | - | - | - | -4.04 [-7.76, -0.32] |
| Residual variances | | | | | | | | | |
| T1 | 0.63 [0.53, 0.73] | 0.58 [0.48, 0.68] | 1.20 [0.93, 1.47] | 1.09 [0.80, 1.38] | 3.34 [2.65, 4.03] | 3.32 [2.50, 4.14] | 40.19 [35.6, 44.78] | 27.09 [23.01, 31.17] | 21.99 [9.03, 34.95] |
| T2 | 0.57 [0.51, 0.63] | 0.57 [0.51, 0.63] | 0.79 [0.67, 0.91] | 0.70 [0.58, 0.82] | 4.70 [3.80, 5.60] | 4.76 [3.86, 5.66] | 20.67 [17.32, 24.02] | 22.23 [19.05, 25.41] | 20.06 [15.73, 24.39] |
| T3 | 0.54 [0.48, 0.60] | 0.54 [0.48, 0.60] | 0.70 [0.60, 0.80] | 0.70 [0.60, 0.80] | 4.12 [3.30, 4.94] | 4.00 [3.20, 4.80] | 22.54 [18.72, 26.36] | 20.61 [16.94, 24.28] | 19.46 [14.11, 24.81] |
| T4 | 0.55 [0.49, 0.61] | 0.49 [0.41, 0.57] | 0.85 [0.67, 1.03] | 0.75 [0.57, 0.93] | 4.71 [3.79, 5.63] | 4.24 [3.26, 5.22] | 32.3 [28.14, 36.46] | 24.43 [19.92, 28.94] | 16.11 [1.72, 30.5] |

*Note.* All models had freely estimated residual terms (heteroscedastic) and used full information maximum likelihood for missing data with robust maximum likelihood estimation (see Methods for more information).

**Supplementary Table 15. Fit Indices for the unconditional latent growth models for each of the cognitive outcomes in individuals aged under ~62.24 years at baseline (*N* = 3,938).**

| Test  Model | χ^2^(*df*) | *p*-value | RMSEA | SRMR | CFI | TLI | AIC | χ^2^/*df* |
| --- | --- | --- | --- | --- | --- | --- | --- | --- |
| Paired associate learning |  |  |  |  |  |  |  |  |
| 1. Intercept (no growth) | 85.47 (8) | .000 | 0.05 | 0.04 | 0.91 | 0.93 | 33072.22 | 10.68 |
| 2. Linear | 5.79 (5) | .327 | 0.01 | 0.01 | 1.00 | 1.00 | 32982.31 | 1.16 |
| Digit Span test |  |  |  |  |  |  |  |  |
| 1. Intercept (no growth) | 25.06 (8) | .002 | 0.02 | 0.02 | 0.98 | 0.99 | 42442.79 | 3.13 |
| 2. Linear^a^ | 9.01 (6) | .173 | 0.01 | 0.01 | 1.00 | 1.00 | 42416.46 | 1.50 |
| Self-ordered search |  |  |  |  |  |  |  |  |
| 1. Intercept (no growth) | 96.58 (8) | .000 | 0.05 | 0.05 | 0.90 | 0.93 | 58768.22 | 12.07 |
| 2. Linear | 68.42 (5) | .000 | 0.06 | 0.04 | 0.93 | 0.92 | 58733.06 | 13.68 |
| Verbal reasoning |  |  |  |  |  |  |  |  |
| 1. Intercept (no growth) | 1366.17 (8) | .000 | 0.21 | 0.13 | 0.78 | 0.84 | 88201.48 | 170.77 |
| 2. Linear | 93.69 (5) | .000 | 0.07 | 0.03 | 0.99 | 0.98 | 86718.10 | 18.74 |
| 3. Quadratic | 2.78 (1) | 0.10 | 0.02 | <0.01 | 1.00 | 1.00 | 86626.89 | 2.78 |

*Note.* χ^2^ = chi-square statistic; SRMR = Standardized Root Mean Squared Residual; RMSEA = root mean square error of approximation; CFI = comparative fit index; TLI = Tucker–Lewis index; AIC = Akaike Information Criterion. All models had freely estimated residual terms (heteroscedastic) and used full information maximum likelihood for missing data with robust maximum likelihood estimation (see Methods for more information).

^a^ For the digit span test linear model, the variance for the slope variance was fixed to 0.0001 (i.e., estimated as a fixed effect; see Supplementary Methods S2), hence no confidence intervals are provided and no predictors are included.

**Supplementary Table 16. Parameter estimates [95% confidence intervals] for the unconditional latent growth models for each of the cognitive outcomes in individuals aged under ~62.24 years at baseline (*N* = 3,938).**

|  | Paired associates learning | | Digit span test | | Self-ordered search | | Verbal reasoning | | |
| --- | --- | --- | --- | --- | --- | --- | --- | --- | --- |
|  | No growth | Linear | No growth | Linear | No growth | Linear | No growth | Linear | Quadratic |
| Parameter | Estimate | Estimate | Estimate | Estimate | Estimate | Estimate | Estimate | Estimate | Estimate |
| Means | | | | | | | | | |
| Intercept | 4.77 [4.75, 4.79] | 4.69 [4.67, 4.71] | 7.65 [7.61, 7.69] | 7.58 [7.54, 7.62] | 7.98 [7.92, 8.04] | 8 [7.92, 8.08] | 37.02 [36.71, 37.33] | 34.8 [34.51, 35.09] | 34.44 [34.15, 34.73] |
| Slope | - | 0.06 [0.04, 0.08] | - | 0.05 [0.03, 0.07] | - | -0.01 [-0.05, 0.03] | - | 1.72 [1.62, 1.82] | 2.86 [2.57, 3.15] |
| Slope^2^ | - | - | - | - | - | - | - | - | -0.39 [-0.49, -0.29] |
| Variances | | | | | | | | | |
| Intercept | 0.24 [0.22, 0.26] | 0.28 [0.22, 0.34] | 1.33 [1.17, 1.49] | 1.36 [1.16, 1.56] | 2.47 [2.25, 2.69] | 2.39 [2.02, 2.76] | 78.52 [74.42, 82.62] | 68.59 [64.40, 72.78] | 70 [61.71, 78.29] |
| Slope | - | 0.02 [0.00, 0.04] | - | 0.00^a^ | - | 0.16 [0.06, 0.26] | - | 1.39 [0.86, 1.92] | 12.12 [2.91, 21.33] |
| Slope^2^ | - | - | - | - | - | - | - | - | 1.05 [0.42, 1.68] |
| Covariances | | | | | | | | | |
| Intercept-Slope | - | -0.03 [-0.05, -0.01] | - | -0.01 [-0.03, 0.01] | - | -0.04 [-0.20, 0.12] | - | 2.79 [1.69, 3.89] | 1.37 [-7.51, 10.25] |
| Intercept-Slope^2^ | - | - | - | - | - | - | - | - | 0.07 [-2.14, 2.28] |
| Slope-Slope^2^ | - | - | - | - | - | - | - | - | -3.24 [-5.38, -1.10] |
| Residual variances | | | | | | | | | |
| T1 | 0.65 [0.57, 0.73] | 0.59 [0.53, 0.65] | 1.27 [1.07, 1.47] | 1.26 [1.06, 1.46] | 3.59 [3.24, 3.94] | 3.41 [2.96, 3.86] | 42.06 [39.14, 44.98] | 28.69 [26, 31.38] | 24.86 [16.94, 32.78] |
| T2 | 0.61 [0.57, 0.65] | 0.61 [0.57, 0.65] | 0.88 [0.78, 0.98] | 0.92 [0.82, 1.02] | 5.71 [5.16, 6.26] | 5.77 [5.22, 6.32] | 22.38 [20.46, 24.3] | 24.54 [22.68, 26.4] | 22.89 [19.95, 25.83] |
| T3 | 0.58 [0.54, 0.62] | 0.57 [0.53, 0.61] | 0.92 [0.80, 1.04] | 0.92 [0.80, 1.04] | 4.51 [4.02, 5.00] | 4.31 [3.82, 4.8] | 27.76 [25.47, 30.05] | 24.49 [22.37, 26.61] | 22.71 [19.71, 25.71] |
| T4 | 0.62 [0.56, 0.68] | 0.55 [0.49, 0.61] | 0.86 [0.70, 1.02] | 0.86 [0.70, 1.02] | 4.14 [3.63, 4.65] | 3.35 [2.78, 3.92] | 33.78 [30.96, 36.60] | 21.31 [18.12, 24.50] | 17.10 [7.71, 26.49] |

*Note.* All models had freely estimated residual terms (heteroscedastic) and used full information maximum likelihood for missing data with robust maximum likelihood estimation (see Methods for more information).

^a^ For the digit span test model, the variance for the slope variance was fixed to 0.0001 (i.e., estimated as a fixed effect; see Supplementary Methods S2), hence no confidence intervals are provided and no predictors are included.

**Supplementary Table 17. Fit Indices for the unconditional latent growth models for each of the cognitive outcomes in individuals aged 62.24 years and over at baseline (*N* = 3,939).**

| Test  Model | χ^2^(*df*) | *p*-value | RMSEA | SRMR | CFI | TLI | AIC | χ^2^/*df* |
| --- | --- | --- | --- | --- | --- | --- | --- | --- |
| Paired associate learning |  |  |  |  |  |  |  |  |
| 1. Intercept (no growth) | 70.56 (8) | .000 | 0.05 | 0.04 | 0.92 | 0.94 | 34049.67 | 8.82 |
| 2. Linear | 2.98 (5) | .703 | <0.01 | 0.01 | 1.00 | 1.00 | 33970.15 | 0.60 |
| Digit span test |  |  |  |  |  |  |  |  |
| 1. Intercept (no growth) | 33.25 (8) | .000 | 0.03 | 0.03 | 0.98 | 0.98 | 45996.75 | 4.16 |
| 2. Linear | 8.66 (5) | .124 | 0.01 | 0.01 | 1.00 | 1.00 | 45955.42 | 1.73 |
| Self-ordered search |  |  |  |  |  |  |  |  |
| 1. Intercept (no growth) | 38.22 (8) | .000 | 0.03 | 0.03 | 0.97 | 0.98 | 59346.57 | 4.78 |
| 2. Linear | 14.99 (5) | .010 | 0.02 | 0.02 | 0.99 | 0.99 | 59324.11 | 3.00 |
| Verbal reasoning |  |  |  |  |  |  |  |  |
| 1. Intercept (no growth) | 1172.08 (8) | .000 | 0.19 | 0.13 | 0.83 | 0.87 | 87424.73 | 146.51 |
| 2. Linear | 131.19 (5) | .000 | 0.08 | 0.03 | 0.98 | 0.98 | 86250.54 | 26.24 |
| 3. Quadratic | 5.42 (1) | 0.02 | 0.03 | 0.01 | 1.00 | 1.00 | 86114.44 | 5.42 |

*Note.* χ^2^ = chi-square statistic; SRMR = Standardized Root Mean Squared Residual; RMSEA = root mean square error of approximation; CFI = comparative fit index; TLI = Tucker–Lewis index; AIC = Akaike Information Criterion. All models had freely estimated residual terms (heteroscedastic) and used full information maximum likelihood for missing data with robust maximum likelihood estimation (see Methods for more information).

**Supplementary Table 18. Parameter estimates [95% confidence intervals] for the unconditional latent growth models for each of the cognitive outcomes in individuals aged 62.24 years and over at baseline (*N* = 3,939).**

|  | Paired associates learning | | Digit span test | | Self-ordered search | | Verbal reasoning | | |
| --- | --- | --- | --- | --- | --- | --- | --- | --- | --- |
|  | No growth | Linear | No growth | Linear | No growth | Linear | No growth | Linear | Quadratic |
| Parameter | Estimate | Estimate | Estimate | Estimate | Estimate | Estimate | Estimate | Estimate | Estimate |
| Means | | | | | | | | | |
| Intercept | 4.48 [4.46, 4.50] | 4.41 [4.39, 4.43] | 7.35 [7.31, 7.39] | 7.3 [7.24, 7.36] | 7.28 [7.22, 7.34] | 7.33 [7.25, 7.41] | 32.95 [32.66, 33.24] | 31.05 [30.78, 31.32] | 30.60 [30.31, 30.89] |
| Slope | - | 0.06 [0.04, 0.08] | - | 0.04 [0.02, 0.06] | - | -0.04 [-0.08, 0.00] | - | 1.39 [1.31, 1.47] | 2.80 [2.53, 3.07] |
| Slope^2^ | - | - | - | - | - | - | - | - | -0.47 [-0.55, -0.39] |
| Variances | | | | | | | | | |
| Intercept | 0.26 [0.24, 0.28] | 0.22 [0.18, 0.26] | 1.55 [1.33, 1.77] | 1.77 [1.46, 2.08] | 2.23 [2.03, 2.43] | 2.08 [1.75, 2.41] | 72.48 [68.58, 76.38] | 62.93 [59.01, 66.85] | 65.11 [57.35, 72.87] |
| Slope | - | 0.00 [-0.02, 0.02] | - | 0.06 [0.02, 0.10] | - | 0.10 [0.00, 0.20] | - | 1.22 [0.73, 1.71] | 10.74 [2.02, 19.46] |
| Slope^2^ | - | - | - | - | - | - | - | - | 0.57 [-0.04, 1.18] |
| Covariances | | | | | | | | | |
| Intercept-Slope | - | 0.01 [-0.01, 0.03] | - | -0.11 [-0.19, -0.03] | - | 0.01 [-0.13, 0.15] | - | 2.46 [1.44, 3.48] | 0.06 [-8.31, 8.43] |
| Intercept-Slope^2^ | - | - | - | - | - | - | - | - | 0.27 [-1.81, 2.35] |
| Slope-Slope^2^ | - | - | - | - | - | - | - | - | -2.38 [-4.40, -0.36] |
| Residual variances | | | | | | | | | |
| T1 | 0.68 [0.62, 0.74] | 0.69 [0.61, 0.77] | 1.60 [1.38, 1.82] | 1.38 [1.13, 1.63] | 3.98 [3.65, 4.31] | 3.93 [3.50, 4.36] | 38.41 [35.84, 40.98] | 27.44 [25.05, 29.83] | 22.3 [14.95, 29.65] |
| T2 | 0.67 [0.61, 0.73] | 0.68 [0.62, 0.74] | 1.34 [1.1, 1.58] | 1.16 [0.92, 1.4] | 5.10 [4.65, 5.55] | 5.13 [4.68, 5.58] | 19.59 [17.92, 21.26] | 21.43 [19.82, 23.04] | 21.15 [18.45, 23.85] |
| T3 | 0.61 [0.55, 0.67] | 0.59 [0.55, 0.63] | 1.15 [0.95, 1.35] | 1.16 [0.96, 1.36] | 4.42 [3.97, 4.87] | 4.28 [3.83, 4.73] | 24.28 [22.24, 26.32] | 22.13 [20.21, 24.05] | 19.59 [16.71, 22.47] |
| T4 | 0.61 [0.55, 0.67] | 0.57 [0.51, 0.63] | 1.05 [0.89, 1.21] | 0.86 [0.66, 1.06] | 4.68 [4.19, 5.17] | 4.13 [3.58, 4.68] | 26.33 [24.29, 28.37] | 18.05 [15.54, 20.56] | 19.11 [10.43, 27.79] |

*Note.* All models had freely estimated residual terms (heteroscedastic) and used full information maximum likelihood for missing data with robust maximum likelihood estimation (see Methods for more information).

**Supplementary Table 19. Fit Indices for the unconditional latent growth models for each of the cognitive outcomes in individuals aged under ~62.77 years at baseline from the subsample with relative telomere data (*n* = 3,938).**

| Model | χ^2^(*df*) | *p*-value | RMSEA | SRMR | CFI | TLI | AIC | χ^2^/*df* |
| --- | --- | --- | --- | --- | --- | --- | --- | --- |
| Paired associate learning |  |  |  |  |  |  |  |  |
| 1. Intercept (no growth) | 10.54 (8) | .229 | 0.03 | 0.04 | 0.97 | 0.98 | 4245.71 | 1.32 |
| 2. Linear | 6.22 (5) | .286 | 0.02 | 0.03 | 0.99 | 0.98 | 4247.02 | 1.24 |
| Digit span test |  |  |  |  |  |  |  |  |
| 1. Intercept (no growth) | 12.06 (8) | .149 | 0.04 | 0.04 | 0.99 | 0.99 | 5255.72 | 1.51 |
| 2. Linear | 7.69 (5) | .174 | 0.04 | 0.03 | 0.99 | 0.99 | 5256.98 | 1.54 |
| Self-ordered search |  |  |  |  |  |  |  |  |
| 1. Intercept (no growth) | 22.63 (8) | .004 | 0.07 | 0.07 | 0.88 | 0.91 | 7574.53 | 2.83 |
| 2. Linear | 14.18 (5) | .014 | 0.07 | 0.05 | 0.92 | 0.91 | 7568.98 | 2.84 |
| Verbal reasoning |  |  |  |  |  |  |  |  |
| 1. Intercept (no growth) | 230.34 (8) | .000 | 0.26 | 0.16 | 0.76 | 0.82 | 11563.86 | 28.79 |
| 2. Linear | 27.11 (5) | .000 | 0.10 | 0.04 | 0.98 | 0.97 | 11334.20 | 5.42 |
| 3. Quadratic | 0.08 (1) | 0.77 | <0.01 | <0.01 | 1.00 | 1.01 | 11312.79 | 0.08 |

*Note.* χ^2^ = chi-square statistic; SRMR = Standardized Root Mean Squared Residual; RMSEA = root mean square error of approximation; CFI = comparative fit index; TLI = Tucker–Lewis index; AIC = Akaike Information Criterion. All models had freely estimated residual terms (heteroscedastic) and used full information maximum likelihood for missing data with robust maximum likelihood estimation (see Methods for more information).

**Supplementary Table 20. Parameter estimates [95% confidence intervals] for the unconditional latent growth models for each of the cognitive outcomes in individuals aged under ~62.77 years at baseline from the subsample with relative telomere length data (*n* = 423).**

|  | Paired associates learning | | Digit span test | | Self-ordered search | | Verbal reasoning | | |
| --- | --- | --- | --- | --- | --- | --- | --- | --- | --- |
|  | No growth | Linear | No growth | Linear | No growth | Linear | No growth | Linear | Quadratic |
| Parameter | Estimate | Estimate | Estimate | Estimate | Estimate | Estimate | Estimate | Estimate | Estimate |
| Means | | | | | | | | | |
| Intercept | 4.76 [4.7, 4.82] | 4.72 [4.64, 4.8] | 7.7 [7.58, 7.82] | 7.64 [7.5, 7.78] | 7.97 [7.81, 8.13] | 8.01 [7.81, 8.21] | 38.46 [37.6, 39.32] | 35.59 [34.77, 36.41] | 34.97 [34.11, 35.83] |
| Slope | - | 0.03 [-0.01, 0.07] | - | 0.04 [0, 0.08] | - | -0.03 [-0.11, 0.05] | - | 1.85 [1.61, 2.09] | 3.57 [2.81, 4.33] |
| Slope^2^ | - | - | - | - | - | - | - | - | -0.58 [-0.83, -0.33] |
| Variances | | | | | | | | | |
| Intercept | 0.17 [0.13, 0.21] | 0.18 [0.06, 0.3] | 1.23 [1.03, 1.43] | 1.29 [1.04, 1.54] | 1.85 [1.32, 2.38] | 1.31 [0.53, 2.09] | 66.97 [56.31, 77.63] | 56.14 [45.61, 66.67] | 56.51 [35.26, 77.76] |
| Slope | - | 0.01 [-0.01, 0.03] | - | 0.02 [-0.02, 0.06] | - | 0.06 [-0.14, 0.26] | - | 0.45 [-0.69, 1.59] | 10.03 [-12.98, 33.04] |
| Slope^2^ | - | - | - | - | - | - | - | - | 1.76 [0.33, 3.19] |
| Covariances | | | | | | | | | |
| Intercept-Slope | - | -0.01 [-0.05, 0.03] | - | -0.03 [-0.09, 0.03] | - | 0.16 [-0.15, 0.47] | - | 3.52 [1.09, 5.95] | 2.93 [-19.26, 25.12] |
| Intercept-Slope^2^ | - | - | - | - | - | - | - | - | 0.18 [-5.46, 5.82] |
| Slope-Slope^2^ | - | - | - | - | - | - | - | - | -3.98 [-9.29, 1.33] |
| Residual variances | | | | | | | | | |
| T1 | 0.61 [0.51, 0.71] | 0.59 [0.45, 0.73] | 0.81 [0.56, 1.06] | 0.73 [0.48, 0.98] | 2.77 [1.91, 3.63] | 2.95 [1.87, 4.03] | 42.48 [35.99, 48.97] | 26.83 [21.13, 32.53] | 25.93 [6.9, 44.96] |
| T2 | 0.57 [0.47, 0.67] | 0.57 [0.47, 0.67] | 0.74 [0.62, 0.86] | 0.76 [0.62, 0.9] | 5.23 [3.82, 6.64] | 5.3 [3.89, 6.71] | 20 [15.32, 24.68] | 22.4 [17.95, 26.85] | 18.04 [11.83, 24.25] |
| T3 | 0.59 [0.49, 0.69] | 0.58 [0.48, 0.68] | 0.76 [0.6, 0.92] | 0.76 [0.6, 0.92] | 4.13 [2.93, 5.33] | 3.93 [2.79, 5.07] | 26.8 [20.43, 33.17] | 23.76 [17.78, 29.74] | 24.08 [15.5, 32.66] |
| T4 | 0.59 [0.49, 0.69] | 0.53 [0.39, 0.67] | 0.88 [0.64, 1.12] | 0.82 [0.55, 1.09] | 3.87 [2.67, 5.07] | 3.32 [2.05, 4.59] | 36.46 [29.64, 43.28] | 27.88 [20.71, 35.05] | 10.83 [-8.32, 29.98] |

*Note.* All models had freely estimated residual terms (heteroscedastic) and used full information maximum likelihood for missing data with robust maximum likelihood estimation (see Methods for more information).

**Supplementary Table 21. Fit Indices for the unconditional latent growth models for each of the cognitive outcomes in individuals aged 62.77 years and over at baseline (*n* = 423).**

| Model | χ^2^(*df*) | *p*-value | RMSEA | SRMR | CFI | TLI | AIC | χ^2^/*df* |
| --- | --- | --- | --- | --- | --- | --- | --- | --- |
| Paired associate learning |  |  |  |  |  |  |  |  |
| 1. Intercept (no growth) | 20.95 (8) | .007 | 0.06 | 0.06 | 0.92 | 0.94 | 4235.44 | 2.62 |
| 2. Linear | 11.35 (5) | .045 | 0.06 | 0.04 | 0.96 | 0.96 | 4232.22 | 2.27 |
| Digit span test |  |  |  |  |  |  |  |  |
| 1. Intercept (no growth) | 8.64 (8) | .374 | 0.01 | 0.03 | 1.00 | 1.00 | 5476.57 | 1.08 |
| 2. Linear | 2.58 (5) | .765 | <0.01 | 0.01 | 1.00 | 1.01 | 5475.36 | 0.52 |
| Self-ordered search |  |  |  |  |  |  |  |  |
| 1. Intercept (no growth) | 5.48 (8) | .706 | <0.01 | 0.03 | 1.00 | 1.02 | 7774.86 | 0.69 |
| 2. Linear | 1.13 (5) | .951 | <0.01 | 0.01 | 1.00 | 1.04 | 7776.05 | 0.23 |
| Verbal reasoning |  |  |  |  |  |  |  |  |
| 1. Intercept (no growth) | 142.31 (8) | .000 | 0.20 | 0.12 | 0.84 | 0.88 | 11287.72 | 17.79 |
| 2. Linear | 10.19 (5) | .070 | 0.05 | 0.02 | 0.99 | 0.99 | 11142.71 | 2.04 |
| 3. Quadratic | 0.05 (1) | 0.83 | <0.01 | <0.01 | 1.00 | 1.01 | 11138.61 | 0.05 |

*Note.* χ^2^ = chi-square statistic; SRMR = Standardized Root Mean Squared Residual; RMSEA = root mean square error of approximation; CFI = comparative fit index; TLI = Tucker–Lewis index; AIC = Akaike Information Criterion. All models had freely estimated residual terms (heteroscedastic) and used full information maximum likelihood for missing data with robust maximum likelihood estimation (see Methods for more information).

**Supplementary Table 22. Parameter estimates [95% confidence intervals] for the unconditional latent growth models for each of the cognitive outcomes in individuals aged 62.77 years and over at baseline from the subsample with telomere length data (*n* = 423).**

|  | Paired associates learning | | Digit span test | | Self-ordered search | | Verbal reasoning | | |
| --- | --- | --- | --- | --- | --- | --- | --- | --- | --- |
|  | No growth | Linear | No growth | Linear | No growth | Linear | No growth | Linear | Quadratic |
| Parameter | Estimate | Estimate | Estimate | Estimate | Estimate | Estimate | Estimate | Estimate | Estimate |
| Means | | | | | | | | | |
| Intercept | 4.56 [4.5, 4.62] | 4.5 [4.42, 4.58] | 7.5 [7.38, 7.62] | 7.45 [7.31, 7.59] | 7.41 [7.23, 7.59] | 7.53 [7.31, 7.75] | 35.07 [34.29, 35.85] | 32.83 [32.05, 33.61] | 32.44 [31.60, 33.28] |
| Slope | - | 0.04 [0.00, 0.08] | - | 0.03 [-0.01, 0.07] | - | -0.09 [-0.19, 0.01] | - | 1.36 [1.12, 1.60] | 2.38 [1.64, 3.12] |
| Slope^2^ | - | - | - | - | - | - | - | - | -0.33 [-0.57, -0.09] |
| Variances | | | | | | | | | |
| Intercept | 0.23 [0.17, 0.29] | 0.28 [0.16, 0.40] | 1.28 [1.06, 1.50] | 1.33 [0.96, 1.70] | 2.07 [1.60, 2.54] | 2.32 [1.40, 3.24] | 58.76 [50.12, 67.4] | 51.16 [41.97, 60.35] | 59.29 [40.51, 78.07] |
| Slope | - | 0.02 [0.00, 0.04] | - | 0.05 [-0.01, 0.11] | - | 0.11 [-0.11, 0.33] | - | 0.84 [-0.24, 1.92] | 16.06 [-5.95, 38.07] |
| Slope^2^ | - | - | - | - | - | - | - | - | 1.05 [-0.44, 2.54] |
| Covariances | | | | | | | | | |
| Intercept-Slope | - | -0.03 [-0.07, 0.01] | - | -0.05 [-0.19, 0.09] | - | -0.14 [-0.51, 0.23] | - | 1.84 [-0.67, 4.35] | -7.91 [-27.45, 11.63] |
| Intercept-Slope^2^ | - | - | - | - | - | - | - | - | 2.34 [-2.50, 7.18] |
| Slope-Slope^2^ | - | - | - | - | - | - | - | - | -3.97 [-9.14, 1.20] |
| Residual variances | | | | | | | | | |
| T1 | 0.65 [0.51, 0.79] | 0.57 [0.43, 0.71] | 1.60 [1.11, 2.09] | 1.44 [0.91, 1.97] | 3.86 [2.82, 4.90] | 3.55 [2.33, 4.77] | 38.14 [31.67, 44.61] | 27.49 [21.63, 33.35] | 17.94 [0.52, 35.36] |
| T2 | 0.58 [0.5, 0.66] | 0.57 [0.49, 0.65] | 0.84 [0.64, 1.04] | 0.65 [0.47, 0.83] | 4.27 [3.13, 5.41] | 4.27 [3.11, 5.43] | 21.36 [16.56, 26.16] | 22 [17.49, 26.51] | 22.12 [16.38, 27.86] |
| T3 | 0.50 [0.42, 0.58] | 0.49 [0.41, 0.57] | 0.64 [0.50, 0.78] | 0.65 [0.51, 0.79] | 4.17 [2.99, 5.35] | 4.13 [2.95, 5.31] | 18.17 [13.92, 22.42] | 17.27 [13.11, 21.43] | 14.78 [8.43, 21.13] |
| T4 | 0.51 [0.41, 0.61] | 0.45 [0.35, 0.55] | 0.81 [0.57, 1.05] | 0.67 [0.43, 0.91] | 5.52 [4.13, 6.91] | 5.12 [3.61, 6.63] | 28.26 [23.34, 33.18] | 21.61 [16.02, 27.2] | 21.56 [0.53, 42.59] |

*Note.* All models had freely estimated residual terms (heteroscedastic) and used full information maximum likelihood for missing data with robust maximum likelihood estimation (see Methods for more information).

## Conditional latent growth models

**Supplementary Table 23. Parameter estimates [95% confidence intervals] for the conditional latent growth models for each of the cognitive outcomes (whole sample, *N* = 7,877).**

|  | Paired associated learning | Digit span test | Self-ordered search | Verbal reasoning |
| --- | --- | --- | --- | --- |
|  | Linear | Linear | Linear | Quadratic |
| Parameter | Estimate | Estimate | Estimate | Estimate |
| Means |  |  |  |  |
| Intercept | 6.05 [5.81, 6.29] | 9.15 [8.72, 9.58] | 12.29 [11.65, 12.92] | 47.31 [44.71, 49.91] |
| Slope | 0.01 [-0.10, 0.13] | 0.08 [-0.08, 0.23] | -0.01 [-0.31, 0.29] | 1.78 [-0.68, 4.24] |
| Slope^2^ | - | - | - | 0.20 [-0.58, 0.98] |
| Variances | | | | |
| Intercept | 0.24 [0.20, 0.28] | 1.52 [1.33, 1.71] | 2.08 [1.83, 2.32] | 60.48 [55.07, 65.88] |
| Slope | 0.01 [0.00, 0.02] | 0.03 [-0.00, 0.06] | 0.12 [0.05, 0.18] | 10.66 [4.51, 16.81] |
| Slope^2^ | - | - | - | 0.80 [0.37, 1.24] |
| Covariances | | | | |
| Intercept-Slope | -0.01 [-0.02, 0.01] | -0.06 [-0.11, -0.01] | -0.02 [-0.12, 0.08] | 0.80 [-5.06, 6.66] |
| Intercept-Slope^2^ | - | - | - | 0.04 [-1.42, 1.50] |
| Slope-Slope^2^ | - | - | - | -2.67 [-4.13, -1.22] |
| Residual variances | | | | |
| Baseline | 0.64 [0.59, 0.69] | 1.33 [1.15, 1.51] | 3.68 [3.37, 3.99] | 24.03 [18.84, 29.23] |
| Year 1 | 0.64 [0.60, 0.68] | 1.10 [0.98, 1.23] | 5.43 [5.08, 5.78] | 21.86 [19.93, 23.79] |
| Year 2 | 0.58 [0.55, 0.61] | 1.05 [0.93, 1.16] | 4.31 [3.98, 4.64] | 21.33 [19.32, 23.35] |
| Year 3 | 0.56 [0.51, 0.60] | 0.87 [0.72, 1.02] | 3.76 [3.36, 4.16] | 17.51 [11.36, 23.65] |
| Intercept predictors | | | | |
| PRS-TL | -0.00 [-0.02, 0.02] | -0.02 [-0.05, 0.02] | 0.03 [-0.02, 0.08] | 0.12 [-0.08, 0.32] |
| Age | -0.03 [-0.03, -0.02] | -0.03 [-0.04, -0.02] | -0.07 [-0.08, -0.06] | -0.33 [-0.37, -0.29] |
| Sex | 0.02 [-0.03, 0.06] | -0.14 [-0.22, -0.05] | -0.36 [-0.48, -0.24] | 0.53 [0.06, 1.01] |
| Education | 0.02 [0.01, 0.04] | 0.09 [0.06, 0.11] | 0.10 [0.06, 0.14] | 1.44 [1.29, 1.59] |
| Employment (Full-time vs. Retired) | 0.07 [0.01, 0.13] | 0.19 [0.07, 0.30] | 0.30 [0.13, 0.46] | 0.32 [-0.38, 1.01] |
| Employment (Full-time vs. Part-time | -0.00 [-0.07, 0.06] | 0.10 [-0.02, 0.21] | 0.14 [-0.03, 0.31] | 0.19 [-0.53, 0.90] |
| Employment (Full-time vs. Self-employed) | 0.06 [-0.02, 0.13] | 0.28 [0.15, 0.41] | 0.20 [-0.01, 0.40] | 0.83 [0.01, 1.66] |
| Employment (Full-time vs. Unemployed) | 0.04 [-0.08, 0.15] | 0.04 [-0.18, 0.26] | 0.25 [-0.05, 0.56] | 0.35 [-0.98, 1.68] |
| PC1 | 0.85 [-0.88, 2.58] | 0.68 [-2.50, 3.87] | -10.82 [-15.38, -6.26] | -19.10 [-38.01, -0.19] |
| PC2 | -0.38 [-2.11, 1.34] | -2.33 [-5.43, 0.76] | 5.54 [0.87, 10.21] | 7.64 [-10.25, 25.52] |
| PC3 | -0.09 [-1.87, 1.68] | -0.96 [-4.09, 2.16] | 2.84 [-1.40, 7.08] | -12.27 [-29.97, 5.42] |
| PC4 | -0.19 [-1.89, 1.51] | 0.86 [-2.27, 4.00] | 2.50 [-1.93, 6.93] | -12.42 [-31.19, 6.35] |
| PC5 | 0.50 [-1.20, 2.20] | 0.77 [-2.29, 3.83] | 3.08 [-1.42, 7.59] | -6.68 [-23.49, 10.13] |
| PC6 | 0.99 [-0.63, 2.60] | 0.35 [-2.62, 3.31] | -1.78 [-6.29, 2.73] | -6.90 [-24.66, 10.85] |
| Slope predictors | | | | |
| PRS-TL | -0.00 [-0.01, 0.01] | 0.00 [-0.01, 0.01] | -0.02 [-0.04, 0.00] | -0.13 [-0.33, 0.06] |
| Age | -0.00 [-0.00, 0.00] | -0.00 [-0.00, 0.00] | -0.00 [-0.01, 0.00] | -0.02 [-0.05, 0.02] |
| Sex | 0.02 [-0.00, 0.04] | 0.02 [-0.01, 0.05] | 0.02 [-0.04, 0.08] | 0.90 [0.42, 1.37] |
| Education | 0.01 [-0.00, 0.01] | 0.01 [-0.00, 0.02] | 0.03 [0.02, 0.05] | 0.11 [-0.03, 0.26] |
| Employment (Full-time vs. Retired) | -0.01 [-0.04, 0.02] | 0.01 [-0.03, 0.05] | 0.01 [-0.07, 0.09] | 0.30 [-0.39, 1.00] |
| Employment (Full-time vs. Part-time | 0.01 [-0.02, 0.05] | 0.01 [-0.03, 0.05] | 0.02 [-0.07, 0.10] | -0.08 [-0.79, 0.63] |
| Employment (Full-time vs. Self-employed) | -0.01 [-0.05, 0.02] | 0.01 [-0.04, 0.05] | 0.02 [-0.08, 0.11] | -0.17 [-0.98, 0.63] |
| Employment (Full-time vs. Unemployed) | -0.03 [-0.09, 0.03] | -0.01 [-0.09, 0.06] | -0.07 [-0.22, 0.07] | -0.45 [-1.73, 0.83] |
| PC1 | -0.29 [-1.13, 0.55] | -0.29 [-1.45, 0.88] | 0.55 [-1.59, 2.68] | 1.34 [-16.35, 19.02] |
| PC2 | 0.31 [-0.51, 1.13] | 0.93 [-0.24, 2.10] | -1.63 [-3.77, 0.51] | 5.94 [-10.89, 22.77] |
| PC3 | -0.11 [-0.97, 0.76] | 0.74 [-0.68, 2.17] | -0.14 [-2.32, 2.04] | -0.02 [-14.88, 14.85] |
| PC4 | 0.33 [-0.50, 1.15] | -0.46 [-1.67, 0.76] | -0.06 [-2.14, 2.02] | 3.78 [-13.66, 21.21] |
| PC5 | -0.38 [-1.17, 0.42] | -0.11 [-1.29, 1.06] | 1.33 [-0.94, 3.60] | 4.81 [-12.57, 22.19] |
| PC6 | -0.48 [-1.23, 0.27] | -0.30 [-1.35, 0.76] | 1.17 [-0.84, 3.18] | -6.40 [-22.28, 9.48] |
| Slope^2^ predictors | | | | |
| PRS-TL | - | - | - | 0.04 [-0.02, 0.11] |
| Age | - | - | - | -0.00 [-0.02, 0.01] |
| Sex | - | - | - | -0.14 [-0.29, 0.01] |
| Education | - | - | - | -0.01 [-0.06, 0.03] |
| Employment (Full-time vs. Retired) | - | - | - | -0.08 [-0.30, 0.14] |
| Employment (Full-time vs. Part-time | - | - | - | -0.05 [-0.27, 0.18] |
| Employment (Full-time vs. Self-employed) | - | - | - | 0.04 [-0.21, 0.30] |
| Employment (Full-time vs. Unemployed) | - | - | - | 0.03 [-0.38, 0.45] |
| PC1 | - | - | - | -0.98 [-6.65, 4.69] |
| PC2 | - | - | - | -3.14 [-8.58, 2.30] |
| PC3 | - | - | - | -0.32 [-5.11, 4.47] |
| PC4 | - | - | - | -1.17 [-6.72, 4.38] |
| PC5 | - | - | - | -0.79 [-6.75, 5.16] |
| PC6 | - | - | - | 2.44 [-2.70, 7.58] |

*Note.* PRS-TL = polygenic score for telomere length (leukocyte); PC = genetic principal component*.* PRS-TL was standardised in the whole sample to a mean of 0 and SD of 1. All models had freely estimated residual terms (heteroscedastic) and used full information maximum likelihood for missing data with robust maximum likelihood estimation (see Methods).

**Supplementary Table 24. Parameter estimates [95% confidence intervals] for the conditional latent growth models for each of the cognitive outcomes (relative telomere length subsample, *N* = 846).**

|  | Paired associated learning | | Digit span test | | Self-ordered search | Verbal reasoning |
| --- | --- | --- | --- | --- | --- | --- |
|  | Linear | | Linear | | Linear | Quadratic |
| Parameter | Estimate | | Estimate | | Estimate | Estimate |
| Means |  | |  | |  |  |
| Intercept | 6.58 [5.85, 7.31] | 8.70 [7.44, 9.97] | | 11.71 [9.75, 13.68] | | 43.04 [35.42, 50.66] |
| Slope | -0.02 [-0.35, 0.30] | 0.16 [-0.26, 0.58] | | 0.55 [-0.26, 1.35] | | 6.51 [-0.82, 13.85] |
| Slope^2^ | - | - | | - | | -1.38 [-3.66, 0.90] |
| Variances | | | | | | |
| Intercept | 0.20 [0.12, 0.28] | 1.25 [1.04, 1.45] | | 1.56 [0.99, 2.13] | | 54.25 [40.14, 68.37] |
| Slope | 0.02 [-0.00, 0.03] | 0.03 [-0.00, 0.06] | | 0.06 [-0.09, 0.21] | | 12.02 [-3.66, 27.71] |
| Slope^2^ | - | - | | - | | 1.26 [0.23, 2.28] |
| Covariances | | | | | | |
| Intercept-Slope | -0.02 [-0.05, 0.02] | -0.04 [-0.11, 0.03] | | 0.04 [-0.20, 0.28] | | -2.59 [-17.19, 12.00] |
| Intercept-Slope^2^ | - | - | | - | | 1.17 [-2.48, 4.82] |
| Slope-Slope^2^ | - | - | | - | | -3.61 [-7.27, 0.04] |
| Residual variances | | | | | | |
| Baseline | 0.58 [0.48, 0.68] | 1.08 [0.79, 1.37] | | 3.37 [2.55, 4.20] | | 21.92 [9.06, 34.78] |
| Year 1 | 0.58 [0.51, 0.64] | 0.78 [0.67, 0.89] | | 4.71 [3.82, 5.61] | | 20.16 [16.01, 24.32] |
| Year 2 | 0.53 [0.47, 0.59] | 0.70 [0.60, 0.80] | | 4.02 [3.21, 4.83] | | 19.30 [14.05, 24.54] |
| Year 3 | 0.48 [0.40, 0.56] | 0.76 [0.58, 0.94] | | 4.24 [3.28, 5.19] | | 16.70 [2.82, 30.58] |
| Intercept predictors | | | | | | |
| RTL | 0.00 [-0.05, 0.05] | -0.01 [-0.10, 0.09] | | 0.00 [-0.15, 0.16] | | -0.41 [-0.99, 0.16] |
| Age | -0.03 [-0.04, -0.02] | -0.02 [-0.04, -0.00] | | -0.06 [-0.09, -0.03] | | -0.26 [-0.37, -0.14] |
| Sex | -0.07 [-0.20, 0.06] | -0.21 [-0.43, 0.01] | | -0.49 [-0.86, -0.13] | | 0.94 [-0.45, 2.33] |
| Education | -0.00 [-0.04, 0.04] | 0.14 [0.07, 0.21] | | 0.04 [-0.06, 0.15] | | 1.02 [0.56, 1.48] |
| Employment (Full-time vs. Retired) | 0.10 [-0.07, 0.28] | 0.12 [-0.21, 0.45] | | 0.44 [-0.07, 0.94] | | 2.01 [-0.16, 4.19] |
| Employment (Full-time vs. Part-time | -0.09 [-0.27, 0.10] | 0.06 [-0.27, 0.39] | | 0.44 [-0.04, 0.92] | | 1.76 [-0.34, 3.85] |
| Employment (Full-time vs. Self-employed) | 0.12 [-0.09, 0.32] | 0.41 [0.01, 0.80] | | 0.71 [0.17, 1.25] | | 1.72 [-0.70, 4.14] |
| Employment (Full-time vs. Unemployed) | 0.19 [-0.14, 0.53] | 0.24 [-0.24, 0.73] | | 0.93 [0.24, 1.63] | | 0.09 [-4.18, 4.35] |
| PC1 | 4.85 [0.18, 9.52] | -2.47 [-12.06, 7.12] | | -11.94 [-24.66, 0.78] | | -28.69 [-84.62, 27.24] |
| PC2 | -2.71 [-6.86, 1.44] | -3.72 [-11.67, 4.23] | | 5.60 [-8.70, 19.91] | | -24.85 [-79.75, 30.06] |
| PC3 | -4.78 [-8.97, -0.60] | 1.10 [-5.69, 7.89] | | -3.44 [-16.36, 9.48] | | -11.91 [-69.97, 46.16] |
| PC4 | -1.41 [-5.89, 3.07] | -4.63 [-11.33, 2.07] | | 3.70 [-7.20, 14.61] | | -31.51 [-81.45, 18.44] |
| PC5 | 1.34 [-3.95, 6.63] | -6.98 [-15.82, 1.86] | | 10.22 [-1.63, 22.07] | | 5.78 [-43.28, 54.84] |
| PC6 | -0.16 [-5.46, 5.15] | -1.91 [-10.80, 6.98] | | -2.07 [-16.51, 12.36] | | 14.13 [-42.46, 70.73] |
| Slope predictors | | | | | | |
| RTL | 0.00 [-0.02, 0.02] | -0.01 [-0.04, 0.03] | | -0.01 [-0.07, 0.05] | | -0.19 [-0.76, 0.37] |
| Age | 0.00 [-0.00, 0.01] | -0.00 [-0.01, 0.00] | | -0.01 [-0.02, 0.00] | | -0.03 [-0.14, 0.07] |
| Sex | -0.01 [-0.07, 0.04] | 0.03 [-0.05, 0.10] | | 0.01 [-0.15, 0.17] | | -0.30 [-1.69, 1.09] |
| Education | 0.00 [-0.02, 0.02] | -0.01 [-0.04, 0.02] | | 0.01 [-0.04, 0.05] | | 0.07 [-0.38, 0.52] |
| Employment (Full-time vs. Retired) | -0.00 [-0.08, 0.08] | 0.11 [0.01, 0.21] | | 0.00 [-0.21, 0.22] | | -2.11 [-4.04, -0.17] |
| Employment (Full-time vs. Part-time | 0.04 [-0.04, 0.13] | 0.07 [-0.02, 0.16] | | -0.04 [-0.25, 0.16] | | -0.74 [-2.63, 1.15] |
| Employment (Full-time vs. Self-employed) | -0.03 [-0.12, 0.07] | 0.07 [-0.04, 0.19] | | 0.06 [-0.17, 0.28] | | -1.42 [-3.40, 0.56] |
| Employment (Full-time vs. Unemployed) | -0.09 [-0.23, 0.05] | -0.04 [-0.26, 0.17] | | -0.12 [-0.36, 0.12] | | 1.10 [-1.91, 4.10] |
| PC1 | -2.77 [-4.86, -0.68] | 0.29 [-2.82, 3.40] | | 2.55 [-4.04, 9.15] | | -2.25 [-55.21, 50.71] |
| PC2 | 0.83 [-1.33, 2.99] | 1.95 [-1.17, 5.07] | | 1.07 [-6.31, 8.44] | | -29.09 [-71.65, 13.48] |
| PC3 | 1.59 [-0.93, 4.11] | 0.92 [-2.32, 4.16] | | -1.52 [-8.47, 5.43] | | -18.31 [-56.12, 19.50] |
| PC4 | 0.14 [-1.69, 1.98] | 0.27 [-2.17, 2.71] | | 0.72 [-3.70, 5.14] | | -7.12 [-45.18, 30.94] |
| PC5 | -0.83 [-2.88, 1.21] | 1.90 [-1.16, 4.96] | | -0.80 [-5.94, 4.34] | | -25.88 [-65.15, 13.40] |
| PC6 | -0.59 [-2.78, 1.60] | 1.59 [-1.54, 4.72] | | 0.69 [-6.03, 7.41] | | -13.26 [-63.95, 37.44] |
| Slope^2^ predictors | | | | | | |
| RTL | - | - | | - | | 0.10 [-0.08, 0.27] |
| Age | - | - | | - | | 0.00 [-0.03, 0.03] |
| Sex | - | - | | - | | 0.28 [-0.16, 0.72] |
| Education | - | - | | - | | -0.00 [-0.14, 0.14] |
| Employment (Full-time vs. Retired) | - | - | | - | | 0.65 [0.04, 1.25] |
| Employment (Full-time vs. Part-time | - | - | | - | | 0.20 [-0.41, 0.80] |
| Employment (Full-time vs. Self-employed) | - | - | | - | | 0.57 [-0.06, 1.20] |
| Employment (Full-time vs. Unemployed) | - | - | | - | | -0.42 [-1.39, 0.55] |
| PC1 | - | - | | - | | -0.35 [-16.75, 16.05] |
| PC2 | - | - | | - | | 5.85 [-8.11, 19.81] |
| PC3 | - | - | | - | | 5.22 [-6.38, 16.81] |
| PC4 | - | - | | - | | 2.32 [-10.12, 14.76] |
| PC5 | - | - | | - | | 11.99 [-0.58, 24.56] |
| PC6 | - | - | | - | | 8.64 [-7.14, 24.41] |

*Note.* RTL = RTL (log-transformed, adjusted for batch); PC = genetic principal component*.* RTL was standardised in the whole sample to a mean of 0 and SD of 1. Outliers of +/- 3.29 SDs from the mean (adjusting for covariates) were removed from the relative telomere length variable (see, Methods). All models had freely estimated residual terms (heteroscedastic) and used full information maximum likelihood for missing data with robust maximum likelihood estimation (see Methods).

**Supplementary Table 25. Parameter estimates [95% confidence intervals] for the conditional latent growth models for each of the cognitive outcomes in individuals aged under ~62.24 years at baseline (*N* = 3,938).**

|  | Paired associated learning | Digit span test | Self-ordered search | Verbal reasoning |
| --- | --- | --- | --- | --- |
|  | Linear | Linear | Linear | Quadratic |
| Parameter | Estimate | Estimate | Estimate | Estimate |
| Means |  |  |  |  |
| Intercept | 5.45 [4.97, 5.93] | 7.89 [7.05, 8.73] | 11.61 [10.29, 12.93] | 42.40 [36.97, 47.83] |
| Slope | 0.01 [-0.23, 0.24] | 0.19 [-0.11, 0.50] | 0.32 [-0.29, 0.94] | 0.52 [-4.95, 6.00] |
| Slope^2^ | - | - | - | 0.04 [-1.70, 1.78] |
| Variances | | | | |
| Intercept | 0.27 [0.21, 0.34] | 1.32 [1.14, 1.51] | 2.28 [1.92, 2.65] | 64.92 [56.93, 72.92] |
| Slope | 0.02 [0.01, 0.03] | 0.00^a^ | 0.15 [0.06, 0.24] | 11.43 [2.47, 20.39] |
| Slope^2^ | - | - | - | 1.03 [0.40, 1.66] |
| Covariances | | | | |
| Intercept-Slope | -0.03 [-0.05, -0.00] | -0.01 [-0.04, 0.02] | -0.05 [-0.20, 0.10] | 1.84 [-6.74, 10.42] |
| Intercept-Slope^2^ | - | - | - | -0.14 [-2.28, 1.99] |
| Slope-Slope^2^ | - | - | - | -3.11 [-5.21, -1.00] |
| Residual variances | | | | |
| Baseline | 0.59 [0.53, 0.66] | 1.26 [1.06, 1.46] | 3.40 [2.96, 3.84] | 25.21 [17.55, 32.88] |
| Year 1 | 0.61 [0.56, 0.65] | 0.88 [0.78, 0.97] | 5.76 [5.22, 6.31] | 22.79 [19.91, 25.66] |
| Year 2 | 0.57 [0.52, 0.61] | 0.92 [0.80, 1.04] | 4.34 [3.86, 4.83] | 22.85 [19.88, 25.82] |
| Year 3 | 0.55 [0.49, 0.61] | 0.87 [0.71, 1.02] | 3.33 [2.76, 3.90] | 16.69 [7.53, 25.86] |
| Intercept predictors | | | | |
| PRS-TL | -0.01 [-0.04, 0.01] | -0.02 [-0.07, 0.03] | 0.03 [-0.04, 0.10] | 0.13 [-0.17, 0.42] |
| Age | -0.02 [-0.02, -0.01] | -0.01 [-0.02, 0.01] | -0.06 [-0.08, -0.04] | -0.25 [-0.34, -0.16] |
| Sex | 0.01 [-0.06, 0.08] | -0.15 [-0.27, -0.03] | -0.39 [-0.58, -0.20] | 0.56 [-0.20, 1.31] |
| Education | 0.03 [0.01, 0.05] | 0.10 [0.06, 0.13] | 0.12 [0.06, 0.17] | 1.47 [1.24, 1.70] |
| Employment (Full-time vs. Retired) | 0.10 [0.02, 0.18] | 0.24 [0.10, 0.38] | 0.33 [0.12, 0.54] | 0.23 [-0.66, 1.12] |
| Employment (Full-time vs. Part-time | 0.00 [-0.07, 0.07] | 0.08 [-0.04, 0.20] | 0.15 [-0.04, 0.33] | 0.12 [-0.69, 0.92] |
| Employment (Full-time vs. Self-employed) | 0.04 [-0.04, 0.13] | 0.19 [0.05, 0.34] | 0.16 [-0.08, 0.39] | 0.71 [-0.24, 1.66] |
| Employment (Full-time vs. Unemployed) | 0.05 [-0.07, 0.17] | 0.06 [-0.16, 0.29] | 0.26 [-0.06, 0.57] | 0.71 [-0.66, 2.08] |
| PC1 | 1.05 [-1.31, 3.40] | 1.91 [-2.11, 5.92] | -12.15 [-18.62, -5.68] | -22.96 [-49.65, 3.73] |
| PC2 | -1.68 [-3.80, 0.44] | -4.35 [-8.56, -0.15] | 6.43 [-0.36, 13.21] | -3.01 [-28.23, 22.20] |
| PC3 | -1.87 [-3.83, 0.09] | -1.69 [-5.98, 2.59] | 3.33 [-2.41, 9.07] | -11.69 [-35.49, 12.10] |
| PC4 | -0.83 [-3.26, 1.59] | -0.49 [-4.52, 3.54] | -1.26 [-7.42, 4.91] | -0.18 [-28.02, 27.66] |
| PC5 | -0.54 [-2.64, 1.56] | -0.07 [-3.44, 3.30] | 0.86 [-5.09, 6.81] | 1.90 [-21.79, 25.59] |
| PC6 | 1.15 [-1.04, 3.34] | 1.54 [-2.52, 5.59] | -3.06 [-9.33, 3.22] | 3.92 [-22.00, 29.84] |
| Slope predictors | | | | |
| PRS-TL | 0.00 [-0.01, 0.02] | 0.01 [-0.01, 0.03] | -0.02 [-0.05, 0.02] | 0.19 [-0.10, 0.48] |
| Age | 0.00 [-0.00, 0.00] | -0.00 [-0.01, 0.00] | -0.01 [-0.02, 0.00] | 0.01 [-0.08, 0.10] |
| Sex | 0.01 [-0.02, 0.04] | -0.01 [-0.05, 0.03] | -0.08 [-0.17, 0.01] | 1.02 [0.26, 1.78] |
| Education | 0.01 [-0.00, 0.02] | 0.01 [-0.00, 0.02] | 0.04 [0.01, 0.06] | -0.03 [-0.24, 0.19] |
| Employment (Full-time vs. Retired) | -0.02 [-0.06, 0.02] | 0.01 [-0.04, 0.06] | 0.02 [-0.08, 0.12] | 0.33 [-0.55, 1.22] |
| Employment (Full-time vs. Part-time | 0.01 [-0.03, 0.04] | 0.03 [-0.01, 0.07] | 0.01 [-0.08, 0.10] | -0.07 [-0.86, 0.72] |
| Employment (Full-time vs. Self-employed) | 0.01 [-0.03, 0.05] | 0.03 [-0.02, 0.09] | 0.02 [-0.08, 0.13] | -0.22 [-1.14, 0.70] |
| Employment (Full-time vs. Unemployed) | -0.03 [-0.09, 0.03] | -0.01 [-0.09, 0.06] | -0.05 [-0.20, 0.10] | -0.45 [-1.77, 0.87] |
| PC1 | -0.15 [-1.29, 1.00] | -0.57 [-2.05, 0.91] | 1.26 [-1.63, 4.15] | 3.95 [-20.74, 28.65] |
| PC2 | 1.48 [0.42, 2.53] | 1.27 [-0.28, 2.81] | -1.29 [-4.08, 1.50] | 4.44 [-19.29, 28.17] |
| PC3 | 0.02 [-1.12, 1.15] | 0.16 [-1.82, 2.13] | -1.75 [-4.37, 0.87] | -2.76 [-22.26, 16.74] |
| PC4 | -0.05 [-1.22, 1.13] | -0.54 [-1.96, 0.87] | 1.29 [-1.52, 4.11] | 5.39 [-19.44, 30.23] |
| PC5 | -0.53 [-1.54, 0.49] | -0.00 [-1.30, 1.29] | 0.63 [-2.15, 3.41] | 2.81 [-20.32, 25.93] |
| PC6 | -0.45 [-1.48, 0.58] | -0.65 [-2.09, 0.79] | 0.91 [-1.97, 3.79] | -2.67 [-25.55, 20.21] |
| Slope^2^ predictors | | | | |
| PRS-TL | - | - | - | -0.05 [-0.14, 0.04] |
| Age | - | - | - | -0.01 [-0.03, 0.02] |
| Sex | - | - | - | -0.14 [-0.39, 0.10] |
| Education | - | - | - | 0.04 [-0.03, 0.11] |
| Employment (Full-time vs. Retired) | - | - | - | -0.08 [-0.36, 0.20] |
| Employment (Full-time vs. Part-time | - | - | - | -0.05 [-0.31, 0.20] |
| Employment (Full-time vs. Self-employed) | - | - | - | 0.06 [-0.23, 0.35] |
| Employment (Full-time vs. Unemployed) | - | - | - | 0.05 [-0.38, 0.48] |
| PC1 | - | - | - | -3.16 [-10.97, 4.65] |
| PC2 | - | - | - | -3.68 [-11.01, 3.65] |
| PC3 | - | - | - | 1.16 [-4.93, 7.25] |
| PC4 | - | - | - | -2.14 [-10.07, 5.79] |
| PC5 | - | - | - | 0.90 [-6.95, 8.75] |
| PC6 | - | - | - | 0.72 [-6.67, 8.12] |

*Note.* PRS-TL = polygenic score for telomere length (leukocyte); PC = genetic principal component*.* PRS-TL was standardised in the whole sample to a mean of 0 and SD of 1. All models had freely estimated residual terms (heteroscedastic) and used full information maximum likelihood for missing data with robust maximum likelihood estimation (see Methods).

^a^ For the digit span test model, the variance for the slope variance was fixed to 0.0001 (i.e., estimated as a fixed effect; see Supplementary Methods S2), hence no confidence intervals are provided.

**Supplementary Table 26. Parameter estimates [95% confidence intervals] for the conditional latent growth models for each of the cognitive outcomes in individuals aged 62.24 years and over at baseline (*N* = 3,939).**

|  | Paired associated learning | Digit span test | Self-ordered search | Verbal reasoning |
| --- | --- | --- | --- | --- |
|  | Linear | Linear | Linear | Quadratic |
| Parameter | Estimate | Estimate | Estimate | Estimate |
| Means |  |  |  |  |
| Intercept | 6.32 [5.87, 6.78] | 10.06 [9.26, 10.86] | 13.35 [12.16, 14.55] | 54.72 [49.77, 59.67] |
| Slope | 0.09 [-0.12, 0.30] | 0.11 [-0.20, 0.42] | -0.28 [-0.86, 0.29] | 3.23 [-1.13, 7.60] |
| Slope^2^ | - | - | - | 0.21 [-1.15, 1.58] |
| Variances | | | | |
| Intercept | 0.19 [0.15, 0.24] | 1.70 [1.39, 2.01] | 1.85 [1.52, 2.18] | 55.55 [48.28, 62.81] |
| Slope | 0.00 [-0.01, 0.01] | 0.06 [0.02, 0.10] | 0.08 [-0.01, 0.17] | 9.67 [1.22, 18.12] |
| Slope^2^ | - | - | - | 0.55 [-0.05, 1.14] |
| Covariances | | | | |
| Intercept-Slope | 0.01 [-0.01, 0.03] | -0.12 [-0.20, -0.03] | 0.02 [-0.13, 0.16] | -0.44 [-8.42, 7.55] |
| Intercept-Slope^2^ | - | - | - | 0.25 [-1.74, 2.24] |
| Slope-Slope^2^ | - | - | - | -2.17 [-4.17, -0.17] |
| Residual variances | | | | |
| Baseline | 0.70 [0.62, 0.77] | 1.38 [1.13, 1.63] | 3.96 [3.53, 4.39] | 22.79 [15.78, 29.81] |
| Year 1 | 0.68 [0.62, 0.73] | 1.33 [1.09, 1.56] | 5.10 [4.65, 5.56] | 20.89 [18.32, 23.46] |
| Year 2 | 0.59 [0.55, 0.64] | 1.17 [0.98, 1.36] | 4.27 [3.82, 4.71] | 19.89 [17.19, 22.59] |
| Year 3 | 0.56 [0.50, 0.63] | 0.86 [0.67, 1.05] | 4.19 [3.63, 4.74] | 18.45 [10.38, 26.53] |
| Intercept predictors | | | | |
| PRS-TL | 0.01 [-0.02, 0.03] | -0.01 [-0.06, 0.04] | 0.02 [-0.05, 0.09] | 0.11 [-0.17, 0.39] |
| Age | -0.03 [-0.03, -0.02] | -0.04 [-0.05, -0.03] | -0.09 [-0.10, -0.07] | -0.42 [-0.49, -0.36] |
| Sex | 0.02 [-0.04, 0.08] | -0.13 [-0.24, -0.02] | -0.33 [-0.49, -0.18] | 0.47 [-0.15, 1.08] |
| Education | 0.02 [-0.00, 0.04] | 0.08 [0.04, 0.11] | 0.08 [0.04, 0.13] | 1.40 [1.20, 1.59] |
| Employment (Full-time vs. Retired) | -0.04 [-0.18, 0.10] | 0.05 [-0.22, 0.31] | 0.11 [-0.30, 0.51] | -0.65 [-2.20, 0.90] |
| Employment (Full-time vs. Part-time | -0.10 [-0.27, 0.06] | 0.02 [-0.29, 0.34] | -0.05 [-0.51, 0.41] | -0.51 [-2.29, 1.26] |
| Employment (Full-time vs. Self-employed) | 0.00 [-0.17, 0.17] | 0.34 [0.02, 0.66] | 0.13 [-0.36, 0.62] | 0.23 [-1.65, 2.11] |
| Employment (Full-time vs. Unemployed) | -0.08 [-0.60, 0.43] | -0.38 [-1.36, 0.61] | 0.76 [-0.46, 1.98] | -5.70 [-10.83, -0.58] |
| PC1 | 0.34 [-2.20, 2.89] | -1.33 [-6.35, 3.69] | -9.61 [-15.98, -3.24] | -16.19 [-42.91, 10.54] |
| PC2 | 0.85 [-1.94, 3.64] | -0.26 [-4.86, 4.34] | 5.45 [-0.75, 11.65] | 18.64 [-6.76, 44.05] |
| PC3 | 2.16 [-1.12, 5.44] | -0.17 [-4.65, 4.31] | 1.62 [-4.46, 7.71] | -14.01 [-40.88, 12.85] |
| PC4 | 0.70 [-1.67, 3.07] | 2.36 [-2.48, 7.20] | 6.46 [0.16, 12.77] | -24.06 [-49.59, 1.47] |
| PC5 | 1.75 [-0.89, 4.38] | 2.00 [-3.25, 7.24] | 5.92 [-0.67, 12.51] | -16.20 [-40.13, 7.72] |
| PC6 | 0.46 [-1.96, 2.88] | -1.18 [-5.62, 3.25] | -0.62 [-6.81, 5.57] | -16.09 [-41.13, 8.96] |
| Slope predictors | | | | |
| PRS-TL | -0.00 [-0.02, 0.01] | -0.01 [-0.02, 0.01] | -0.03 [-0.06, 0.00] | -0.45 [-0.72, -0.18] |
| Age | -0.00 [-0.00, 0.00] | -0.00 [-0.01, 0.00] | -0.00 [-0.01, 0.01] | -0.03 [-0.09, 0.02] |
| Sex | 0.02 [-0.01, 0.05] | 0.03 [-0.01, 0.08] | 0.09 [0.01, 0.17] | 0.80 [0.20, 1.40] |
| Education | 0.00 [-0.00, 0.01] | 0.00 [-0.01, 0.02] | 0.04 [0.01, 0.06] | 0.21 [0.02, 0.41] |
| Employment (Full-time vs. Retired) | -0.01 [-0.08, 0.07] | -0.05 [-0.15, 0.05] | 0.04 [-0.18, 0.25] | -0.20 [-1.86, 1.45] |
| Employment (Full-time vs. Part-time | 0.05 [-0.04, 0.13] | -0.08 [-0.19, 0.04] | 0.12 [-0.12, 0.36] | -0.63 [-2.52, 1.27] |
| Employment (Full-time vs. Self-employed) | -0.05 [-0.14, 0.04] | -0.09 [-0.21, 0.03] | 0.04 [-0.23, 0.30] | -0.47 [-2.42, 1.47] |
| Employment (Full-time vs. Unemployed) | 0.04 [-0.25, 0.33] | 0.31 [-0.10, 0.73] | -0.27 [-0.94, 0.39] | -2.02 [-7.63, 3.59] |
| PC1 | -0.49 [-1.72, 0.75] | 0.14 [-1.69, 1.96] | -0.52 [-3.69, 2.64] | -0.46 [-25.93, 25.01] |
| PC2 | -0.96 [-2.17, 0.25] | 0.58 [-1.26, 2.42] | -2.27 [-5.49, 0.95] | 7.45 [-16.79, 31.68] |
| PC3 | 0.04 [-1.19, 1.27] | 1.80 [-0.07, 3.68] | 2.49 [-1.03, 6.01] | 6.28 [-16.23, 28.80] |
| PC4 | 0.69 [-0.49, 1.86] | -0.38 [-2.39, 1.63] | -1.46 [-4.47, 1.55] | 0.32 [-24.18, 24.82] |
| PC5 | -0.19 [-1.44, 1.05] | -0.37 [-2.46, 1.73] | 2.07 [-1.52, 5.65] | 8.82 [-16.87, 34.50] |
| PC6 | -0.78 [-1.91, 0.35] | -0.02 [-1.73, 1.69] | 1.04 [-1.83, 3.91] | -9.18 [-31.61, 13.25] |
| Slope^2^ predictors | | | | |
| PRS-TL | - | - | - | 0.13 [0.05, 0.22] |
| Age | - | - | - | -0.00 [-0.02, 0.01] |
| Sex | - | - | - | -0.14 [-0.33, 0.05] |
| Education | - | - | - | -0.05 [-0.11, 0.01] |
| Employment (Full-time vs. Retired) | - | - | - | -0.04 [-0.55, 0.47] |
| Employment (Full-time vs. Part-time | - | - | - | 0.02 [-0.56, 0.61] |
| Employment (Full-time vs. Self-employed) | - | - | - | 0.03 [-0.57, 0.63] |
| Employment (Full-time vs. Unemployed) | - | - | - | 0.22 [-1.39, 1.84] |
| PC1 | - | - | - | 1.27 [-7.01, 9.55] |
| PC2 | - | - | - | -2.26 [-10.27, 5.75] |
| PC3 | - | - | - | -3.54 [-11.00, 3.93] |
| PC4 | - | - | - | 0.23 [-7.54, 8.01] |
| PC5 | - | - | - | -3.64 [-11.72, 4.45] |
| PC6 | - | - | - | 4.41 [-2.54, 11.36] |

*Note.* PRS-TL = polygenic score for telomere length (leukocyte); PC = genetic principal component*.* PRS-TL was standardised in the whole sample to a mean of 0 and SD of 1. All models had freely estimated residual terms (heteroscedastic) and used full information maximum likelihood for missing data with robust maximum likelihood estimation (see Methods).

**Supplementary Table 27. Parameter estimates [95% confidence intervals] for the conditional latent growth models for each of the cognitive outcomes in individuals aged under ~62.77 years at baseline (RTL subsample, *n* = 423).**

|  | Paired associated learning | Digit span test | Self-ordered search | Verbal reasoning |
| --- | --- | --- | --- | --- |
|  | Linear | Linear | Linear | Quadratic |
| Parameter | Estimate | Estimate | Estimate | Estimate |
| Means |  |  |  |  |
| Intercept | 6.54 [5.26, 7.83] | 8.73 [6.55, 10.90] | 10.19 [6.53, 13.85] | 34.20 [18.86, 49.53] |
| Slope | -0.01 [-0.62, 0.60] | -0.22 [-0.94, 0.49] | 1.38 [-0.09, 2.85] | 10.01 [-2.15, 22.16] |
| Slope^2^ | - | - | - | -2.62 [-6.47, 1.23] |
| Variances | | | | |
| Intercept | 0.12 [0.01, 0.23] | 1.22 [0.98, 1.46] | 1.11 [0.39, 1.83] | 52.09 [31.02, 73.16] |
| Slope | 0.01 [-0.02, 0.03] | 0.01 [-0.02, 0.05] | 0.04 [-0.16, 0.24] | 6.90 [-15.63, 29.43] |
| Slope^2^ | - | - | - | 1.60 [0.18, 3.02] |
| Covariances | | | | |
| Intercept-Slope | 0.01 [-0.04, 0.05] | -0.03 [-0.10, 0.03] | 0.18 [-0.12, 0.48] | 5.59 [-16.13, 27.30] |
| Intercept-Slope^2^ | - | - | - | -0.60 [-6.09, 4.89] |
| Slope-Slope^2^ | - | - | - | -3.24 [-8.46, 1.98] |
| Residual variances | | | | |
| Baseline | 0.61 [0.46, 0.76] | 0.73 [0.48, 0.98] | 3.00 [1.92, 4.07] | 27.75 [8.86, 46.64] |
| Year 1 | 0.57 [0.47, 0.67] | 0.74 [0.61, 0.87] | 5.26 [3.87, 6.66] | 17.97 [11.78, 24.15] |
| Year 2 | 0.57 [0.48, 0.66] | 0.76 [0.60, 0.91] | 3.97 [2.83, 5.10] | 24.18 [15.51, 32.85] |
| Year 3 | 0.52 [0.39, 0.66] | 0.83 [0.55, 1.11] | 3.28 [2.08, 4.48] | 8.86 [-9.93, 27.65] |
| Intercept predictors | | | | |
| RTL | -0.02 [-0.09, 0.05] | 0.06 [-0.07, 0.18] | -0.08 [-0.30, 0.15] | 0.22 [-0.59, 1.03] |
| Age | -0.02 [-0.05, -0.00] | -0.01 [-0.05, 0.02] | -0.03 [-0.09, 0.03] | -0.07 [-0.33, 0.18] |
| Sex | -0.22 [-0.42, -0.02] | -0.48 [-0.81, -0.16] | -0.39 [-0.92, 0.13] | 0.01 [-2.18, 2.19] |
| Education | -0.01 [-0.07, 0.05] | 0.09 [-0.01, 0.18] | 0.03 [-0.11, 0.16] | 1.00 [0.32, 1.69] |
| Employment (Full-time vs. Not working) | 0.15 [-0.05, 0.36] | 0.25 [-0.09, 0.60] | 0.56 [0.01, 1.12] | 1.85 [-0.69, 4.39] |
| Employment (Full-time vs. Part-time | -0.11 [-0.31, 0.09] | 0.10 [-0.25, 0.45] | 0.35 [-0.18, 0.87] | 2.00 [-0.31, 4.31] |
| Employment (Full-time vs. Self-employed) | 0.08 [-0.13, 0.30] | 0.29 [-0.11, 0.70] | 0.83 [0.25, 1.40] | 1.69 [-1.10, 4.49] |
| PC1 | 5.34 [-1.10, 11.77] | 0.23 [-12.48, 12.94] | -10.11 [-26.99, 6.77] | -31.03 [-105.53, 43.47] |
| PC2 | -3.66 [-8.56, 1.25] | -7.52 [-18.13, 3.09] | 8.24 [-11.51, 28.00] | -5.92 [-82.06, 70.23] |
| PC3 | -6.85 [-12.19, -1.50] | -0.71 [-8.16, 6.74] | 5.58 [-5.15, 16.30] | 26.88 [-40.67, 94.42] |
| PC4 | -3.49 [-8.52, 1.54] | -3.42 [-11.20, 4.35] | 1.90 [-12.27, 16.07] | -35.94 [-99.64, 27.76] |
| PC5 | 3.73 [-3.53, 10.99] | -4.43 [-14.91, 6.05] | 12.14 [-4.93, 29.21] | -2.16 [-84.94, 80.63] |
| PC6 | 3.35 [-3.50, 10.20] | -2.15 [-13.50, 9.20] | -10.06 [-28.66, 8.55] | 35.41 [-48.27, 119.09] |
| Slope predictors | | | | |
| RTL | 0.01 [-0.02, 0.04] | -0.02 [-0.06, 0.02] | 0.02 [-0.07, 0.11] | -0.64 [-1.48, 0.21] |
| Age | -0.00 [-0.01, 0.01] | 0.00 [-0.01, 0.01] | -0.02 [-0.05, 0.00] | -0.09 [-0.30, 0.11] |
| Sex | 0.01 [-0.08, 0.10] | 0.04 [-0.07, 0.15] | -0.15 [-0.37, 0.08] | 0.06 [-2.41, 2.53] |
| Education | 0.00 [-0.02, 0.03] | 0.00 [-0.03, 0.03] | 0.02 [-0.05, 0.08] | -0.05 [-0.71, 0.61] |
| Employment (Full-time vs. Not working) | 0.01 [-0.09, 0.10] | 0.07 [-0.05, 0.19] | -0.01 [-0.25, 0.24] | -1.03 [-3.33, 1.27] |
| Employment (Full-time vs. Part-time | 0.05 [-0.04, 0.15] | 0.09 [-0.01, 0.20] | -0.03 [-0.26, 0.20] | -1.45 [-3.55, 0.65] |
| Employment (Full-time vs. Self-employed) | 0.00 [-0.11, 0.11] | 0.13 [0.01, 0.25] | -0.00 [-0.24, 0.23] | -2.20 [-4.37, -0.03] |
| PC1 | -3.67 [-6.67, -0.68] | 0.81 [-2.90, 4.51] | 3.10 [-5.40, 11.59] | -9.86 [-77.81, 58.09] |
| PC2 | 0.87 [-1.80, 3.53] | 0.74 [-3.99, 5.47] | -0.30 [-9.99, 9.39] | -13.78 [-72.93, 45.38] |
| PC3 | 2.70 [-0.57, 5.97] | 0.35 [-3.11, 3.81] | -3.35 [-10.26, 3.55] | -36.00 [-81.29, 9.30] |
| PC4 | 1.43 [-0.98, 3.85] | 0.02 [-2.71, 2.74] | 0.43 [-5.35, 6.20] | 12.17 [-34.69, 59.03] |
| PC5 | -2.78 [-5.78, 0.21] | 0.52 [-3.12, 4.16] | -4.21 [-12.33, 3.91] | -12.58 [-76.92, 51.77] |
| PC6 | -1.08 [-4.05, 1.90] | 0.91 [-2.89, 4.70] | 3.95 [-5.35, 13.26] | -5.74 [-78.46, 66.98] |
| Slope^2^ predictors | | | | |
| RTL | - | - | - | 0.19 [-0.08, 0.46] |
| Age | - | - | - | 0.02 [-0.04, 0.09] |
| Sex | - | - | - | 0.12 [-0.68, 0.92] |
| Education | - | - | - | 0.04 [-0.17, 0.26] |
| Employment (Full-time vs. Not working) | - | - | - | 0.40 [-0.33, 1.13] |
| Employment (Full-time vs. Part-time | - | - | - | 0.43 [-0.25, 1.11] |
| Employment (Full-time vs. Self-employed) | - | - | - | 0.73 [0.03, 1.43] |
| PC1 | - | - | - | -2.37 [-24.18, 19.44] |
| PC2 | - | - | - | 0.62 [-19.23, 20.47] |
| PC3 | - | - | - | 11.24 [-2.39, 24.86] |
| PC4 | - | - | - | -3.34 [-19.28, 12.61] |
| PC5 | - | - | - | 11.24 [-9.42, 31.91] |
| PC6 | - | - | - | 6.43 [-16.77, 29.62] |

*Note.* RTL = RTL (log-transformed, adjusted for batch); PC = genetic principal component*.* RTL was standardised in the whole sample to a mean of 0 and SD of 1. Outliers of +/- 3.29 SDs from the mean (adjusting for covariates) were removed from the relative telomere length variable (see, Methods). All models had freely estimated residual terms (heteroscedastic) and used full information maximum likelihood for missing data with robust maximum likelihood estimation (see Methods).

**Supplementary Table 28. Parameter estimates [95% confidence intervals] for the conditional latent growth models for each of the cognitive outcomes in individuals aged 62.77 years and over at baseline (RTL subsample, *n* = 423).**

|  | Paired associated learning | Digit span test | Self-ordered search | Verbal reasoning |
| --- | --- | --- | --- | --- |
|  | Linear | Linear | Linear | Quadratic |
| Parameter | Estimate | Estimate | Estimate | Estimate |
| Means |  |  |  |  |
| Intercept | 7.50 [6.22, 8.79] | 9.01 [6.45, 11.57] | 14.07 [9.90, 18.25] | 47.58 [33.02, 62.14] |
| Slope | -0.02 [-0.59, 0.55] | 0.41 [-0.49, 1.31] | 0.59 [-1.13, 2.31] | 6.19 [-7.21, 19.60] |
| Slope^2^ | - | - | - | -0.64 [-4.76, 3.49] |
| Variances | | | | |
| Intercept | 0.24 [0.13, 0.34] | 1.18 [0.87, 1.50] | 1.93 [1.04, 2.83] | 50.72 [32.33, 69.11] |
| Slope | 0.02 [-0.00, 0.04] | 0.04 [-0.01, 0.09] | 0.07 [-0.14, 0.28] | 12.04 [-9.60, 33.67] |
| Slope^2^ | - | - | - | 0.77 [-0.66, 2.20] |
| Covariances | | | | |
| Intercept-Slope | -0.03 [-0.07, 0.01] | -0.04 [-0.16, 0.08] | -0.12 [-0.48, 0.25] | -6.15 [-25.60, 13.31] |
| Intercept-Slope^2^ | - | - | - | 1.78 [-3.05, 6.61] |
| Slope-Slope^2^ | - | - | - | -2.99 [-8.05, 2.08] |
| Residual variances | | | | |
| Baseline | 0.55 [0.42, 0.69] | 1.44 [0.93, 1.95] | 3.65 [2.45, 4.85] | 19.87 [2.32, 37.43] |
| Year 1 | 0.58 [0.49, 0.66] | 0.82 [0.65, 0.99] | 4.17 [3.05, 5.29] | 21.56 [15.84, 27.28] |
| Year 2 | 0.50 [0.43, 0.58] | 0.65 [0.52, 0.79] | 4.12 [2.96, 5.29] | 15.08 [8.79, 21.37] |
| Year 3 | 0.44 [0.34, 0.55] | 0.65 [0.43, 0.87] | 5.26 [3.75, 6.77] | 21.95 [2.13, 41.78] |
| Intercept predictors | | | | |
| RTL | 0.03 [-0.04, 0.11] | -0.08 [-0.22, 0.06] | 0.10 [-0.11, 0.30] | -1.05 [-1.85, -0.24] |
| Age | -0.04 [-0.06, -0.03] | -0.03 [-0.07, 0.00] | -0.09 [-0.14, -0.04] | -0.32 [-0.50, -0.13] |
| Sex | 0.04 [-0.12, 0.21] | -0.00 [-0.29, 0.29] | -0.48 [-0.97, 0.00] | 1.36 [-0.41, 3.14] |
| Education | -0.00 [-0.06, 0.06] | 0.20 [0.10, 0.31] | 0.08 [-0.07, 0.23] | 1.14 [0.52, 1.76] |
| Employment (Full-time vs. Not working) | -0.05 [-0.34, 0.25] | -0.14 [-0.96, 0.67] | 0.03 [-1.16, 1.22] | 0.36 [-4.95, 5.68] |
| Employment (Full-time vs. Part-time | -0.05 [-0.47, 0.37] | -0.09 [-1.02, 0.84] | 0.33 [-0.96, 1.62] | -0.18 [-6.12, 5.75] |
| Employment (Full-time vs. Self-employed) | 0.09 [-0.36, 0.54] | 0.56 [-0.48, 1.61] | 0.10 [-1.32, 1.53] | 0.95 [-4.91, 6.80] |
| PC1 | 3.15 [-3.85, 10.15] | -6.44 [-20.42, 7.53] | -15.27 [-33.38, 2.83] | -28.24 [-112.36, 55.88] |
| PC2 | -2.33 [-9.16, 4.50] | 0.79 [-10.28, 11.87] | 8.11 [-9.69, 25.91] | -26.99 [-109.83, 55.86] |
| PC3 | 1.72 [-5.49, 8.93] | -2.57 [-17.23, 12.09] | -26.59 [-53.40, 0.22] | -98.27 [-209.39, 12.85] |
| PC4 | 1.53 [-5.56, 8.63] | -7.65 [-19.02, 3.72] | 3.52 [-14.21, 21.25] | -34.52 [-109.85, 40.81] |
| PC5 | 0.19 [-7.32, 7.71] | -9.11 [-21.53, 3.31] | 7.05 [-10.24, 24.35] | -3.93 [-62.08, 54.22] |
| PC6 | -3.73 [-11.62, 4.15] | -0.77 [-14.17, 12.64] | 6.17 [-16.50, 28.85] | -9.76 [-85.05, 65.53] |
| Slope predictors | | | | |
| RTL | -0.01 [-0.04, 0.03] | 0.01 [-0.04, 0.07] | -0.04 [-0.12, 0.05] | 0.30 [-0.46, 1.06] |
| Age | 0.00 [-0.01, 0.01] | -0.00 [-0.02, 0.01] | -0.01 [-0.03, 0.01] | -0.04 [-0.22, 0.13] |
| Sex | -0.02 [-0.09, 0.05] | 0.01 [-0.09, 0.10] | 0.10 [-0.11, 0.32] | -0.22 [-1.92, 1.49] |
| Education | 0.00 [-0.02, 0.03] | -0.03 [-0.07, 0.01] | -0.01 [-0.08, 0.06] | 0.14 [-0.49, 0.77] |
| Employment (Full-time vs. Not working) | -0.09 [-0.25, 0.08] | 0.05 [-0.12, 0.22] | 0.04 [-0.46, 0.54] | -1.51 [-5.28, 2.25] |
| Employment (Full-time vs. Part-time | -0.04 [-0.24, 0.16] | -0.03 [-0.23, 0.17] | 0.06 [-0.48, 0.60] | 1.33 [-3.47, 6.13] |
| Employment (Full-time vs. Self-employed) | -0.15 [-0.36, 0.06] | -0.09 [-0.37, 0.19] | 0.23 [-0.41, 0.87] | 0.78 [-3.90, 5.46] |
| PC1 | -1.46 [-4.44, 1.53] | -0.15 [-5.23, 4.92] | 1.16 [-8.79, 11.12] | -2.38 [-84.59, 79.84] |
| PC2 | 1.25 [-2.23, 4.72] | 2.53 [-2.37, 7.42] | 2.18 [-8.76, 13.13] | -60.08 [-119.17, -1.00] |
| PC3 | -1.16 [-4.74, 2.42] | 5.45 [-1.93, 12.83] | 4.11 [-11.37, 19.58] | 35.77 [-37.75, 109.29] |
| PC4 | -1.64 [-4.64, 1.35] | 1.21 [-2.89, 5.31] | 1.78 [-5.44, 8.99] | -24.40 [-88.17, 39.38] |
| PC5 | 0.13 [-2.47, 2.74] | 3.25 [-1.10, 7.60] | 2.57 [-5.08, 10.22] | -31.02 [-82.58, 20.54] |
| PC6 | -0.02 [-3.19, 3.16] | 1.71 [-3.19, 6.60] | -2.84 [-12.67, 6.99] | -5.09 [-76.92, 66.75] |
| Slope^2^ predictors | | | | |
| RTL | - | - | - | -0.01 [-0.24, 0.22] |
| Age | - | - | - | -0.00 [-0.06, 0.05] |
| Sex | - | - | - | 0.31 [-0.22, 0.84] |
| Education | - | - | - | -0.03 [-0.22, 0.16] |
| Employment (Full-time vs. Not working) | - | - | - | 0.19 [-1.03, 1.40] |
| Employment (Full-time vs. Part-time | - | - | - | -0.64 [-2.15, 0.86] |
| Employment (Full-time vs. Self-employed) | - | - | - | -0.09 [-1.56, 1.38] |
| PC1 | - | - | - | 4.53 [-20.31, 29.37] |
| PC2 | - | - | - | 16.85 [-1.29, 34.99] |
| PC3 | - | - | - | -14.14 [-35.61, 7.33] |
| PC4 | - | - | - | 6.84 [-13.18, 26.86] |
| PC5 | - | - | - | 10.31 [-5.77, 26.39] |
| PC6 | - | - | - | 6.83 [-14.83, 28.50] |

*Note.* RTL = RTL (log-transformed, adjusted for batch); PC = genetic principal component*.* RTL was standardised in the whole sample to a mean of 0 and SD of 1. Outliers of +/- 3.29 SDs from the mean (adjusting for covariates) were removed from the relative telomere length variable (see, Methods). All models had freely estimated residual terms (heteroscedastic) and used full information maximum likelihood for missing data with robust maximum likelihood estimation (see Methods).

**Supplementary Table 29. Fit indices for the conditional latent growth models for each of the cognitive outcomes.**

| Model (growth shape) | χ^2^(*df*) | *p*-value | RMSEA | SRMR | CFI | TLI | AIC | χ^2^/*df* |
| --- | --- | --- | --- | --- | --- | --- | --- | --- |
| *Full sample* (*N* = 7,877) | | | | | | | | |
| Paired associate learning (linear) | 53.51 (33) | .013 | 0.01 | 0.01 | 0.99 | 0.99 | 66755.18 | 1.62 |
| Digit span test (linear) | 55.28 (33) | .009 | 0.01 | <0.01 | 1.00 | 0.99 | 88436.17 | 1.68 |
| Self-ordered search (linear) | 104.33 (33) | <.001 | 0.02 | 0.01 | 0.98 | 0.96 | 117728.68 | 3.16 |
| Verbal reasoning (quadratic) | 22.99 (15) | .084 | 0.01 | <0.01 | 1.00 | 1.00 | 172002.03 | 1.53 |
| *RTL subsample* (*n* = 846) | | | | | | | | |
| Paired associate learning (linear) | 36.99 (33) | .290 | 0.01 | 0.01 | 0.99 | 0.98 | 8457.48 | 1.12 |
| Digit span test (linear) | 25.15 (33) | .834 | <0.01 | 0.01 | 1.00 | 1.01 | 10755.17 | 0.76 |
| Self-ordered search (linear) | 40.68 (33) | .168 | 0.02 | 0.01 | 0.98 | 0.97 | 15338.46 | 1.23 |
| Verbal reasoning (quadratic) | 13.44 (15) | .568 | <0.01 | <0.01 | 1.00 | 1.00 | 22423.43 | 0.90 |

*Note.* χ^2^ = chi-square statistic; SRMR = Standardized Root Mean Squared Residual; RMSEA = root mean square error of approximation; CFI = comparative fit index; TLI = Tucker–Lewis index; AIC = Akaike Information Criterion. All models had freely estimated residual terms (heteroscedastic) and used full information maximum likelihood for missing data with robust maximum likelihood estimation (see Methods for more information).

**Supplementary Table 30. Fit indices for the conditional latent growth models for each of the cognitive outcomes, split by median age.**

| Model (growth shape) | χ2(df) | *p*-value | RMSEA | SRMR | CFI | TLI | AIC | χ2/df |
| --- | --- | --- | --- | --- | --- | --- | --- | --- |
|  | Full sample (*N* = 7,877; median age = ~62.24 years) | | | | | | | |
| Middle-aged subsample (n =3,938) | | | | | | | | |
| Paired associate learning (linear) | 53.8 (33) | .013 | 0.01 | 0.01 | 0.98 | 0.97 | 32961.07 | 1.63 |
| Digit span test (linear)^a^ | 40.1 (34) | .218 | 0.01 | 0.01 | 1.00 | 1.00 | 42371.88 | 1.18 |
| Self-ordered search (linear) | 110.69 (33) | .000 | 0.02 | 0.01 | 0.96 | 0.92 | 58630.85 | 3.35 |
| Verbal reasoning (quadratic) | 13.87 (15) | .536 | <0.01 | <0.01 | 1.00 | 1.00 | 86419.10 | 0.92 |
| Older subsample (n = 3,939) | | | | | | | | |
| Paired associate learning (linear) | 31.31 (33) | .552 | <0.01 | 0.01 | 1.00 | 1.00 | 33788.54 | 0.95 |
| Digit span test (linear) | 35.18 (33) | .365 | <0.01 | 0.01 | 1.00 | 1.00 | 45865.09 | 1.07 |
| Self-ordered search (linear) | 51.92 (33) | .019 | 0.01 | 0.01 | 0.99 | 0.98 | 59101.56 | 1.57 |
| Verbal reasoning (quadratic) | 21.17 (15) | .131 | 0.01 | <0.01 | 1.00 | 1.00 | 85553.56 | 1.41 |
|  | RTL Subsample (*n* = 846; median age = ~62.77 years) | | | | | | | |
| Middle-aged subsample (n = 423) | | | | | | | | |
| Paired associate learning (linear) | 37.91 (31) | .183 | <0.01 | 0.01 | 1.00 | 1.00 | 33788.54 | 0.95 |
| Digit span test (linear) | 38.03 (31) | .180 | <0.01 | 0.01 | 1.00 | 1.00 | 45865.09 | 1.07 |
| Self-ordered search (linear) | 45.77 (31) | .043 | 0.01 | 0.01 | 0.99 | 0.98 | 59101.56 | 1.57 |
| Verbal reasoning (quadratic) | 15.17 (14) | .367 | 0.01 | <0.01 | 1.00 | 1.00 | 85553.56 | 1.41 |
| Older subsample (n = 423) | | | | | | | | |
| Paired associate learning (linear) | 35.52 (31) | .264 | 0.02 | 0.02 | 0.98 | 0.96 | 4229.48 | 1.15 |
| Digit span test (linear) | 17.62 (31) | .974 | <0.01 | 0.01 | 1.00 | 1.04 | 5479.13 | 0.57 |
| Self-ordered search (linear) | 28.20 (31) | .611 | <0.01 | 0.02 | 1.00 | 1.03 | 7769.57 | 0.91 |
| Verbal reasoning (quadratic) | 18.33 (14) | .192 | 0.03 | 0.01 | 1.00 | 0.99 | 11107.26 | 1.31 |

*Note.* χ^2^ = chi-square statistic; SRMR = Standardized Root Mean Squared Residual; RMSEA = root mean square error of approximation; CFI = comparative fit index; TLI = Tucker–Lewis index; AIC = Akaike Information Criterion. All models had freely estimated residual terms (heteroscedastic) and used full information maximum likelihood for missing data with robust maximum likelihood estimation (see Methods for more information).

^a^ The variance for the slope variance was fixed to 0.0001 (i.e., estimated as a fixed effect; see Supplementary Methods S2), hence no confidence intervals are provided and no predictors are included.

**Supplementary Table 31. Parameter estimates [95% confidence intervals] for the conditional latent growth models for each of the cognitive outcomes (complete cases, *N* = 4,722).**

|  | Paired associated learning | Digit span test | Self-ordered search | Verbal reasoning |
| --- | --- | --- | --- | --- |
|  | Linear | Linear | Linear | Quadratic |
| Parameter | Estimate | Estimate | Estimate | Estimate |
| Means |  |  |  |  |
| Intercept | 5.96 [5.66, 6.26] | 9.20 [8.68, 9.72] | 11.80 [11.00, 12.61] | 47.88 [44.61, 51.14] |
| Slope | 0.07 [-0.06, 0.20] | 0.06 [-0.11, 0.23] | 0.24 [-0.09, 0.57] | 1.93 [-0.98, 4.83] |
| Slope^2^ | - | - | - | 0.21 [-0.67, 1.10] |
| Variances | | | | |
| Intercept | 0.25 [0.20, 0.29] | 1.30 [1.13, 1.48] | 2.06 [1.79, 2.34] | 59.59 [53.49, 65.70] |
| Slope | 0.01 [0.00, 0.02] | 0.03 [-0.00, 0.06] | 0.12 [0.05, 0.18] | 11.52 [4.79, 18.25] |
| Slope^2^ | - | - | - | 0.87 [0.43, 1.31] |
| Covariances | | | | |
| Intercept-Slope | -0.01 [-0.03, 0.00] | -0.04 [-0.09, 0.01] | -0.06 [-0.16, 0.05] | -0.36 [-6.72, 6.01] |
| Intercept-Slope^2^ | - | - | - | 0.35 [-1.21, 1.92] |
| Slope-Slope^2^ | - | - | - | -2.91 [-4.46, -1.36] |
| Residual variances | | | | |
| Baseline | 0.60 [0.54, 0.65] | 1.24 [1.04, 1.45] | 3.41 [3.06, 3.76] | 22.90 [17.13, 28.67] |
| Year 1 | 0.65 [0.61, 0.69] | 1.09 [0.95, 1.22] | 5.26 [4.85, 5.68] | 21.21 [19.12, 23.31] |
| Year 2 | 0.57 [0.53, 0.60] | 1.00 [0.88, 1.11] | 4.10 [3.75, 4.44] | 21.70 [19.50, 23.90] |
| Year 3 | 0.55 [0.50, 0.59] | 0.85 [0.70, 1.00] | 3.71 [3.32, 4.10] | 17.06 [10.62, 23.50] |
| Intercept predictors | | | | |
| PRS-TL | -0.01 [-0.03, 0.02] | -0.00 [-0.04, 0.04] | 0.00 [-0.06, 0.07] | 0.11 [-0.15, 0.37] |
| Age | -0.02 [-0.03, -0.02] | -0.03 [-0.04, -0.02] | -0.06 [-0.07, -0.05] | -0.33 [-0.38, -0.28] |
| Sex | 0.02 [-0.04, 0.07] | -0.19 [-0.29, -0.09] | -0.42 [-0.58, -0.26] | 0.37 [-0.25, 0.99] |
| Education | 0.03 [0.01, 0.04] | 0.09 [0.06, 0.12] | 0.11 [0.06, 0.16] | 1.32 [1.13, 1.51] |
| Employment (Full-time vs. Retired) | 0.04 [-0.04, 0.12] | 0.14 [-0.00, 0.27] | 0.18 [-0.04, 0.39] | 0.79 [-0.13, 1.71] |
| Employment (Full-time vs. Part-time | 0.01 [-0.08, 0.09] | 0.11 [-0.03, 0.25] | 0.20 [-0.02, 0.41] | 1.08 [0.13, 2.04] |
| Employment (Full-time vs. Self-employed) | 0.09 [0.00, 0.19] | 0.30 [0.14, 0.46] | 0.20 [-0.06, 0.46] | 1.82 [0.73, 2.90] |
| Employment (Full-time vs. Unemployed) | 0.08 [-0.07, 0.23] | -0.06 [-0.30, 0.19] | 0.43 [0.03, 0.84] | 1.42 [-0.32, 3.17] |
| PC1 | 1.00 [-1.16, 3.16] | -1.05 [-4.99, 2.89] | -9.32 [-15.06, -3.59] | -27.21 [-51.47, -2.95] |
| PC2 | -0.28 [-2.43, 1.86] | -2.03 [-5.94, 1.87] | 7.92 [1.98, 13.87] | 15.87 [-7.02, 38.76] |
| PC3 | 0.45 [-1.90, 2.79] | 0.13 [-3.87, 4.12] | 3.48 [-1.58, 8.53] | -5.65 [-28.15, 16.84] |
| PC4 | 0.57 [-1.55, 2.70] | -1.01 [-4.72, 2.70] | 4.04 [-1.24, 9.31] | -14.13 [-37.71, 9.45] |
| PC5 | -0.02 [-2.11, 2.06] | 0.19 [-3.44, 3.83] | 4.42 [-1.40, 10.23] | -8.24 [-29.14, 12.66] |
| PC6 | 1.37 [-0.53, 3.27] | 0.08 [-3.34, 3.50] | -1.47 [-7.16, 4.23] | -11.37 [-33.09, 10.34] |
| Slope predictors | | | | |
| PRS-TL | 0.00 [-0.01, 0.01] | 0.00 [-0.01, 0.02] | -0.02 [-0.04, 0.01] | -0.13 [-0.36, 0.10] |
| Age | -0.00 [-0.00, 0.00] | -0.00 [-0.00, 0.00] | -0.01 [-0.01, -0.00] | -0.01 [-0.06, 0.03] |
| Sex | 0.02 [-0.01, 0.04] | 0.03 [-0.00, 0.06] | 0.01 [-0.05, 0.08] | 0.91 [0.35, 1.47] |
| Education | 0.00 [-0.00, 0.01] | 0.01 [-0.00, 0.02] | 0.03 [0.01, 0.05] | 0.15 [-0.02, 0.32] |
| Employment (Full-time vs. Retired) | 0.00 [-0.03, 0.04] | 0.02 [-0.02, 0.07] | 0.03 [-0.06, 0.12] | 0.11 [-0.72, 0.94] |
| Employment (Full-time vs. Part-time | 0.02 [-0.02, 0.05] | 0.01 [-0.03, 0.06] | -0.01 [-0.10, 0.08] | -0.21 [-1.05, 0.63] |
| Employment (Full-time vs. Self-employed) | -0.02 [-0.06, 0.02] | 0.01 [-0.04, 0.07] | 0.02 [-0.09, 0.13] | -0.45 [-1.42, 0.52] |
| Employment (Full-time vs. Unemployed) | -0.04 [-0.11, 0.02] | 0.01 [-0.07, 0.09] | -0.05 [-0.19, 0.10] | -0.26 [-1.72, 1.19] |
| PC1 | -0.57 [-1.51, 0.36] | 0.37 [-0.90, 1.64] | 0.66 [-1.64, 2.97] | 5.39 [-14.92, 25.71] |
| PC2 | 0.58 [-0.32, 1.47] | 0.80 [-0.47, 2.07] | -1.81 [-4.16, 0.53] | 9.17 [-9.83, 28.16] |
| PC3 | -0.10 [-1.07, 0.87] | 0.71 [-0.91, 2.33] | 0.14 [-2.26, 2.55] | -0.74 [-18.00, 16.52] |
| PC4 | -0.10 [-1.01, 0.81] | -0.28 [-1.59, 1.03] | -0.45 [-2.66, 1.75] | 6.22 [-13.38, 25.81] |
| PC5 | -0.34 [-1.22, 0.53] | 0.33 [-0.91, 1.58] | 0.67 [-1.70, 3.04] | 10.61 [-8.83, 30.05] |
| PC6 | -0.63 [-1.43, 0.16] | -0.22 [-1.31, 0.87] | 1.29 [-0.85, 3.42] | -2.52 [-20.84, 15.80] |
| Slope^2^ predictors | | | | |
| PRS-TL | - | - | - | 0.04 [-0.03, 0.11] |
| Age | - | - | - | -0.01 [-0.02, 0.01] |
| Sex | - | - | - | -0.14 [-0.31, 0.03] |
| Education | - | - | - | -0.02 [-0.07, 0.03] |
| Employment (Full-time vs. Retired) | - | - | - | -0.06 [-0.32, 0.19] |
| Employment (Full-time vs. Part-time | - | - | - | -0.04 [-0.30, 0.21] |
| Employment (Full-time vs. Self-employed) | - | - | - | 0.09 [-0.20, 0.38] |
| Employment (Full-time vs. Unemployed) | - | - | - | -0.03 [-0.49, 0.43] |
| PC1 | - | - | - | -1.73 [-8.00, 4.54] |
| PC2 | - | - | - | -4.06 [-9.98, 1.85] |
| PC3 | - | - | - | -0.41 [-5.68, 4.86] |
| PC4 | - | - | - | -1.81 [-7.87, 4.25] |
| PC5 | - | - | - | -1.76 [-8.23, 4.72] |
| PC6 | - | - | - | 1.79 [-3.83, 7.40] |

*Note.* PRS-TL = polygenic score for telomere length (leukocyte); PC = genetic principal component*.* PRS-TL was standardised in the whole sample to a mean of 0 and SD of 1. All models had freely estimated residual terms (heteroscedastic) and used full information maximum likelihood for missing data with robust maximum likelihood estimation (see Methods).

**Supplementary Table 32. Parameter estimates [95% confidence intervals] for the conditional latent growth models for each of the cognitive outcomes complete cases aged under ~62.40 years at baseline (*N* = 2,361).**

|  | Paired associated learning | Digit span test | Self-ordered search | Verbal reasoning |
| --- | --- | --- | --- | --- |
|  | Linear | Linear | Linear | Quadratic |
| Parameter | Estimate | Estimate | Estimate | Estimate |
| Means |  |  |  |  |
| Intercept | 5.01 [4.41, 5.61] | 8.37 [7.33, 9.41] | 9.85 [8.18, 11.52] | 41.98 [35.01, 48.95] |
| Slope | 0.24 [-0.03, 0.50] | 0.05 [-0.28, 0.39] | 0.90 [0.24, 1.56] | 1.54 [-4.89, 7.97] |
| Slope^2^ | - | - | - | -0.02 [-1.99, 1.96] |
| Variances | | | | |
| Intercept | 0.28 [0.19, 0.36] | 1.13 [1.01, 1.25] | 2.12 [1.71, 2.53] | 64.21 [55.08, 73.33] |
| Slope | 0.02 [0.01, 0.03] | 0.00^a^ | 0.12 [0.03, 0.20] | 12.38 [2.57, 22.18] |
| Slope^2^ | - | - | - | 1.13 [0.49, 1.77] |
| Covariances | | | | |
| Intercept-Slope | -0.03 [-0.06, 0.00] | 0.00 [-0.02, 0.03] | -0.03 [-0.19, 0.12] | 1.06 [-8.20, 10.32] |
| Intercept-Slope^2^ | - | - | - | 0.11 [-2.17, 2.38] |
| Slope-Slope^2^ | - | - | - | -3.41 [-5.65, -1.16] |
| Residual variances | | | | |
| Baseline | 0.58 [0.51, 0.66] | 1.23 [0.98, 1.49] | 3.31 [2.80, 3.83] | 24.64 [16.14, 33.14] |
| Year 1 | 0.64 [0.58, 0.70] | 0.90 [0.79, 1.01] | 5.73 [5.08, 6.37] | 22.44 [19.38, 25.51] |
| Year 2 | 0.55 [0.51, 0.60] | 0.89 [0.77, 1.02] | 3.87 [3.39, 4.36] | 23.11 [19.88, 26.34] |
| Year 3 | 0.56 [0.50, 0.62] | 0.86 [0.70, 1.01] | 3.34 [2.79, 3.89] | 16.00 [6.46, 25.53] |
| Intercept predictors | | | | |
| PRS-TL | -0.01 [-0.04, 0.02] | -0.01 [-0.07, 0.04] | 0.01 [-0.08, 0.10] | 0.12 [-0.26, 0.50] |
| Age | -0.01 [-0.02, 0.00] | -0.01 [-0.03, 0.00] | -0.03 [-0.06, -0.00] | -0.24 [-0.36, -0.12] |
| Sex | 0.00 [-0.08, 0.09] | -0.21 [-0.35, -0.06] | -0.38 [-0.63, -0.13] | 0.36 [-0.62, 1.34] |
| Education | 0.03 [0.00, 0.06] | 0.10 [0.05, 0.14] | 0.13 [0.06, 0.20] | 1.42 [1.13, 1.72] |
| Employment (Full-time vs. Retired) | 0.08 [-0.02, 0.18] | 0.22 [0.05, 0.38] | 0.13 [-0.13, 0.39] | 0.98 [-0.16, 2.12] |
| Employment (Full-time vs. Part-time | 0.02 [-0.07, 0.11] | 0.09 [-0.06, 0.24] | 0.19 [-0.05, 0.42] | 1.27 [0.21, 2.34] |
| Employment (Full-time vs. Self-employed) | 0.09 [-0.02, 0.19] | 0.24 [0.06, 0.42] | 0.17 [-0.12, 0.47] | 1.93 [0.71, 3.16] |
| Employment (Full-time vs. Unemployed) | 0.10 [-0.06, 0.25] | -0.02 [-0.27, 0.23] | 0.42 [0.01, 0.83] | 1.98 [0.20, 3.77] |
| PC1 | 0.78 [-2.22, 3.77] | 0.48 [-4.48, 5.43] | -10.98 [-19.04, -2.93] | -26.60 [-60.17, 6.96] |
| PC2 | -0.73 [-3.39, 1.93] | -3.12 [-8.55, 2.31] | 9.27 [0.67, 17.86] | 15.99 [-15.69, 47.67] |
| PC3 | -2.13 [-4.63, 0.38] | -0.51 [-6.01, 4.99] | 4.61 [-1.96, 11.17] | -7.27 [-37.98, 23.43] |
| PC4 | -0.16 [-3.21, 2.89] | -0.26 [-4.96, 4.43] | 1.92 [-5.34, 9.18] | -8.23 [-42.70, 26.23] |
| PC5 | -0.73 [-3.23, 1.76] | -0.55 [-4.50, 3.40] | 1.34 [-6.15, 8.83] | 11.00 [-17.46, 39.46] |
| PC6 | 1.89 [-0.86, 4.65] | 1.59 [-3.46, 6.65] | -4.06 [-12.24, 4.13] | 2.40 [-28.51, 33.31] |
| Slope predictors | | | | |
| PRS-TL | 0.00 [-0.01, 0.02] | 0.02 [-0.00, 0.04] | -0.01 [-0.05, 0.02] | 0.16 [-0.17, 0.50] |
| Age | -0.00 [-0.01, 0.00] | -0.00 [-0.01, 0.00] | -0.01 [-0.02, -0.00] | 0.00 [-0.10, 0.11] |
| Sex | 0.01 [-0.02, 0.05] | 0.01 [-0.03, 0.06] | -0.12 [-0.22, -0.02] | 0.87 [-0.04, 1.78] |
| Education | 0.01 [-0.00, 0.02] | 0.01 [-0.00, 0.02] | 0.03 [0.00, 0.06] | 0.01 [-0.24, 0.26] |
| Employment (Full-time vs. Retired) | -0.01 [-0.05, 0.04] | 0.02 [-0.04, 0.07] | 0.08 [-0.03, 0.18] | -0.01 [-1.05, 1.04] |
| Employment (Full-time vs. Part-time | 0.00 [-0.04, 0.04] | 0.03 [-0.02, 0.07] | -0.01 [-0.11, 0.09] | -0.23 [-1.15, 0.70] |
| Employment (Full-time vs. Self-employed) | -0.01 [-0.06, 0.03] | 0.03 [-0.03, 0.10] | 0.04 [-0.08, 0.15] | -0.66 [-1.75, 0.43] |
| Employment (Full-time vs. Unemployed) | -0.05 [-0.11, 0.02] | 0.00 [-0.08, 0.08] | -0.02 [-0.17, 0.13] | -0.39 [-1.90, 1.11] |
| PC1 | -0.34 [-1.65, 0.97] | 0.22 [-1.38, 1.83] | 1.65 [-1.44, 4.75] | 8.87 [-19.11, 36.84] |
| PC2 | 1.62 [0.45, 2.79] | 1.40 [-0.31, 3.11] | -0.86 [-4.00, 2.29] | 10.80 [-15.87, 37.46] |
| PC3 | 0.25 [-1.05, 1.55] | 0.11 [-2.17, 2.38] | -1.39 [-4.25, 1.48] | 4.15 [-18.87, 27.17] |
| PC4 | -0.14 [-1.45, 1.17] | -0.23 [-1.75, 1.29] | 0.65 [-2.33, 3.63] | 19.85 [-7.35, 47.05] |
| PC5 | -0.58 [-1.70, 0.54] | 0.50 [-0.81, 1.80] | 0.05 [-2.79, 2.89] | 2.84 [-23.21, 28.89] |
| PC6 | -0.74 [-1.85, 0.36] | -0.40 [-1.88, 1.08] | 1.27 [-1.81, 4.36] | -3.62 [-30.58, 23.33] |
| Slope^2^ predictors | | | | |
| PRS-TL | - | - | - | -0.04 [-0.14, 0.07] |
| Age | - | - | - | -0.01 [-0.04, 0.03] |
| Sex | - | - | - | -0.09 [-0.38, 0.19] |
| Education | - | - | - | 0.03 [-0.05, 0.11] |
| Employment (Full-time vs. Retired) | - | - | - | -0.02 [-0.33, 0.30] |
| Employment (Full-time vs. Part-time | - | - | - | -0.05 [-0.34, 0.24] |
| Employment (Full-time vs. Self-employed) | - | - | - | 0.15 [-0.18, 0.48] |
| Employment (Full-time vs. Unemployed) | - | - | - | 0.02 [-0.46, 0.49] |
| PC1 | - | - | - | -4.84 [-13.39, 3.72] |
| PC2 | - | - | - | -5.66 [-13.64, 2.32] |
| PC3 | - | - | - | -0.92 [-7.81, 5.96] |
| PC4 | - | - | - | -6.07 [-14.57, 2.43] |
| PC5 | - | - | - | 1.45 [-7.17, 10.08] |
| PC6 | - | - | - | 1.87 [-6.30, 10.05] |

*Note.* PRS-TL = polygenic score for telomere length (leukocyte); PC = genetic principal component*.* PRS-TL was standardised in the whole sample to a mean of 0 and SD of 1. All models had freely estimated residual terms (heteroscedastic) and used full information maximum likelihood for missing data with robust maximum likelihood estimation (see Methods).

^a^ For the digit span test model, the variance for the slope variance was fixed to 0.0001 (i.e., estimated as a fixed effect; see Supplementary Methods S2), hence no confidence intervals are provided.

**Supplementary Table 33. Parameter estimates [95% confidence intervals] for the conditional latent growth models for each of the cognitive outcomes in complete cases aged 62.40 years and over at baseline (*N* = 2,361).**

|  | Paired associated learning | Digit span test | Self-ordered search | Verbal reasoning |
| --- | --- | --- | --- | --- |
|  | Linear | Linear | Linear | Quadratic |
| Parameter | Estimate | Estimate | Estimate | Estimate |
| Means |  |  |  |  |
| Intercept | 6.30 [5.77, 6.84] | 9.96 [8.97, 10.94] | 13.46 [11.98, 14.93] | 57.28 [51.32, 63.25] |
| Slope | 0.12 [-0.11, 0.36] | 0.21 [-0.13, 0.55] | -0.34 [-0.97, 0.30] | 2.33 [-2.76, 7.42] |
| Slope^2^ | - | - | - | 0.43 [-1.11, 1.98] |
| Variances | | | | |
| Intercept | 0.21 [0.16, 0.25] | 1.49 [1.19, 1.79] | 2.00 [1.64, 2.36] | 54.05 [46.02, 62.08] |
| Slope | 0.01 [-0.00, 0.02] | 0.06 [0.03, 0.10] | 0.11 [0.02, 0.20] | 10.32 [1.13, 19.50] |
| Slope^2^ | - | - | - | 0.57 [-0.04, 1.19] |
| Covariances | | | | |
| Intercept-Slope | 0.00 [-0.02, 0.02] | -0.10 [-0.18, -0.02] | -0.08 [-0.23, 0.07] | -1.73 [-10.41, 6.94] |
| Intercept-Slope^2^ | - | - | - | 0.56 [-1.58, 2.70] |
| Slope-Slope^2^ | - | - | - | -2.31 [-4.44, -0.17] |
| Residual variances | | | | |
| Baseline | 0.61 [0.54, 0.67] | 1.22 [0.99, 1.45] | 3.49 [3.01, 3.97] | 21.16 [13.39, 28.92] |
| Year 1 | 0.66 [0.61, 0.72] | 1.27 [1.03, 1.51] | 4.79 [4.28, 5.31] | 19.95 [17.11, 22.79] |
| Year 2 | 0.59 [0.53, 0.64] | 1.11 [0.92, 1.30] | 4.31 [3.82, 4.80] | 20.32 [17.35, 23.28] |
| Year 3 | 0.53 [0.47, 0.60] | 0.81 [0.63, 0.98] | 4.10 [3.54, 4.66] | 18.23 [9.69, 26.77] |
| Intercept predictors | | | | |
| PRS-TL | -0.01 [-0.04, 0.03] | 0.01 [-0.05, 0.06] | 0.00 [-0.08, 0.09] | 0.09 [-0.25, 0.44] |
| Age | -0.03 [-0.03, -0.02] | -0.04 [-0.05, -0.02] | -0.08 [-0.10, -0.06] | -0.42 [-0.50, -0.35] |
| Sex | 0.02 [-0.05, 0.09] | -0.18 [-0.31, -0.04] | -0.45 [-0.66, -0.25] | 0.26 [-0.54, 1.06] |
| Education | 0.02 [-0.00, 0.05] | 0.08 [0.04, 0.13] | 0.09 [0.03, 0.15] | 1.23 [0.99, 1.48] |
| Employment (Full-time vs. Retired) | -0.17 [-0.35, 0.00] | -0.09 [-0.44, 0.27] | -0.02 [-0.54, 0.51] | -1.89 [-4.10, 0.32] |
| Employment (Full-time vs. Part-time | -0.21 [-0.42, 0.00] | -0.00 [-0.42, 0.42] | -0.03 [-0.62, 0.57] | -1.43 [-3.93, 1.07] |
| Employment (Full-time vs. Self-employed) | -0.06 [-0.28, 0.15] | 0.27 [-0.15, 0.70] | 0.06 [-0.59, 0.71] | -0.54 [-3.18, 2.10] |
| Employment (Full-time vs. Unemployed) | -0.17 [-1.00, 0.66] | -0.52 [-1.92, 0.88] | 1.05 [-1.32, 3.41] | -7.31 [-17.19, 2.57] |
| PC1 | 0.98 [-2.15, 4.12] | -3.60 [-9.89, 2.68] | -7.78 [-15.86, 0.29] | -29.72 [-64.61, 5.18] |
| PC2 | -0.01 [-3.43, 3.41] | -1.07 [-6.60, 4.46] | 7.82 [0.13, 15.50] | 13.76 [-18.28, 45.80] |
| PC3 | 4.34 [0.12, 8.56] | 1.22 [-3.94, 6.39] | 1.42 [-6.22, 9.05] | -1.36 [-32.56, 29.83] |
| PC4 | 1.69 [-1.25, 4.62] | -1.64 [-7.45, 4.16] | 6.47 [-1.05, 13.99] | -20.93 [-52.97, 11.12] |
| PC5 | 1.04 [-2.48, 4.56] | 1.20 [-5.52, 7.93] | 8.14 [-0.01, 16.28] | -33.77 [-65.30, -2.24] |
| PC6 | 0.16 [-2.61, 2.93] | -1.83 [-6.69, 3.04] | 0.43 [-6.72, 7.57] | -23.55 [-53.11, 6.01] |
| Slope predictors | | | | |
| PRS-TL | 0.00 [-0.01, 0.01] | -0.01 [-0.03, 0.01] | -0.03 [-0.06, 0.01] | -0.43 [-0.74, -0.11] |
| Age | -0.00 [-0.01, 0.00] | -0.00 [-0.01, 0.00] | -0.00 [-0.01, 0.01] | -0.03 [-0.09, 0.04] |
| Sex | 0.02 [-0.01, 0.05] | 0.04 [-0.00, 0.09] | 0.11 [0.02, 0.20] | 0.94 [0.23, 1.65] |
| Education | 0.00 [-0.01, 0.01] | 0.00 [-0.01, 0.02] | 0.03 [0.00, 0.06] | 0.25 [0.02, 0.48] |
| Employment (Full-time vs. Retired) | 0.04 [-0.04, 0.12] | -0.03 [-0.15, 0.09] | 0.06 [-0.18, 0.29] | 0.45 [-1.62, 2.51] |
| Employment (Full-time vs. Part-time | 0.10 [0.01, 0.19] | -0.06 [-0.19, 0.07] | 0.14 [-0.12, 0.39] | 0.05 [-2.29, 2.39] |
| Employment (Full-time vs. Self-employed) | -0.01 [-0.10, 0.09] | -0.08 [-0.22, 0.06] | 0.04 [-0.27, 0.34] | 0.32 [-2.13, 2.77] |
| Employment (Full-time vs. Unemployed) | 0.11 [-0.30, 0.51] | 0.45 [-0.05, 0.95] | 0.05 [-0.35, 0.45] | 0.93 [-5.29, 7.15] |
| PC1 | -0.84 [-2.17, 0.48] | 0.66 [-1.34, 2.66] | -0.68 [-4.14, 2.78] | 2.63 [-27.18, 32.45] |
| PC2 | -0.56 [-1.86, 0.75] | 0.18 [-1.73, 2.10] | -3.16 [-6.62, 0.30] | 9.69 [-18.10, 37.49] |
| PC3 | -0.43 [-1.79, 0.93] | 1.83 [-0.14, 3.80] | 2.65 [-1.17, 6.46] | -6.56 [-32.59, 19.47] |
| PC4 | -0.08 [-1.35, 1.20] | -0.29 [-2.51, 1.93] | -1.64 [-4.81, 1.53] | -9.69 [-38.16, 18.77] |
| PC5 | -0.14 [-1.55, 1.28] | -0.04 [-2.41, 2.32] | 1.27 [-2.64, 5.19] | 19.70 [-8.96, 48.36] |
| PC6 | -0.74 [-1.92, 0.43] | -0.17 [-1.95, 1.60] | 0.91 [-2.17, 3.98] | -0.00 [-25.20, 25.20] |
| Slope^2^ predictors | | | | |
| PRS-TL | - | - | - | 0.12 [0.02, 0.21] |
| Age | - | - | - | -0.00 [-0.02, 0.02] |
| Sex | - | - | - | -0.18 [-0.39, 0.04] |
| Education | - | - | - | -0.06 [-0.13, 0.00] |
| Employment (Full-time vs. Retired) | - | - | - | -0.23 [-0.84, 0.38] |
| Employment (Full-time vs. Part-time | - | - | - | -0.16 [-0.86, 0.54] |
| Employment (Full-time vs. Self-employed) | - | - | - | -0.19 [-0.91, 0.53] |
| Employment (Full-time vs. Unemployed) | - | - | - | -0.59 [-2.34, 1.16] |
| PC1 | - | - | - | 1.63 [-7.66, 10.92] |
| PC2 | - | - | - | -2.75 [-11.64, 6.14] |
| PC3 | - | - | - | -0.37 [-8.32, 7.58] |
| PC4 | - | - | - | 3.04 [-5.66, 11.74] |
| PC5 | - | - | - | -5.74 [-14.54, 3.06] |
| PC6 | - | - | - | 1.96 [-5.66, 9.58] |

*Note.* PRS-TL = polygenic score for telomere length (leukocyte); PC = genetic principal component*.* PRS-TL was standardised in the whole sample to a mean of 0 and SD of 1. All models had freely estimated residual terms (heteroscedastic) and used full information maximum likelihood for missing data with robust maximum likelihood estimation (see Methods).

**Supplementary Table 34. Fit indices for the conditional latent growth models for each of the cognitive outcomes in complete cases and split by median age.**

| Model (growth shape) | χ2(df) | *p*-value | | RMSEA | SRMR | CFI | TLI | AIC | χ2/df |
| --- | --- | --- | --- | --- | --- | --- | --- | --- | --- |
|  | Full sample (*N* = 4,722; median age = ~62.40 years) | | | | | | | | |
| Paired associate learning (linear) | 49.57 (33) | .032 | 0.01 | | 0.01 | 0.99 | 0.99 | 48780.53 | 1.50 |
| Digit span test (linear) | 62.35 (33) | .002 | 0.01 | | 0.01 | 0.99 | 0.99 | 63269.42 | 1.89 |
| Self-ordered search (linear) | 73.35 (33) | .000 | 0.02 | | 0.01 | 0.99 | 0.98 | 86253.01 | 2.22 |
| Verbal reasoning (quadratic) | 24.27 (15) | .000 | 0.01 | | <0.01 | 1.00 | 1.00 | 125742.43 | 1.62 |
| Middle-aged subsample (n = 2,361) | | | | | | | | | |
| Paired associate learning (linear) | 50.09 (33) | .029 | | 0.02 | 0.01 | 0.98 | 0.97 | 24395.61 | 1.52 |
| Digit span test (linear)^a^ | 45.62 (34) | .088 | | 0.01 | 0.01 | 1.00 | 0.99 | 30629.45 | 1.34 |
| Self-ordered search (linear) | 80.86 (33) | <.001 | | 0.03 | 0.01 | 0.97 | 0.94 | 43089.40 | 2.45 |
| Verbal reasoning (quadratic) | 13.42 (15) | .570 | | 0.00 | 0.00 | 1.00 | 1.00 | 63565.69 | 0.90 |
| Older subsample (n = 2,361) | | | | | | | | | |
| Paired associate learning (linear) | 30.16 (33) | .609 | | 0.00 | 0.01 | 1.00 | 1.01 | 24384.33 | 0.91 |
| Digit span test (linear) | 43.43 (33) | .106 | | 0.01 | 0.01 | 1.00 | 0.99 | 32539.16 | 1.32 |
| Self-ordered search (linear) | 50.07 (33) | .029 | | 0.02 | 0.01 | 0.99 | 0.98 | 43157.42 | 1.52 |
| Verbal reasoning (quadratic) | 17.95 (15) | .265 | | 0.01 | 0.00 | 1.00 | 1.00 | 62158.47 | 1.20 |

*Note.* χ^2^ = chi-square statistic; SRMR = Standardized Root Mean Squared Residual; RMSEA = root mean square error of approximation; CFI = comparative fit index; TLI = Tucker–Lewis index; AIC = Akaike Information Criterion. All models had freely estimated residual terms (heteroscedastic) and used full information maximum likelihood for missing data with robust maximum likelihood estimation (see Methods for more information).

^a^ The variance for the slope variance was fixed to 0.0001 (i.e., estimated as a fixed effect; see Supplementary Methods S2), hence no confidence intervals are provided and no predictors are included.
